# Supplementary material for: The Relationship of Tree Nuts and Peanuts with Adiposity Parameters: A Systematic Review and Network Meta-Analysis
Source: Nutrients. 2021 Jun 30;13(7):2251. doi: 10.3390/nu13072251 (PMC8308485; doi:10.3390/nu13072251)

Supplementary Material

The relationship of tree nuts and peanuts with adiposity parameters: A systematic review and network meta-analysis.

Rubén Fernández-Rodríguez (Ruben.Fernandez@uclm.es), MSc[1] ; Arthur E. Mesas (Arthur.EMesas@uclm.es), PhD[1,2*]; Miriam Garrido-Miguel (Miriam.Garrido@uclm.es), PhD[1,3]; Isabel A. Martínez-Ortega (IsabelA.Martinez@uclm.es), MSc[1]; Estela Jiménez-López (Estela.JimenezLopez@uclm.es), PhD[1]; Vicente Martínez-Vizcaíno (Vicente.Martinez@uclm.es), PhD, MD[1,4].

1 Universidad de Castilla La-Mancha, Health and Social Research Center, Cuenca, Spain.

2 Universidade Estadual de Londrina, Health Science Centre, Londrina, Brazil.

3 Universidad de Castilla-La Mancha, Facultad de Enfermería, Albacete, Spain.

4 Universidad Autónoma de Chile, Facultad de Ciencias de la Salud, Talca, Chile.

***Correspondence**: Arthur.EMesas@uclm.es, Universidad de Castilla La-Mancha, Health and Social Research Center, Santa Teresa Jornet s/n, 16071 Cuenca, Spain, +(34) 969179100 ext. 4686

INDEX

PRISMA NMA Checklist......3

Table S1. Search strategy for databases......7

Table S2. Excluded studies with reasons (n=140)......8

Table S3. Characteristics of the included studies......18

Table S4. Transitivity assessment......33

Table S5. Transitivity assessment regarding the Intervention groups......34

Table S6. Transitivity assessment regarding the Control groups......38

Table S7. Direct and indirect evidence and network meta-analysis results summary table for body weight using CINeMA......42

Table S8. Direct and indirect evidence and network meta-analysis results summary table for body mass index using CINeMA......44

Table S9. Direct and indirect evidence and network meta-analysis results summary table for waist circumference using CINeMA......47

Table S10. Direct and indirect evidence and network meta-analysis results summary table for body fat percentage using CINeMA......49

Table S11. Quality Assessment (RoB 2.0)......51

Table S12. Percentage of change for healthy/normal weight and people with Overweight/obesity......58

Table S13. GRADE approach: summary of findings......60

Table S14. CINeMA final report......62

Table S15. Sensitivity analyses......70

Table S16. Publication bias......75

Table S17. Inconsistency assessment......76

Figure S1. Network diagrams......77

Figure S2. Pairwise meta-analyses for all outcomes......78

Figure S3. Network estimates for body weight......79

Figure S4. Subgroup analyses......83

Figure S5. Subgroup analyses based on control comparison for all outcomes:......91

Figure S6. Meta-regressions......92

Figure S7. Funnel plots......94

Figure S8. Risk of Bias assessment......96

PRISMA NMA Checklist of Items to Include When Reporting a Systematic Review Involving a Network Meta-analysis

Section/Topic	Item #	Checklist Item	Reported on Page #
**TITLE**			
Title	1	Identify the report as a systematic review *incorporating a network meta-analysis (or related form of meta-analysis).*	***1***
**ABSTRACT**			***2***
Structured summary	2	Provide a structured summary including, as applicable:<br>**Background:** main objectives<br>**Methods:** data sources; study eligibility criteria, participants, and interventions; study appraisal; and *synthesis methods, such as network meta-analysis.*<br>**Results:** number of studies and participants identified; summary estimates with corresponding confidence/credible intervals; *treatment rankings may also be discussed. Authors may choose to summarize pairwise comparisons against a chosen treatment included in their analyses for brevity.*<br>**Discussion/Conclusions:** limitations; conclusions and implications of findings.<br>**Other:** primary source of funding; systematic review registration number with registry name.	
**INTRODUCTION**			
Rationale	3	Describe the rationale for the review in the context of what is already known*, including mention of why a network meta-analysis has been conducted.*	***3***
Objectives	4	Provide an explicit statement of questions being addressed, with reference to participants, interventions, comparisons, outcomes, and study design (PICOS).	4
**METHODS**			
Protocol and registration	5	Indicate whether a review protocol exists and if and where it can be accessed (e.g., Web address); and, if available, provide registration information, including registration number.	4
Eligibility criteria	6	Specify study characteristics (e.g., PICOS, length of follow-up) and report characteristics (e.g., years considered, language, publication status) used as criteria for eligibility, giving rationale. *Clearly describe eligible treatments included in the treatment network, and note whether any have*	***4-5***

		been clustered or merged into the same node (with justification).	
Information sources	7	Describe all information sources (e.g., databases with dates of coverage, contact with study authors to identify additional studies) in the search and date last searched.	4
Search	8	Present full electronic search strategy for at least one database, including any limits used, such that it could be repeated.	4
Study selection	9	State the process for selecting studies (i.e., screening, eligibility, included in systematic review, and, if applicable, included in the meta-analysis).	5
Data collection process	10	Describe method of data extraction from reports (e.g., piloted forms, independently, in duplicate) and any processes for obtaining and confirming data from investigators.	5
Data items	11	List and define all variables for which data were sought (e.g., PICOS, funding sources) and any assumptions and simplifications made.	5
**Geometry of the network**	**S1**	Describe methods used to explore the geometry of the treatment network under study and potential biases related to it. This should include how the evidence base has been graphically summarized for presentation, and what characteristics were compiled and used to describe the evidence base to readers.	***6-7***
Risk of bias within individual studies	12	Describe methods used for assessing risk of bias of individual studies (including specification of whether this was done at the study or outcome level), and how this information is to be used in any data synthesis.	6
Summary measures	13	State the principal summary measures (e.g., risk ratio, difference in means). *Also describe the use of additional summary measures assessed, such as treatment rankings and surface under the cumulative ranking curve (SUCRA) values, as well as modified approaches used to present summary findings from meta-analyses.*	6-7
Planned methods of analysis	14	Describe the methods of handling data and combining results of studies for each network meta-analysis. This should include, but not be limited to:<br>• *Handling of multi-arm trials;*<br>• *Selection of variance structure;*<br>• *Selection of prior distributions in Bayesian analyses; and*<br>• *Assessment of model fit.*	6-8
**Assessment of Inconsistency**	**S2**	Describe the statistical methods used to evaluate the agreement of direct and indirect evidence in the treatment network(s) studied. Describe efforts taken to address its presence when found.	8
Risk of bias across studies	15	Specify any assessment of risk of bias that may affect the cumulative evidence (e.g., publication bias, selective reporting within studies).	**8**

Additional analyses	16	Describe methods of additional analyses if done, indicating which were pre-specified. This may include, but not be limited to, the following:<br>• Sensitivity or subgroup analyses;<br>• Meta-regression analyses;<br>• *Alternative formulations of the treatment network; and*<br>• *Use of alternative prior distributions for Bayesian analyses (if applicable).*	***7-8***
**RESULTS†**			
Study selection	17	Give numbers of studies screened, assessed for eligibility, and included in the review, with reasons for exclusions at each stage, ideally with a flow diagram.	8
**Presentation of network structure**	**S3**	Provide a network graph of the included studies to enable visualization of the geometry of the treatment network.	***9***
**Summary of network geometry**	**S4**	Provide a brief overview of characteristics of the treatment network. This may include commentary on the abundance of trials and randomized patients for the different interventions and pairwise comparisons in the network, gaps of evidence in the treatment network, and potential biases reflected by the network structure.	***8-9***
Study characteristics	18	For each study, present characteristics for which data were extracted (e.g., study size, PICOS, follow-up period) and provide the citations.	8-9
Risk of bias within studies	19	Present data on risk of bias of each study and, if available, any outcome level assessment.	9
Results of individual studies	20	For all outcomes considered (benefits or harms), present, for each study: 1) simple summary data for each intervention group, and 2) effect estimates and confidence intervals. *Modified approaches may be needed to deal with information from larger networks.*	8-10
Synthesis of results	21	Present results of each meta-analysis done, including confidence/credible intervals. *In larger networks, authors may focus on comparisons versus a particular comparator (e.g. placebo or standard care), with full findings presented in an appendix. League tables and forest plots may be considered to summarize pairwise comparisons.* If additional summary measures were explored (such as treatment rankings), these should also be presented.	***Table1, supplementary material***
**Exploration for inconsistency**	**S5**	Describe results from investigations of inconsistency. This may include such information as measures of model fit to compare consistency	***9***

		and inconsistency models, *P* values from statistical tests, or summary of inconsistency estimates from different parts of the treatment network.	
Risk of bias across studies	22	Present results of any assessment of risk of bias across studies for the evidence base being studied.	9-10
Results of additional analyses	23	Give results of additional analyses, if done (e.g., sensitivity or subgroup analyses, meta-regression analyses*, alternative network geometries studied, alternative choice of prior distributions for Bayesian analyses,* and so forth).	***10-11***
**DISCUSSION**			
Summary of evidence	24	Summarize the main findings, including the strength of evidence for each main outcome; consider their relevance to key groups (e.g., healthcare providers, users, and policy-makers).	11
Limitations	25	Discuss limitations at study and outcome level (e.g., risk of bias), and at review level (e.g., incomplete retrieval of identified research, reporting bias). *Comment on the validity of the assumptions, such as transitivity and consistency. Comment on any concerns regarding network geometry (e.g., avoidance of certain comparisons).*	13
Conclusions	26	Provide a general interpretation of the results in the context of other evidence, and implications for future research.	13-14
**FUNDING**			
Funding	27	Describe sources of funding for the systematic review and other support (e.g., supply of data); role of funders for the systematic review. This should also include information regarding whether funding has been received from manufacturers of treatments in the network and/or whether some of the authors are content experts with professional conflicts of interest that could affect use of treatments in the network.	***14***

PICOS = population, intervention, comparators, outcomes, study design.
* Text in italics indicateS wording specific to reporting of network meta-analyses that has been added to guidance from the PRISMA statement.
† Authors may wish to plan for use of appendices to present all relevant information in full detail for items in this section.

Table S1. Search strategy for databases.

Search set Medline
#1 nut [All Fields]
#2 tree nut [MeSH Terms]
#3 almond [All Fields]
#4 hazelnut [All Fields]
#5 pistachio [All Fields]
#6 walnut [All Fields]
#7 cashew [All Fields]
#8 peanut [All Fields]
#9 nuts [All Fields]
#10 almonds [All Fields]
#11 hazelnuts [All Fields]
#11 pistachios [All Fields]
#12 walnuts [All Fields]
#13 cashews [All Fields]
#14 peanut [All Fields]
#15 anthropometric [All Fields]
#16 weight [All Fields]
#17 body weight [All Fields]
#18 fat [All Fields]
#19 fat mass [All Fields]
#20 fat percentage [All Fields]
#21 BMI [All Fields]
#22 body mass index [All Fields]
#23 waist circumference [All Fields]
#24 obese [All Fields]

Search set Medline
#25 obesity [All Fields]
#26 overweight [All Fields]
#27 over weighted [All Fields]
#28 body fat [All Fields]
#29 RCT [All Fields]
#30 randomised controlled trial [All Fields]
#31 controlled trial [All Fields]

Search set Medline
#32 randomized controlled trial [All Fields]
#33 trial [All Fields]
#34 clinical trial [All Fields]
#35 intervention [All Fields]
#36 1 OR 2 OR 3 OR 4 OR 5 OR 6 OR 7 OR 8 OR 9 OR 10 OR 11 OR 12 OR 13 OR 14
#37 15 OR 16 OR 17 OR 18 OR 19 OR 20 OR 21 OR 22 OR 23 OR 24 OR 25 OR 26 OR 27 OR 28
#38 29 OR 30 OR 31 OR 32 OR 33 OR 34 OR 35
#39 36 AND 37 AND 38

SCOPUS search strategy
(TITLE-ABS-KEY ((nut OR almond OR hazelnut OR pistachio OR walnut OR cashew, OR nuts OR almonds OR hazelnuts OR pistachios OR walnuts OR cashews))) AND (TITLE-ABS-KEY ((anthropometric OR weight OR "body weight" OR "fat" OR "fat mass" OR "fat percentage" OR bmi OR "body mass index" OR "waist circumference" OR "obese" OR "obesity" OR "overweight" OR "over weighted" OR "body fat"))) AND (TITLE-ABS-KEY ((rct OR "randomised controlled trial" OR "controlled trial" OR "randomized controlled trial" OR "trial" OR "clinical trial" OR intervention)))

WOS search strategy
(nut OR almond OR hazelnut OR pistachio OR walnut OR cashew, OR nuts OR almonds OR hazelnuts OR pistachios OR walnuts OR cashews) AND (anthropometric OR weight OR “body weight” OR “fat” OR “fat mass” OR “fat percentage” OR BMI OR “body mass index” OR “waist circumference” OR “obese” OR “obesity” OR “overweight” OR “over weighted” OR “body fat”) AND (RCT OR "randomised controlled trial" OR "controlled trial" OR "randomized controlled trial" OR “trial” OR “clinical trial” OR Intervention)

Table S2. Excluded studies with reasons (n=140).

<u>Studies excluded after full-text review</u>
**Non-eligible publications:**
1. Arab L, Dhaliwal SK, Martin CJ, Larios AD, Jackson NJ, Elashoff D. Association between walnut consumption and diabetes risk in NHANES. Diabetes Metab Res Rev. 2018;34(7):1-8. doi:10.1002/dmrr.3031
2. Bibiloni MDM, Julibert A, Bouzas C, et al. Nut consumptions as a marker of higher diet quality in a mediterranean population at high cardiovascular risk. Nutrients. 2019;11(4). doi:10.3390/nu11040754
3. Campbell 2019 (abstract-> excluded) Almond Consumption Increases Satiety Hormones Relative to a High-Carbohydrate Food but Has Minimal Impact on Body Composition: A Pilot Study in Black and Hispanic Adults
4. Casas-Agustench P, Bulló M, Ros E, Basora J, Salas-Salvadó J. Cross-sectional association of nut intake with adiposity in a Mediterranean population. Nutr Metab Cardiovasc Dis. 2011;21(7):518-525. doi:10.1016/j.numecd.2009.11.010
5. Chen et al 2011. Almonds decreased inflammation and oxidative stress in patients with type 2 diabetes
6. De La Torre NG, Assaf-Balut C, Varas IJ, et al. Effectiveness of following mediterranean diet recommendations in the real world in the incidence of gestational diabetes mellitus (Gdm) and adverse maternal-foetal outcomes: A prospective, universal, interventional study with a single group. the st carlos study. Nutrients. 2019;11(6). doi:10.3390/nu11061210
7. Dikariyanto V, Berry SE, Francis L, Smith L, Hall WL. Whole almond consumption is associated with better diet quality and cardiovascular disease risk factors in the UK adult population: National Diet and Nutrition Survey (NDNS) 2008–2017. Eur J Nutr. Published online 2020. doi:10.1007/s00394-020-02270-9
8. Djoussé L, Gaziano JM, Kase CS, Kurth T. Nut consumption and risk of stroke in US male physicians. Clin Nutr. 2010;29(5):605-609. doi:10.1016/j.clnu.2010.03.005
9. Ibarrola-Jurado N, Bulló M, Guasch-Ferré M, et al. Cross-Sectional Assessment of Nut Consumption and Obesity, Metabolic Syndrome and Other Cardiometabolic Risk Factors: The PREDIMED Study. PLoS One. 2013;8(2):1-8. doi:10.1371/journal.pone.0057367
10. Jaceldo-Siegl K, Haddad E, Oda K, Fraser GE, Sabaté J. Tree nuts are inversely associated with metabolic syndrome and obesity: The Adventist Health Study-2. PLoS One. 2014;9(1):1-7. doi:10.1371/journal.pone.0085133
11. Martínez-Lapiscina EH, Pimenta AM, Beunza JJ, Bes-Rastrollo M, Martínez JA, Martínez-González MA. Nut consumption and incidence of hypertension: The SUN prospective cohort. Nutr Metab Cardiovasc Dis. 2010;20(5):359-365. doi:10.1016/j.numecd.2009.04.013
12. Mohammadifard N, Yazdekhasti N, Stangl GI, Sarrafzadegan N. Inverse association between the frequency of nut consumption and obesity among Iranian population: Isfahan Healthy Heart Program. Eur J Nutr. 2015;54(6):925-931. doi:10.1007/s00394-014-0769-x
13. O'Neil CE, Fulgoni VL, Nicklas TA. Tree Nut consumption is associated with better adiposity measures and cardiovascular and metabolic syndrome health risk factors in U.S. Adults: NHANES 2005-2010. Nutr J. 2015;14(1):4-11. doi:10.1186/s12937-015-0052-x

14. Park et al 2016. Daily almond consumption reduces fasting total-, LDL-, and non-HDL cholesterol and body fat mass in healthy young Korean subjects: A randomized controlled trial (abstract-conference paper)

15. Petrović-Oggiano G, Debeljak-Martačić J, Ranković S, et al. The effect of walnut consumption on n-3 fatty acid profile of healthy people living in a non-mediterranean west balkan country, a small scale randomized study. Nutrients. 2020;12(1). doi:10.3390/nu12010192

16. Sabaté J. Nut consumption and body weight. Am J Clin Nutr. 2003;78(3 SUPPL.):647-650. doi:10.1093/ajcn/78.3.647s

17. Teitelbaum J, Zatorre J, Carpenter S, et al. The New England Journal of Medicine Downloaded from nejm.org at UNIVERSITY OF ALBERTA LIBRARY on October 12, 2014. For personal use only. No other uses without permission. Copyright © 1993 Massachusetts Medical Society. All rights reserved. Neurol Sequelae Domoic Acid Intox Due To Ingestion Contam Mussels. 1993;322:2012.

18. Wien et al. The effect of almonds on plasma lipids in persons with Prediabetes. 2008.

Intervention non-related with nut consumption:

1. Agebratt C, Strom E, Romu T, et al. A Randomized Study of the Effects of Additional Fruit and Nuts Consumption on Hepatic Fat Content, Cardiovascular Risk Factors and Basal Metabolic Rate. PLoS One. 2016;11(1):1-13. doi:10.1371/journal.pone.0147149

2. Aronis KN, Vamvini MT, Chamberland JP, et al. Short-term walnut consumption increases circulating total adiponectin and apolipoprotein A concentrations, but does not affect markers of inflammation or vascular injury in obese humans with the metabolic syndrome: Data from a double-blinded, randomized, placebo-controlled study. Metabolism. 2012;61(4):577-582. doi:10.1016/j.metabol.2011.09.008

3. Austel A, Ranke C, Wagner N, Görge J, Ellrott T. Weight loss with a modified Mediterranean-type diet using fat modification: A randomized controlled trial. Eur J Clin Nutr. 2015;69(8):878-884. doi:10.1038/ejcn.2015.11

4. Barbour JA, Howe PRC, Buckley JD, Wright GC, Bryan J, Coates AM. Lower energy intake following consumption of Hi-oleic and regular peanuts compared with iso-energetic consumption of potato crisps. Appetite. 2014;82:124-130. doi:10.1016/j.appet.2014.07.015

5. Bellido C, López-Miranda J, Blanco-Colio LM, et al. Butter and walnuts, but not olive oil, elicit postprandial activation of nuclear transcription factor κB in peripheral blood mononuclear cells from healthy men. Am J Clin Nutr. 2004;80(6):1487-1491. doi:10.1093/ajcn/80.6.1487

6. Butler TJ, Confue C, Guild J, Mushtaq S. Effect of a single serving of pecan nuts on blood lipids and weight: a single blind randomised control trial. Proc Nutr Soc. 2018;77(OCE4):2019. doi:10.1017/s0029665118001702

7. Casas-Agustench P, López-Uriarte P, Bulló M, Ros E, Cabré-Vila JJ, Salas-Salvadó J. Effects of one serving of mixed nuts on serum lipids, insulin resistance and inflammatory markers in patients with the metabolic syndrome. Nutr Metab Cardiovasc Dis. 2011;21(2):126-135. doi:10.1016/j.numecd.2009.08.005

8. Chahibakhsh N, Hosseini E, lslam MS, Rahbar AR. Bitter almond gum reduces body mass index, serum triglyceride, hyperinsulinemia and insulin resistance in overweight subjects with hyperlipidemia. J Funct Foods. 2019;55(February):343-351. doi:10.1016/j.jff.2019.02.040

9. Choudhary et al. Journal A, Diseases I, Publications S. Almond Consumption Decreases Fasting and Post Prandial Blood Glucose Level in Female Type 2 Diabetes Subject Parul , 1 Saroj Kothari and 2 Vimal Sharma Department of Foods and Nutrition , College of Home Science , India Presenting author-Rajasthan College of Agriculture , Maharana Pratap University of Agriculture and Technology , Udaipur 313 001 India. 2009;5(2):109-111.
10. Farr OM, Tuccinardi D, Upadhyay J, Oussaada SM, Mantzoros CS. Walnut consumption increases activation of the insula to highly desirable food cues: A randomized, double-blind, placebo-controlled, cross-over fMRI study. Diabetes, Obes Metab. 2018;20(1):173-177. doi:10.1111/dom.13060
11. Fitschen PJ, Rolfhus KR, Winfrey MR, Allen BK, Manzy M, Maher MA. Cardiovascular effects of consumption of black versus english walnuts. J Med Food. 2011;14(9):890-898. doi:10.1089/jmf.2010.0169
12. Guirette M, Dashti H, Tucker C, Garaulet M, Scheer F, Saxena R. a Novel Food Timing Questionnaire (P18-016-19). Published online 2012:105072. doi:10.1093/cdn/nzz039
13. Hiraoka-Yamamoto J, Ikeda K, Negishi H, et al. Serum lipid effects of a monounsaturated (palmitoleic) fatty acid-rich diet based on macadamia nuts in healthy, young japanese women. Clin Exp Pharmacol Physiol. 2004;31(SUPPL.2):37-38. doi:10.1111/j.1440-1681.2004.04121.x
14. Jenkins DJA, Kendall CWC, Marchie A, et al. The effect of combining plant sterols, soy protein, viscous fibers, and almonds in treating hypercholesterolemia. Metabolism. 2003;52(11):1478-1483. doi:10.1016/S0026-0495(03)00260-9
15. Razquin C, Sanchez-Tainta A, Salas-Salvadó J, et al. Dietary energy density and body weight changes after 3 years in the PREDIMED study. Int J Food Sci Nutr. 2017;68(7):865-872. doi:10.1080/09637486.2017.1295028
16. Reis BZ, Duarte GBS, Vargas-Mendez E, et al. Brazil nut intake increases circulating miR-454-3p and miR-584-5p in obese women. Nutr Res. 2019;67:40-52. doi:10.1016/j.nutres.2019.05.004
17. Tapsell L, Batterham M, Tan SY, Warensjö E. The effect of a calorie controlled diet containing walnuts on substrate oxidation during 8-hours in a room calorimeter. J Am Coll Nutr. 2009;28(5):611-617. doi:10.1080/07315724.2009.10719793
18. Tey SL, Robinson T, Gray AR, Chisholm AW, Brown RC. Do dry roasting, lightly salting nuts affect their cardioprotective properties and acceptability? Eur J Nutr. 2017;56(3):1025-1036. doi:10.1007/s00394-015-1150-4
19. Tuccinardi D, Farr OM, Upadhyay J, et al. Mechanisms underlying the cardiometabolic protective effect of walnut consumption in obese people: A cross-over, randomized, double-blind, controlled inpatient physiology study. Diabetes, Obes Metab. 2019;21(9):2086-2095. doi:10.1111/dom.1377
20. Yang M, Hu FB, Giovannucci EL, et al. Nut consumption and risk of colorectal cancer in women. Eur J Clin Nutr. 2016;70(3):333-337. doi:10.1038/ejcn.2015.66
**Outcome measures non-related with the exposure:**
1. Alper CM, Mattes RD. Peanut consumption improves indices of cardiovascular disease risk in healthy adults. J Am Coll Nutr. 2003;22(2):133-141. doi:10.1080/07315724.2003.10719286
2. Baer DJ, Novotny JA. Consumption of cashew nuts does not influence blood lipids or other markers of cardiovascular disease in humans: A randomized controlled trial. Am J Clin Nutr. 2019;109(2):269-275. doi:10.1093/ajcn/nqy242

3. Bamberger C, Rossmeier A, Lechner K, et al. A walnut-enriched diet reduces lipids in healthy caucasian subjects, independent of recommended macronutrient replacement and time point of consumption: A prospective, randomized, controlled trial. Nutrients. 2017;9(10). doi:10.3390/nu9101097

4. Berryman CE, Grieger JA, West SG, et al. Acute consumption of walnuts and walnut components differentially affect postprandial lipemia, endothelial function, oxidative stress, and cholesterol efflux in humans with mild hypercholesterolemia. J Nutr. 2013;143(6):788-794. doi:10.3945/jn.112.170993

5. Bhardwaj R, Dod H, Sandhu MS, et al. Acute effects of diets rich in almonds and walnuts on endothelial function. Indian Heart J. 2018;70(4):497-501. doi:10.1016/j.ihj.2018.01.030

6. Borkowski K, Yim SJ, Holt RR, et al. Walnuts change lipoprotein composition suppressing TNFα-stimulated cytokine production by diabetic adipocyte. J Nutr Biochem. 2019;68:51-58. doi:10.1016/j.jnutbio.2019.03.004

7. Buijsse B, Boeing H, Drogan D, et al. Consumption of fatty foods and incident type 2 diabetes in populations from eight European countries. Eur J Clin Nutr. 2015;69(4):455-461. doi:10.1038/ejcn.2014.249

8. Canudas S, Hernández-Alonso P, Gali S, et al. Pistachio consumption modulates DNA oxidation and genes related to telomere maintenance: A crossover randomized clinical trial. Am J Clin Nutr. 2019;109(6):1738-1745. doi:10.1093/ajcn/nqz048

9. Cardozo LFMF, Stockler-Pinto MB, Mafra D. Brazil nut consumption modulates Nrf2 expression in hemodialysis patients: A pilot study. Mol Nutr Food Res. 2016;60(7):1719-1724. doi:10.1002/mnfr.201500658

10. Chen CYO, Holbrook M, Duess MA, et al. Effect of almond consumption on vascular function in patients with coronary artery disease: A randomized, controlled, cross-over trial. Nutr J. 2015;14(1):1-11. doi:10.1186/s12937-015-0049-5

11. Chisholm A, Mann J, Skeaff M, et al. A diet rich in walnuts favourably influences plasma fatty acid profile in moderately hyperlipidaemic subjects. Eur J Clin Nutr. 1998;52(1):12-16. doi:10.1038/sj.ejcn.1600507

12. Davis L, Stonehouse W, Loots DT, et al. The effects of high walnut and cashew nut diets on the antioxidant status of subjects with metabolic syndrome. Eur J Nutr. 2007;46(3):155-164. doi:10.1007/s00394-007-0647-x

13. de Souza RGM, Gomes AC, Navarro AM, et al. Baru almonds increase the activity of glutathione peroxidase in overweight and obese women: A randomized, Placebo-controlled trial. Nutrients. 2019;11(8):1-9. doi:10.3390/nu11081750

14. Dhillon J, Li Z, Ortiz RM. Almond Snacking for 8 wk Increases Alpha-Diversity of the Gastrointestinal Microbiome and Decreases Bacteroides fragilis Abundance Compared with an Isocaloric Snack in College Freshmen. Curr Dev Nutr. 2019;3(8):1-9. doi:10.1093/cdn/nzz079

15. Dhillon J, Tan SY, Mattes RD. Effects of almond consumption on the post-lunch dip and long-term cognitive function in energy-restricted overweight and obese adults. Br J Nutr. 2017;117(3):395-402. doi:10.1017/S0007114516004463

16. Di Renzo L, Merra G, Botta R, et al. Post-prandial effects of hazelnut-enriched high fat meal on LDL oxidative status, oxidative and inflammatory gene expression of healthy subjects: a randomized trial. Eur Rev Med Pharmacol Sci. 2017;21(7):1610-1626.

17. Djoussé L, Rudich T, Gaziano JM. Nut consumption and risk of hypertension in US male physicians. Clin Nutr. 2009;28(1):10-14. doi:10.1016/j.clnu.2008.08.005

18. Donadio JLS, Rogero MM, Guerra-Shinohara EM, Desmarchelier C, Borel P, Cozzolino SMF. SEPP1 polymorphisms modulate serum glucose and lipid response to Brazil nut supplementation. Eur J Nutr. 2018;57(5):1873-1882. doi:10.1007/s00394-017-1470-7

19. Edwards K, Kwaw I, Matud J, Kurtz I. Effect of Pistachio Nuts on Serum Lipid Levels in Patients with Moderate Hypercholesterolemia. J Am Coll Nutr. 1999;18(3):229-232. doi:10.1080/07315724.1999.10718856

20. Eneroth H, Wallin S, Leander K, Sommar JN, Åkesson A. Risks and benefits of increased nut consumption: Cardiovascular health benefits outweigh the burden of carcinogenic effects attributed to aflatoxin B1 exposure. Nutrients. 2017;9(12). doi:10.3390/nu9121355

21. Fadelu T, Zhang S, Niedzwiecki D, et al. Nut consumption and survival in patients with stage III colon cancer: Results from CALGB 89803 (Alliance). J Clin Oncol. 2018;36(11):1112-1120. doi:10.1200/JCO.2017.75.5413

22. Griel AE, Cao Y, Bagshaw DD, Cifelli AM, Holub B, Kris-Etherton PM. A Macadamia nut-rich diet reduces total and LDL-cholesterol in mildly hypercholesterolemic men and women. J Nutr. 2008;138(4):761-767. doi:10.1093/jn/138.4.761

23. Haddad E, Jambazian P, Karunia M, Tanzman J, Sabaté J. A pecan-enriched diet increases γ-tocopherol/cholesterol and decreases thiobarbituric acid reactive substances in plasma of adults. Nutr Res. 2006;26(8):397-402. doi:10.1016/j.nutres.2006.06.022

24. Hernández-Alonso P, Giardina S, Salas-Salvadó J, Arcelin P, Bulló M. Chronic pistachio intake modulates circulating microRNAs related to glucose metabolism and insulin resistance in prediabetic subjects. Eur J Nutr. 2017;56(6):2181-2191. doi:10.1007/s00394-016-1262-5

25. Holligan SD, West SG, Gebauer SK, Kay CD, Kris-Etherton PM. A moderate-fat diet containing pistachios improves emerging markers of cardiometabolic syndrome in healthy adults with elevated LDL levels. Br J Nutr. 2014;112(5):744-752. doi:10.1017/S0007114514001561

26. Holscher HD, Guetterman HM, Swanson KS, et al. Walnut consumption alters the gastrointestinal microbiota, microbially derived secondary bile acids, and health markers in healthy adults: A randomized controlled trial. J Nutr. 2018;148(6):861-867. doi:10.1093/jn/nxy004

27. Huetos AS, Muralidharan J, Galiè S, Salas-Salvadó J, Bulló M. Effect of nut consumption on erectile and sexual function in healthy males: A Secondary outcome analysis of the fertinuts randomized controlled trial. Nutrients. 2019;11(6). doi:10.3390/nu11061372

28. Iwamoto M, Sato M, Kono M, et al. Retracted: Walnuts Lower Serum Cholesterol in Japanese Men and Women. J Nutr. 2000;130(2):171-176. doi:10.1093/jn/130.2.171

29. Iyer SS, Boateng LA, Sales RL, et al. Effects of peanut oil consumption on appetite and food choice. Int J Obes. 2006;30(4):704-710. doi:10.1038/sj.ijo.0803180

30. Jaceldo-Siegl K, Sabaté J, Rajaram S, Fraser GE. Long-term almond supplementation without advice on food replacement induces favourable nutrient modifications to the habitual diets of free-living individuals. Br J Nutr. 2004;92(3):533-540. doi:10.1079/bjn20041223

31. Kay CD, Gebauer SK, West SG, Kris-Etherton PM. Pistachios increase serum antioxidants and lower serum oxidized-LDL in hypercholesterolemic adults. J Nutr. 2010;140(6):1093-1098. doi:10.3945/jn.109.117366

32. Kurlandsky SB, Stote KS. Cardioprotective effects of chocolate and almond consumption in healthy women. Nutr Res. 2006;26(10):509-516. doi:10.1016/j.nutres.2006.08.007

33. Lovejoy JC, Most MM, Lefevre M, Greenway FL, Rood JC. Effect of diets enriched in almonds on insulin action and serum lipids in adults with normal glucose tolerance or type 2 diabetes. Am J Clin Nutr. 2002;76(5):1000-1006. doi:10.1093/ajcn/76.5.1000
34. McKay DL, Eliasziw M, Oliver Chen CY, Blumberg JB. A pecan-rich diet improves cardiometabolic risk factors in overweight and obese adults: A randomized controlled trial. Nutrients. 2018;10(3). doi:10.3390/nu10030339
35. Morgan JM, Horton K, Reese D, Carey C, Walker K, Capuzzi DM. Effects of walnut consumption as part of a low-fat, low-cholesterol diet on serum cardiovascular risk factors. Int J Vitam Nutr Res. 2002;72(5):341-347. doi:10.1024/0300-9831.72.5.341
36. Nieuwenhuis L, van den Brandt PA. Nut and peanut butter intake are not directly associated with the risk of endometrial or ovarian cancer: Results from a Dutch prospective cohort study. Clin Nutr. 2020;39(7):2202-2210. doi:10.1016/j.clnu.2019.09.008
37. Nishi S, Kendall CWC, Gascoyne AM, et al. Effect of almond consumption on the serum fatty acid profile: A dose-response study. Br J Nutr. 2014;112(7):1137-1146. doi:10.1017/S0007114514001640
38. Rajaram S, Burke K, Connell B, Myint T, Sabaté J. A monounsaturated fatty acid-rich pecan-enriched diet favorably alters the serum lipid profile of healthy men and women. J Nutr. 2001;131(9):2275-2279. doi:10.1093/jn/131.9.2275
39. Sala-Vila A, Valls-Pedret C, Rajaram S, et al. Effect of a 2-year diet intervention with walnuts on cognitive decline. The Walnuts and Healthy Aging (WAHA) study: A randomized controlled trial. Am J Clin Nutr. 2020;111(3):590-600. doi:10.1093/ajcn/nqz328
40. Salas-Huetos A, Moraleda R, Giardina S, et al. Effect of nut consumption on semen quality and functionality in healthy men consuming a Western-style diet: A randomized controlled trial. Am J Clin Nutr. 2018;108(5):953-962. doi:10.1093/ajcn/nqy181
41. Sayer RD, Dhillon J, Tamer GG, et al. Consuming almonds vs. Isoenergetic baked food does not differentially influence postprandial appetite or neural reward responses to visual food stimuli. Nutrients. 2017;9(8). doi:10.3390/nu9080807
42. Tey SL, Brown RC, Gray AR, Chisholm AW, Delahunty CM. Long-term consumption of high energy-dense snack foods on sensory-specific satiety and intake. Am J Clin Nutr. 2012;95(5):1038-1047. doi:10.3945/ajcn.111.030882
43. Tong TYN, Appleby PN, Key TJ, et al. The associations of major foods and fibre with risks of ischaemic and haemorrhagic stroke: a prospective study of 418 329 participants in the EPIC cohort across nine European countries. Eur Heart J. 2020;41(28):2632-2640. doi:10.1093/eurheartj/ehaa007
44. Torabian S, Haddad E, Cordero-Macintyre Z, Tanzman J, Fernandez ML, Sabate J. Long-term walnut supplementation without dietary advice induces favorable serum lipid changes in free-living individuals. Eur J Clin Nutr. 2010;64(3):274-279. doi:10.1038/ejcn.2009.152
45. Wu L, Piotrowski K, Rau T, et al. Walnut-enriched diet reduces fasting non-hdl-cholesterol and apolipoprotein b in healthy caucasian subjects: A randomized controlled cross-over clinical trial. Metabolism. 2014;63(3):382-391. doi:10.1016/j.metabol.2013.11.005
46. Almond consumption affects fecal microbiota composition, stool pH, and stool moisture in overweight and obese adults with elevated fasting blood glucose: A randomized controlled trial
47. Acute effects of delayed-release hydrolyzed pine nut oil on glucose tolerance, incretins, ghrelin and appetite in healthy humans

48. Sperm DNA methylation changes after short-term nut supplementation in healthy men consuming a Western-style diet
49. Adherence to the Mediterranean Lifestyle and Desired Body Weight Loss in a Mediterranean Adult Population with Overweight: A PREDIMED-Plus Study.
**Study design:**
1. Li M, Shi Z. A Prospective Association of Nut Consumption with Cognitive Function in Chinese Adults Aged 55+ _ China Health and Nutrition Survey. J Nutr Heal Aging. 2019;23(2):211-216. doi:10.1007/s12603-018-1122-5
2. Abbey M, Noakes M, Belling GB, Nestel PJ. Partial replacement of saturated fatty acids with almonds or walnuts lowers total plasma cholesterol and low-density-lipoprotein cholesterol. Am J Clin Nutr. 1994;59(5):995-999. doi:10.1093/ajcn/59.5.995
3. Bitok et al, 2016.Does a Daily Walnut Supplement Given for a Year Result in Body Weight Gain?
4. Celis-Morales C, Livingstone KM, Affleck A, et al. Correlates of overall and central obesity in adults from seven European countries: Findings from the Food4Me Study. Eur J Clin Nutr. 2018;72(2):207-219. doi:10.1038/s41430-017-0004-y
5. Cominetti C, de Bortoli MC, Purgatto E, et al. Associations between glutathione peroxidase-1 Pro198Leu polymorphism, selenium status, and DNA damage levels in obese women after consumption of Brazil nuts. Nutrition. 2011;27(9):891-896. doi:10.1016/j.nut.2010.09.003
6. Garg ML, Blake RJ, Wills RBH, Clayton EH. Macadamia nut consumption modulates favourably risk factors for coronary artery disease in hypercholesterolemic subjects. Lipids. 2007;42(6):583-587. doi:10.1007/s11745-007-3042-8
7. Garg ML, Blake RJ, Wills RBH. Human Nutrition and Metabolism Lowers Plasma Total and LDL. J Nutr. 2003;(March):1060-1063.
8. Gillen LJ, Tapsell LC, Patch CS, Owen A, Batterham M. Structured dietary advice incorporating walnuts achieves optimal fat and energy balance in patients with type 2 diabetes mellitus. J Am Diet Assoc. 2005;105(7):1087-1096. doi:10.1016/j.jada.2005.04.007
9. Gulati S, Misra A, Pandey RM. Effect of Almond Supplementation on Glycemia and Cardiovascular Risk Factors in Asian Indians in North India with Type 2 Diabetes Mellitus: A 24-Week Study. Metab Syndr Relat Disord. 2017;15(2):98-105. doi:10.1089/met.2016.0066
10. Hershey M, Mateos LG, Ruiz-Estigarribia L, et al. Effective Dietary Behavior Change Using an Online Nutrition Intervention with a Mediterranean Diet Plus Extra-virgin Olive Oil for the Prevention of Recurrent Arrhythmia (P12-006-19). Curr Dev Nutr. 2019;3(Supplement_1):1156-1158. doi:10.1093/cdn/nzz035.p12-006-19
11. Sari I, Baltaci Y, Bagci C, et al. Effect of pistachio diet on lipid parameters, endothelial function, inflammation, and oxidative status: A prospective study. Nutrition. 2010;26(4):399-404. doi:10.1016/j.nut.2009.05.023
12. Zaveri S, Drummond S. The effect of including a conventional snack (cereal bar) and a nonconventional snack (almonds) on hunger, eating frequency, dietary intake and body weight. J Hum Nutr Diet. 2009;22(5):461-468. doi:10.1111/j.1365-277X.2009.00983.x

13. Asghari G, Ghorbani Z, Mirmiran P, Azizi F. Nut consumption is associated with lower incidence of type 2 diabetes: The Tehran Lipid and Glucose Study. Diabetes Metab. 2017;43(1):18-24. doi:10.1016/j.diabet.2016.09.008
14. Bao Y, Hu FB, Giovannucci EL, et al. Nut consumption and risk of pancreatic cancer in women. Br J Cancer. 2013;109(11):2911-2916. doi:10.1038/bjc.2013.665
15. Bes-Rastrollo M, Sabaté J, Gómez-Gracia E, Alonso A, Martinez JA, Martínez-González MA. Nut consumption and weight gain in a mediterranean cohort: The SUN study. Obesity. 2007;15(1):107. doi:10.1038/oby.2007.507
16. De La Fuente-Arrillaga C, Zazpe I, Santiago S, et al. Beneficial changes in food consumption and nutrient intake after 10 years of follow-up in a Mediterranean cohort: The SUN project. BMC Public Health. 2016;16(1):1-11. doi:10.1186/s12889-016-2739-0
17. Di Giuseppe R, Fjeld MK, Dierkes J, et al. The association between nut consumption and the risk of total and ischemic stroke in a German cohort study. Eur J Clin Nutr. 2015;69(4):431-435. doi:10.1038/ejcn.2014.212
18. Freisling H, Noh H, Slimani N, et al. Nut intake and 5-year changes in body weight and obesity risk in adults: results from the EPIC-PANACEA study. Eur J Nutr. 2018;57(7):2399-2408. doi:10.1007/s00394-017-1513-0
19. Kochar J, Gaziano JM, Djoussé L. Nut consumption and risk of type II diabetes in the Physicians Health Study. Eur J Clin Nutr. 2010;64(1):75-79. doi:10.1038/ejcn.2009.121
20. Konieczna J, Romaguera D, Pereira V, et al. Longitudinal association of changes in diet with changes in body weight and waist circumference in subjects at high cardiovascular risk: The PREDIMED trial. Int J Behav Nutr Phys Act. 2019;16(1):1-13. doi:10.1186/s12966-019-0893-3
21. Liu G, Guasch-Ferré M, Hu Y, et al. Nut Consumption in Relation to Cardiovascular Disease Incidence and Mortality among Patients with Diabetes Mellitus. Circ Res. 2019;124(6):920-929. doi:10.1161/CIRCRESAHA.118.314316
22. Mohammadifard N, Haghighatdoost F, Mansourian M, et al. Long-term association of nut consumption and cardiometabolic risk factors. Nutr Metab Cardiovasc Dis. 2019;29(9):972-982. doi:10.1016/j.numecd.2019.04.014
23. Mozaffarian D, Hao T, Rimm EB, Willett WC, Hu FB. Changes in diet and lifestyle and long-term weight gain in women and men. N Engl J Med. 2011;364(25):2392-2404. doi:10.1056/NEJMoa1014296
24. Sterling SR, Bertrand B, Judd S, Carson TL, Chandler-Laney P, Baskin ML. Longitudinal Analysis of Nut-Inclusive Diets and Body Mass Index Among Overweight and Obese African American Women Living in Rural Alabama and Mississippi, 2011-2013. Prev Chronic Dis. 2017;14:E82. doi:10.5888/pcd14.160595
**Same population as included studies:**
1. Abazarfard Z, Eslamian G, Salehi M, Keshavarzi S. A randomized controlled trial of the effects of an almond-enriched, hypocaloric diet on liver function tests in overweight/obese women. Iran Red Crescent Med J. 2016;18(3). doi:10.5812/ircmj.23628
2. Alves RDM, Moreira APB, Macedo VS, Costa NMB, Alfenas R de CG, Bressan J. Cacahuete alto-oleico aumenta la termogénesis inducida por la dieta en hombres con sobrepeso y obesidad. Nutr Hosp. 2014;29(5):1024-1032. doi:10.3305/nh.2014.29.5.7235

3. Barbour JA, Howe PRC, Buckley JD, Bryan J, Coates AM. Cerebrovascular and cognitive benefits of high-oleic peanut consumption in healthy overweight middle-aged adults. Nutr Neurosci. 2017;20(10):555-562. doi:10.1080/1028415X.2016.1204744

4. Berryman CE, Fleming JA, Kris-Etherton PM. Inclusion of almonds in a cholesterol-lowering diet improves plasma HDL subspecies and cholesterol efflux to serum in normal-weight individuals with elevated LDL cholesterol. J Nutr. 2017;147(8):1517-1523. doi:10.3945/jn.116.245126

5. Canales A, Bastida S, Librelottto J, Nus M, Sánchez-Muniz FJ, Benedi J. Platelet aggregation, eicosanoid production and thrombogenic ratio in individuals at high cardiovascular risk consuming meat enriched in walnut paste. A crossover, placebo-controlled study. Br J Nutr. 2009;102(1):134-141. doi:10.1017/S000711450813361X

6. Dhillon J, Ortiz R. Incorporation of a Morning Snack (Almonds vs. Crackers) Differentially Altered Serum Primary Metabolite Profiles of Breakfast Skipping Young Adults (FS03-03-19). Curr Dev Nutr. 2019;3(Supplement_1):663. doi:10.1093/cdn/nzz046.fs03-03-19

7. Hernández-Alonso P, Salas-Salvadó J, Baldrich-Mora M, Mallol R, Correig X, Bulló M. Effect of pistachio consumption on plasma lipoprotein subclasses in pre-diabetic subjects. Nutr Metab Cardiovasc Dis. 2015;25(4):396-402. doi:10.1016/j.numecd.2015.01.013

8. Jenkins DJA, Kendall CWC, Marchie A, et al. Almonds reduce biomarkers of lipid peroxidation in older hyperlipidemic subjects. J Nutr. 2008;138(5):908-913. doi:10.1093/jn/138.5.908

9. Kranz S, Hill AM, Fleming JA, Hartman TJ, West SG, Kris-Etherton PM. Nutrient displacement associated with walnut supplementation in men. J Hum Nutr Diet. 2014;27(SUPPL2):247-254. doi:10.1111/jhn.12146

10. Liu JF, Liu YH, Chen CM, Chang WH, Chen CYO. The effect of almonds on inflammation and oxidative stress in Chinese patients with type 2 diabetes mellitus: A randomized crossover controlled feeding trial. Eur J Nutr. 2013;52(3):927-935. doi:10.1007/s00394-012-0400-y

11. López-Uriarte Patricia P, Nogués R, Saez G, et al. Effect of nut consumption on oxidative stress and the endothelial function in metabolic syndrome. Clin Nutr. 2010;29(3):373-380. doi:10.1016/j.clnu.2009.12.008

12. Mora-Cubillos X, Tulipani S, Garcia-Aloy M, Bulló M, Tinahones FJ, Andres-Lacueva C. Plasma metabolomic biomarkers of mixed nuts exposure inversely correlate with severity of metabolic syndrome. Mol Nutr Food Res. 2015;59(12):2480-2490. doi:10.1002/mnfr.201500549

13. Muñoz S, Merlos M, Zambón D, et al. Walnut-enriched diet increases the association of LDL from hypercholesterolemic men with human HepG2 cells. J Lipid Res. 2001;42(12):2069-2076.

14. Nishi SK, Kendall CWC, Bazinet RP, et al. Nut consumption, serum fatty acid profile and estimated coronary heart disease risk in type 2 diabetes. Nutr Metab Cardiovasc Dis. 2014;24(8):845-852. doi:10.1016/j.numecd.2014.04.001

15. Pieters M, Oosthuizen W, Jerling JC, Loots DT, Mukuddem-Petersen J, Hanekom SM. Clustering of haemostatic variables and the effect of high cashew and walnut diets on these variables in metabolic syndrome patients. Blood Coagul Fibrinolysis. 2005;16(6):429-437. doi:10.1097/01.mbc.0000174966.86549.27

16. Rajaram S, Connell KM, Sabaté J. Effect of almond-enriched high-monounsaturated fat diet on selected markers of inflammation: A randomised, controlled, crossover study. Br J Nutr. 2010;103(6):907-912. doi:10.1017/S0007114509992480

17. Alper CM, Mattes RD. Effects of chronic peanut consumption on energy balance and hedonics. Int J Obes. 2002;26(8):1129-1137. doi:10.1038/sj.ijo.0802050

18. Babio N, Toledo E, Estruch R, et al. Mediterranean diets and metabolic syndrome status in the PREDIMED randomized trial. Cmaj. 2014;186(17):E649-E657. doi:10.1503/cmaj.140764

19. Bowen J, Luscombe-Marsh ND, Stonehouse W, et al. Effects of almond consumption on metabolic function and liver fat in overweight and obese adults with elevated fasting blood glucose: A randomised controlled trial. Clin Nutr ESPEN. 2019;30:10-18. doi:10.1016/j.clnesp.2018.12.088

20. Fraser GE, Bennett HW, Jaceldo KB, Sabaté J. Effect on Body Weight of a Free 76 Kilojoule (320 Calorie) Daily Supplement of Almonds for Six Months. J Am Coll Nutr. 2002;21(3):275-283. doi:10.1080/07315724.2002.10719221

21. Hou YY, Ojo O, Wang LL, et al. A randomized controlled trial to compare the effect of peanuts and almonds on the cardio-metabolic and inflammatory parameters in patients with type 2 diabetes mellitus. Nutrients. 2018;10(11):1-16. doi:10.3390/nu10111565

22. Huguenin GVB, Oliveira GMM, Moreira ASB, et al. Improvement of antioxidant status after Brazil nut intake in hypertensive and dyslipidemic subjects. Nutr J. 2015;14(1). doi:10.1186/s12937-015-0043-y

23. Jaceldo-Siegl K, Sabaté J, Batech M, Fraser GE. Influence of body mass index and serum lipids on the cholesterol-lowering effects of almonds in free-living individuals. Nutr Metab Cardiovasc Dis. 2011;21(SUPPL. 1). doi:10.1016/j.numecd.2011.03.007

24. Lima RPA, do Nascimento RAF, Luna RCP, et al. Effect of a diet containing folate and hazelnut oil capsule on the methylation level of the ADRB3 gene, lipid profile and oxidative stress in overweight or obese women. Clin Epigenetics. 2017;9(1):1-9. doi:10.1186/s13148-017-0407-6

25. Mah E, Schulz JA, Kaden VN, et al. Cashew consumption reduces total and LDL cholesterol: A randomized, crossover, controlled-feeding trial. Am J Clin Nutr. 2017;105(5):1070-1078. doi:10.3945/ajcn.116.150037

26. Ndanuko RN, Tapsell LC, Charlton KE, Neale EP, Batterham MJ. Effect of individualised dietary advice for weight loss supplemented with walnuts on blood pressure: The HealthTrack study. Eur J Clin Nutr. 2018;72(6):894-903. doi:10.1038/s41430-018-0123-0

27. Neale EP, Tapsell LC, Martin A, Batterham MJ, Wibisono C, Probst YC. Impact of providing walnut samples in a lifestyle intervention for weight loss: A secondary analysis of the HealthTrack trial. Food Nutr Res. 2017;61(1). doi:10.1080/16546628.2017.1344522

28. Tapsell LC, Lonergan M, Batterham MJ, et al. Effect of interdisciplinary care on weight loss: A randomised controlled trial. BMJ Open. 2017;7(7):1-12. doi:10.1136/bmjopen-2016-014533

29. Wang X, Li Z, Liu Y, Lv X, Yang W. Effects of pistachios on body weight in Chinese subjects with metabolic syndrome. Nutr J. 2012;11(1):1-6. doi:10.1186/1475-2891-11-20

Table S3. Characteristics of the included studies.

Author, Year	Design	Country	Study population	n	% women	Age (mean/sd; range)	Baseline BW	Baseline BMI	Type of nut	Nut consumption (g/d; kcal/d; %energy)	Control	Duration (wks)	Outcome	Main results
Abazarfard, 2014	RCT parallel	Iran	OW/obese	IG: 50<br>CG: 50	100%	IG: 42.4±7.3<br>CG: 42.9±6.8	IG: 76.4±2.7<br>CG: 75.6±2.4	IG: 29.9±1.2<br>CG: 29.4±1.7	Almonds	50g/d (snack)	Balanced hypocaloric diet	12	BW (kg)<br>BMI (kg/m2)<br>WC (cm)<br>WHR	BW, BMI, WC and WHR decreased significantly in the almond group compared with nut-free group (p<0.001)
Abbaspour, 2019	RCT parallel	USA	OW/obese	IG: 24<br>CG: 24	39.6%	IG: 30.4±10.2<br>CG: 29.1±9.3	IG: 90.3±13.8<br>CG: 95.1±12.2	IG: 30.7±2.8<br>CG: 31.6±3.1	Mixed nuts	42.5g/d	Isocaloric snack (unsalted pretzels)	8	BW (kg)<br>BMI (kg/m2)<br>WC (cm)<br>WHR<br>BF%	Significant reduction on BW and BMI in the nut group compared to control group
Abdrabalnabi, 2020	RCT parallel	USA and Spain	Healthy	Total: 625<br>IG: 324<br>CG: 312	67%	69.1±3.6	73.5±15.2	-	Walnuts	30-60g/d	Habitual diet	96	BMI (kg/m2)	Not significant group x time interaction
Álvarez-Pérez, 2016	RCT parallel	Spain	CVD or at CV risk	IG: 111<br>CG: 87	66%	55-80	IG: 80.2±11.8<br>CG: 79.3±12.2	IG: 31.1±3.9<br>CG: 31.3±3.9	Mixed nuts	30g/d: 15g walnuts, 7.5g almonds and 7.5g hazelnuts	Low-fat diet	48	BW (kg)<br>BMI (kg/m2)<br>WC (cm)<br>BF%<br>Total fat mass (kg)	Significant reduction in WC for nuts group, control group showed a significant increase in BF%. No differences between groups
Alves, 2014	RCT parallel	Brazil	OW/obese	IG: 21<br>IG2: 22<br>CG: 22	100%	IG1: 27±2<br>IG2: 28±1.5<br>IG3: 27.4±1.6	IG1: 95.1±2.4<br>IG2: 93.4±2.2<br>CG: 94.5±2.5	IG1: 30±0.6<br>IG2: 29.5±0.4<br>CG: 29.7±0.6	Peanuts	56g/d (conventional and high-oleic peanuts)	Nut-free hypocaloric diet	4	BW (kg)<br>BMI (kg/m2)<br>WC (cm)<br>Hip (cm)<br>WHR<br>BF%<br>BFM (kg)	Significant decrease in BF% in high oleic peanut group
Barbour, 2015	RCT crossover	Australia	OW/obese	IG: 61	52.5%	65±7	87.7±14	30.6±4	Peanuts (roasted, unsalted)	56-84g/d; 15-20% energy intake	Habitual diet	12	BW (kg)<br>BMI (kg/m2)<br>WC (cm)	No differences in body composition, and less than predicted increase (0.5 kg) in body BW

Author, Year	Design	Country	Study population	n	% women	Age (mean/sd; range)	Baseline BW	Baseline BMI	Type of nut	Nut consumption (g/d; kcal/d; %energy)	Control	Duration (wks)	Outcome	Main results
Bashan, 2018	RCT parallel	Turkey	Healthy	IG: 73<br>CG: 72	66.3%	IG: 41.0±10.8<br>CG: 40.7±9.6	IG: 86.3±11.2<br>CG: 85.0±13.2	IG: 30.9±2.6<br>CG: 30.1±2.9	Walnuts	40-50g/d (Maras 18)	Individualized diet recommended by AHA dietary guidelines	12	BW (kg)	Not significant differences between groups (p=0.42)
Bento, 2014	RCT crossover	Brazil	Hypercholesterolemia/hyperlipidemic	IG: 20	60%	35±2.7	IG: 64±2.4<br>CG: 63.7±2.6	IG: 23.2±0.5<br>CG: 23.1±0.5	Almonds (baru)	20g/d	Placebo (corn starch capsule)	6	BW (kg)<br>BMI (kg/m2)<br>BF%	Not significant reduction
Berryman, 2015	RCT crossover	USA	Hypercholesterolemia/hyperlipidemic	IG: 48	54%	50±9.4	74.7±1.5	26.2±2.8	Almonds	1.5oz/d	Same diet plus isocaloric muffin	6	BW (kg)<br>FM (kg)<br>WC (cm)<br>Abdominal FM (kg)	Almond consumption reduced abdominal fat significantly (p=0.02)
Bitok, 2018	RCT parallel	USA	Healthy	IG: 159<br>CG: 148	67%	IG: 69.7±4.1<br>CG: 69.1±3.7	IG: 77.1±17.2<br>CG: 75.6±16.1	IG: 27.5±4.8<br>CG: 27.4±4.8	Walnuts	56g/d (15% energy requirements)	Habitual diet	96	BW (kg)<br>WC (cm)<br>WHR<br>BF%	No significant differences were observed between groups for BW, body fat, WC or WHR
Bowen, 2019	RCT parallel	Australia	T2DM	IG: 39<br>CG: 37	40.8%	IG: 60.8±6.6<br>CG:60.6±8.8	IG: 102±18.5<br>CG: 95.8±18.4	IG: 34.4±6.2<br>CG: 33.2±4.9	Almonds (raw)	56g/d (two servings as morning and afternoon snacks)	Isocaloric snack (biscuit)	8	BW (kg)<br>WC (cm)<br>FM (kg)<br>VAT (cm)	No significant changes between groups
Bulló, 2009	RCT parallel	Spain	CVD or at CV risk	IG1: 70<br>IG2: 73<br>CG: 59	48.5%	IG1: 68.4±6<br>IG2: 67.8±6.5<br>CG: 67.8±6.1	-	IG1: 29.4±3.3<br>IG2: 29.2±3<br>CG: 29.6±3.2	Mixed nuts	Walnuts 15g/d; hazelnuts 7.5g/d; almonds 7.5g/d	Low-fat diet	48	BMI (kg/m2)<br>WC (cm)	Not significant changes
Caldas, 2020	RCT parallel	Brazil	OW/obese	IG1: 21<br>IG2: 21<br>CG: 22	0%	IG1:28±1.5<br>IG2:27.2±1.8<br>CG:27.4±1.6	IG1: 92.7±2.1<br>IG2: 95.5±2.2<br>CG: 94.5±2.4	IG1: 29.4±0.4<br>IG2: 30.1±0.5<br>CG: 29.7±0.5	Peanuts (conventional vs high oleic)	IG1: Conventional peanuts; 56g/d<br>IG2: High-oleic peanut; 56g/d<br>Hypocaloric diet (-250kcal)	Hypocaloric diet (-250kcal)	4	BW (kg)<br>BMI (kg/m2)<br>WC (cm)	Not significant differences between groups, but significant reduction of BW, BMI and WC intragroup
Canales, 2007	RCT crossover	Spain	OW/obese	IG: 22	40%	54.8±8.3	81.0±12.9	29.6±3.4	Walnuts	150g/wk, 21.4g/d	Habitual diet plus meat	5	BMI (kg/m2)	Not significant change (p=0.05)

Author, Year	Design	Country	Study population	n	% women	Age (mean/sd; range)	Baseline BW	Baseline BMI	Type of nut	Nut consumption (g/d; kcal/d; %energy)	Control	Duration (wks)	Outcome	Main results
Carughi, 2019	RCT parallel	France	Healthy	IG: 30<br>CG: 30	100%	23-49 (35±7)	IG: 58.5±6<br>CG: 57.3±5.8	-	Pistachios	56g/d	Isocaloric and isoprotein biscuit	4	BW (kg)<br>WC (cm)<br>WHR<br>BF%	Not significant changes
Casas-Agustench, 2011	RCT parallel	Spain	MetS	IG: 25<br>CG: 25	44%	IG: 52.9±8.4<br>CG: 50.6±8.4	(95%CI)<br>IG: 86.4 (80.8-92)<br>CG: 79.9 (75.5-84.4)	95%CI)<br>IG: 31.6 (30.5-32.8)<br>CG: 30 (28.6-31.4)	Mixed nuts	Walnuts 15g/d; hazelnuts 7.5g/d; almonds 7.5g/d	AHA dietary guidelines	12	BW (kg)<br>WC (cm)<br>BF%	Significant differences from baseline, not significant differences between groups
Chen, 2017	RCT crossover	Taiwan	T2DM	IG:33	60.6%	55±10.5	IG:65.3±11<br>CG: 64.7±10.8	IG: 25.6±4.3<br>CG: 25.3±4.1	Almonds (roasted)	60g/d	NCEP Step II	12	BW (kg)<br>BMI (kg/m2)<br>WC (cm)<br>WHR<br>BF%	Not significant differences between groups
Chiang, 2012	RCT crossover	USA	Hypercholesterolemia/hyperlipidemic	IG: 25	44%	IG: 23-65	70.9 (51.5-115.8)	24.8 (18.7-36-6)	Walnuts	36.42g/d (42.5g/ 6times per week)	Fish diet (113g fatty fish, 2 times/wk) and control diet without nuts or fish	4	BW (kg)	Not significant changes in mean body BW between groups
Chisholm, 2005	RCT crossover	New Zealand	Healthy	IG: 28	-	48	IG: 74.2±11.6;<br>CG: 74.1±11.7	IG: 26.8±3.2;<br>CG: 26.8±3.4	Mixed nuts	30g/d	Habitual diet plus cereal	6	BW (kg); BMI (kg/m2); WHR	No significant differences between the values at the end of the intervention periods for BW, BMI, waist to hip ratio
Claesson, 2009	RCT parallel	Sweden	Healthy	IG1: 13<br>CG: 12	53.8%	IG1: 68.4±6<br>CG: 67.8±6.1	IG: 68.7±6.1<br>CG: 67.3±47.6	IG: 22.2±2<br>CG: 22.2±1.4	Peanuts (roasted and salted)	20kcal/kg/d	Isocaloric candy (20kcal/kg/d)	2	BW (kg)<br>BMI (kg/m2)<br>WC (cm)<br>BF%	Not significant changes in the nut group<br><br>WC, BMI increased in the control group
Coates, 2020	RCT parallel	Australia	OW/obese	IG: 63<br>CG: 65	60.9%	IG: 64±8<br>CG: 65±8	IG: 84.4±12<br>CG: 85.4±14	IG: 30.3±3.6<br>CG: 30.5±3.8	Almonds (whole, raw)	15% energy intake	Isocaloric nut-free diet	12	BW (kg)<br>BMI (kg/m2)<br>WC (cm)<br>BF%	Not significant changes

Author, Year	Design	Country	Study population	n	% women	Age (mean/sd; range)	Baseline BW	Baseline BMI	Type of nut	Nut consumption (g/d; kcal/d; %energy)	Control	Duration (wks)	Outcome	Main results
Costa e Silva, 2019	RCT parallel	Brazil	MetS	IG: 15 CG:16	-	36-65	-	IG: 32.2±4.4 CG: 30.8±4.4	Brazil nut (virgin extra oil)	10mL/d	Supply of soybean oil (10mL/d)	4	BMI (kg/m2) WC (cm) BF%	Not significant changes
Damasceno, 2011	RCT crossover	Spain	Hypercholesterolemia/hyperlipidemic	IG: 18	50%	56±13	70.7±18.6	25.7±2.3	Almonds (raw) vs Walnuts	Almonds: 50-75g/d; Walnuts: 40-65g/d	Isoenergetic Mediterranean diet plus Virgin Olive Oil	4	BW (kg)	Not significant differences between groups (p=0.60)
Damasceno, 2013	RCT parallel	Spain	Hypercholesterolemia/hyperlipidemic	IG: 58 CG: 52	55%	55-88y	IG: 75.4 (72.5; 78.4) CG: 77.6 (74.4; 80.7)	IG: 29 (28.1;29.9) CG: 29.7 (28.7; 30.7)	Mixed nuts	30g/d: 15g walnuts, 7.5g almonds and 7.5g hazelnuts	Low-fat diet	48	BW (kg) BMI (kg/m2) WC (cm)	MeDiet + nuts participants showed significant reductions from baseline in WC
Damavandi, 2013	RCT parallel	Iran	T2DM	IG: 23 CG:25	68%	IG: 55.7±7.7	IG: 72.1±10.3 CG: 72±9.6	IG: 28.47±3.57 CG: 28.2±3.55	Hazelnuts (raw, unsalted)	29g/d as snacks (10% energy intake)	Habitual diet	8	BW (kg) BMI (kg/m2)	Not significant changes in BW nor BMI throughout the study
Damavandi, 2019	RCT parallel	Iran	T2DM	IG: 22 CG: 21	79%	53.86 ± 7.2	IG: 72.1±13.2 CG: 71.9±9.8	IG: 28.7±5.8 CG: 28.6±3.1	Cashews	28g/d (10% energy intake)	Habitual diet	8	BW (kg) BMI (kg/m2) WC (cm)	Not significant differences intra or intergroups comparisons
Dhillon, 2016	RCT parallel	USA	OW/obese	IG: 36 CG: 37	69.7%	IG: 33.6±13 CG: 35±13	IG: 83.5±13 CG: 82.2±14.6	IG: 30.3±3.2 CG: 30.6±3.9	Almond (dry-roasted, lightly salted)	15% energy intake	Energy-restricted diet (500kcal), same as IG	12	BW (kg) VAT (cm) WC (cm)	Significant reduction on BW, VAT and WC
Dhillon, 2018	RCT parallel	USA	Healthy	IG: 38 CG: 35	56.1%	18-19y	IG: 71.5±18.6 CG: 71.3±15	IG: 25.6±5 CG: 25.3±4.5	Almonds (dry roasted)	56.7g/d (364kcal)	Graham cracker snack (338kcal)	8	BW (kg) WC (cm) FM (kg) BF%	Similar body mass gains over 8 weeks for both groups
Dikariyanto, 2020	RCT parallel	UK	CVD or at CV risk	IG: 54 CG: 51		IG: 56.3±10.3 CG: 56±10.7	-	IG: 27.3±4.4 CG: 26.7±4.5	Almonds (whole, roasted)	20%energy intake (as snacks)	Control snacks	6	BMI (kg/m2) WC (cm) BF%	No differences in body composition

Author, Year	Design	Country	Study population	n	% women	Age (mean/sd; range)	Baseline BW	Baseline BMI	Type of nut	Nut consumption (g/d; kcal/d; %energy)	Control	Duration (wks)	Outcome	Main results
Domenech, 2019	RCT parallel	USA and Spain	Healthy	IG: 116<br>CG: 120	65.3%	IG: 69.2±3.5<br>CG: 68.5±3.1	IG: 69.3±12<br>CG: 71.6±12.9	IG: 26.3±3.4<br>CG: 27.5±4.1	Walnuts	30-60g/d (15% energy intake)	Habitual diet	96	BW (kg)<br>WC (cm)	Not significant changes
Estruch, 2016	RCT parallel	Spain	CVD or at CV risk	IG: 2454<br>CG. 2450	57%	IG: 33.6±13<br>CG: 35±13	IG: 83.5±13<br>CG: 82.2±14.6	IG: 30.3±3.2<br>CG: 30.6±3.9	Mixed nuts	30g/d: 15g walnuts, 7.5g almonds and 7.5g hazelnuts	Low-fat diet	240	BW (kg)<br>WC (cm)	Significant decrease in BW (p=0.048) and WC (p=0.006)
Fantino, 2019	RCT parallel	France	Healthy	IG: 30<br>CG: 30	100%	IG: 30±1 (SEM)<br>CG: 33±1 (SEM)	IG: 62.7±1.7 (SEM)<br>CG: 61.44±1.41 (SEM)	IG: 23.6±0.59 (SEM)<br>CG: 23.42±0.49 (SEM)	Pistachios (dry, roasted, unsalted, shelled)	44g/d (250kcal) as snacks in the morning	Habitual diet	12	BW (kg)	BW and body composition were unchanged in both groups
Fatahi, 2019	RCT parallel	Iran	OW/obese	IG:33<br>CG1: 33<br>CG2:33	100%	IG: 52.9±1.5<br>CG1: 54±1.6<br>CG2: 54.5±1.6	IG: 85.6±4.3<br>CG1: 83.9±5.1<br>CG2: 84.7±5.1	IG: 33.3±5.8<br>CG1: 32.98±5.73<br>CG2: 33.6±NR	Walnuts	10.2g/d plus low calorie diet (18 walnuts/week)	Low calorie diet (plus 300g/wk of fish or plus fish 150g/wk and 9 walnuts/wk)	12	BW (kg)<br>WC (cm)	Not significant changes
Foster, 2012	RCT parallel	USA	OW/obese	IG: 61<br>CG: 62	91.1%	IG: 47±12<br>CG: 46.7±13	IG: 94±13.1<br>CG: 91.5±11.9	IG: 31.06±2.8<br>CG: 30.0±3.3	Almonds	28g/d	Hypocaloric diet	72	BW (kg)<br>Fat mass (kg)	Participants in the almond-enriched diet lost significantly less BW than nut-free diet participants at 6 months, not significant changes at 18 months
Gebauer, 2008	RCT crossover	USA	Hypercholesterolemia/hyperlipidemic	IG: 28	68.3%	48±1.5	76.8±2.6	26.8±0.7	Pistachios	15% energy intake	Low-fat diet	4	BW (kg)	Not significant changes
Ghadiminouran, 2009	RCT crossover	Iran	Hypercholesterolemia/hyperlipidemic	IG: 54	0%	43 (SE: 1.3)	IG:78 (2.1)<br>CG: 78.1 (2.1)	27.5 (0.5)	Peanuts (roasted, salted)	60-93g/d (20% energy intake)	Habitual diet	4	BW (kg)	Not significant change

Author, Year	Design	Country	Study population	n	% women	Age (mean/sd; range)	Baseline BW	Baseline BMI	Type of nut	Nut consumption (g/d; kcal/d; %energy)	Control	Duration (wks)	Outcome	Main results
Gozde, 2019	RCT parallel	Turkey	Hypercholesterolemia/hyperlipidemic	IG: 20<br>CG: 17	43.2%	IG: 47.1±5.44<br>CG: 43.3±6.2	IG: 73.3±11.1<br>CG: 73.6±14.45	IG: 25.6±2.04<br>CG: 25.5±3.2	Walnuts	40g/d	Low-fat diet (AHA)	6	BW (kg)<br>BMI (kg/m2)<br>WC (cm)<br>WHR<br>FM (kg)<br>BF%	Not significant changes
Gulati, 2014	RCT parallel	India	MetS	IG: 30<br>CG: 30	45.6%	IG: 29.9±1.2<br>CG: 29.4±1.7	IG: 81.6±13<br>CG: 80.3±10.3	-	Pistachios	20% energy intake	Guideline-based diet	24	BW (kg)<br>WC (cm)	Not significant changes
Hernández-Alonso, 2014	RCT crossover	Spain	Healthy	IG: 54	46.3%	IG: 55 (53.4 to 56.8)	(95%CI): 77.6 (74.8 to 80.3)	BMI (95%CI): 28.9 (28.2 to 29.6)	Pistachios (half roasted and half salted)	57g/d	Isocaloric and matched for nutrients diet	16	BW (kg)<br>BMI (kg/m2)<br>WC (cm)	Not significant treatment effect for both groups
Hollis, 2007	RCT crossover	USA	Healthy	IG: 20	100%	24±9	70.2±10.1	25.9±3.1	Almonds (raw, unsalted)	1440kJ/d	Habitual diet	10	BW (kg)<br>BMI (kg/m2)<br>BF%<br>Fat mass (kg)	Not significant changes
Hou, 2018	RCT parallel	China	T2DM	IG1: 11<br>IG2: 14	40%	IG1: 68±5.8<br>IG2: 70.86±8.2	-	IG1: 22.84±2.48<br>IG2: 24.08±3.15	IG1: Peanuts;<br>IG2: Almonds	IG1: Peanuts (50-60g/d)<br>IG2: Almonds (45-55g/d) (unsalted, with skin, no processed)	Low carbohydrate diet for both interventions	12	BMI (kg/m2)	Both interventions did not increase BMI
Hwang, 2019	RCT crossover	Korea	MetS	n: 84	50%	39.44±6.53	76.1±14.9	27.1±3.6	Walnuts	45g/d	Isocaloric white bread	16	WC (cm)<br>Central obesity,<br>Reversion rates for MetS	28.6%-52.8% reversion rates for individual MetS components and 51.2% of participants with MetS at baseline reverted to a normal status after the walnut intervention

Author, Year	Design	Country	Study population	n	% women	Age (mean/sd; range)	Baseline BW	Baseline BMI	Type of nut	Nut consumption (g/d; kcal/d; %energy)	Control	Duration (wks)	Outcome	Main results
Jamshed, 2015	RCT crossover	Pakistan	CVD or at CV risk	IG1: 38<br>IG2: 41<br>CG: 34	24.7%	60 (32-86 y)	(SEM)<br>IG1: 79±0.2<br>IG2: 75±0.2<br>CG: 73.4±0.2	-	Almonds (soaked overnight, without skin)	10g/d (IG1: Pakistani almonds; IG2: American almonds)	Habitual diet	12	BW (kg)	Not significant changes
Jenkins, 1997	RCT crossover	Canada	Healthy	IG: 10	30	33±4 (SEM)	IG: 71.3±5.2;<br>CG: 72.6±5.2	BMI: 23±1	Mixed	60-120 g/d	Habitual diet	2	BW (kg)	Not significant change (p=0.38)
Jenkins, 2002	RCT crossover	Canada	Hypercholesterolemia/hyperlipidemic	IG: 27	44.4%	IG: 64±9	CG: 71.0±2.4;<br>IG1: 71.1±2.4;<br>IG2: 71.2±2.5	BMI:25.7±3	Almonds	73+/-3 g/d	NCEP Step II	4	BW (kg)	Not significant reduction
Jenkins, 2011	RCT parallel	Canada	T2DM	IG: 40<br>CG: 39	34%	IG: 63±9<br>CG: 61±10	IG: 80±15<br>CG: 83±15	IG: 29±5<br>CG: 29±4	Mixed (raw almonds, pistachios, walnuts, pecans, hazelnuts, peanuts, cashews and macadamias)	75g/d	Isocaloric and isoprotein sugarfree muffins	12	BW (kg)	Not significant changes from baseline in body BW
Johnston, 2013	RCT parallel	USA	OW/obese	IG: 23<br>CG: 21	63.7%	20-65 y	IG: 87±3.9<br>CG: 82.5±3.7	-	Peanuts	28g/d (170kcal), consumed 1h prior dinner meal	Low fat diet + Grain bar, 140kcal	8-16	BW (kg)<br>BF%<br>WC (cm)	BW was reduced for control versus peanut group after eight weeks (p = 0.03). Decrement on BF% and WC not significant
Jung, 2017	RCT crossover	Korea	OW/obese	IG: 84	86.9%	52±0.6	-	25.4±0.22	Almonds (roasted)	56g/d	Isocaloric cookies	4	BW (kg)<br>BMI (kg/m2)<br>WC (cm)<br>FM (kg)	Not significant changes

Author, Year	Design	Country	Study population	n	% women	Age (mean/sd; range)	Baseline BW	Baseline BMI	Type of nut	Nut consumption (g/d; kcal/d; %energy)	Control	Duration (wks)	Outcome	Main results
Kalgaonkar, 2010	RCT parallel	USA	Polycystic ovary syndrome	IG1: 14<br>IG2. 17	100%	IG: 36.2±1.7<br>IG2: 31.2±1.7	IG1: 97.1±6.2<br>IG2: 101±5.3	IG1: 35.1±1.8<br>IG2: 35.2±1.6	Almonds vs Walnuts	31g/d of total fat (46g/d almonds vs 36g/d walnuts)		6	BW (kg)<br>BMI (kg/m2)	Not significant change
Kaseb, 2013	RCT crossover	Iran	T2DM	24	61.9%	52.6±7.1	74.35±12.24	26.94±2.89	Almonds vs Walnuts	40cc of almond oil; 40cc of walnut oil	Step I diet	4	BW (kg)	Not significant changes
Kasliwal, 2015	RCT parallel	India	Hypercholesterolemia/hyperlipidemic	IG: 21<br>CG: 21	18.1%	IG: 37.7±7.6<br>CG: 40.4±8.2	IG: 77.1±12.4<br>CG: 80.4±15.7	IG: 26.1±2.9<br>CG: 27.8±4.7	Pistachios (shelled)	40g/d	Healthy diet	12	BW (kg)<br>BMI (kg/m2)<br>WC (cm)	Not significant changes
Katz, 2012	RCT crossover	USA	OW/obese	IG: 40	60.9%	57.4±11.9	-	33.2±4.4	Walnuts (shelled, raw)	56g/d (as snack or with a meal)	Ad libitum diet	8	BW (kg)<br>BMI (kg/m2)<br>WC (cm)	The control diet was associated with a reduction in BMI (p=0.02) and body BW (p=0.02) relative to the walnut-enriched diet
Kocyigit, 2006	RCT parallel	Turkey	Healthy	IG: 44	38.7%	IG: 33.4±7.2;<br>CG: 32.8±6.7	-	IG: 24.2±6.1;<br>CG: 24.6±5.6	Pistachios	20% energy intake, 65-75g/d	Habitual diet	3	BMI (kg/m2)	Not significant change (p>0.05)
Lamarche, 2004	RCT crossover	Canada	Hypercholesterolemia/hyperlipidemic	IG:12	50%	IG: 65±3	70±13.7	25.8±3.4	Almonds	15g/4·2MJ	Low fat diet	4	BW (kg)	Significant reduction (p=0.02)
Le, 2016	RCT parallel	USA	OW/obese	IG: 35<br>CG1: 36<br>CG2: 31	100%	IG: 51 (22-67)<br>CG1: 50 (25-68)<br>CG2: 50 (25-72)	-	IG: 33.6 (27-40)<br>CG1: 33.2 (27-40)<br>CG2: 33.6 (27-40)	Walnuts	42g/d	Hypocaloric diet (Lower fat CG1; Lower carbohydrate CG2)	24	BW (kg)<br>BMI (kg/m2)	Similar and significant BW loss across the diet groups, walnut-enriched diet resulted in the most favourable changes in lipid level
Lee, 2014	RCT parallel	Korea	MetS	IG: 30<br>CG: 30	100%	35-65y	IG: 73±11.34<br>CG: 73±11.12	IG: 27.2±2.11<br>CG: 27±2.16	Mixed nuts	30g/d (15 g raw walnuts, 7.5g of roasted peanuts and raw pine nuts)	Dietary recommendations for prudent diet (keeping their usual diet)	6	BW (kg)<br>BMI (kg/m2)<br>WC (cm)	Not significant treatment effect for both groups

Author, Year	Design	Country	Study population	n	% women	Age (mean/sd; range)	Baseline BW	Baseline BMI	Type of nut	Nut consumption (g/d; kcal/d; %energy)	Control	Duration (wks)	Outcome	Main results
Lee, 2017	RCT crossover	USA	OW/obese	IG: 31	41.9%	46.3±1.8	-	29.6±0.5 (SEM)	Almonds	42.5g/d	Control isocaloric diet	4	BW (kg)	Data post no reported: BW loss -1.4(1.7kg), no different between treatments
Li, 2010	RCT parallel	USA	OW/obese	IG: 27<br>CG: 25	80.5%	IG: 45.4±2<br>CG: 47.3±2.3	IG: 86 (3.2)<br>CG: 85.5 (4.8)	IG: 30.1 (0.4)<br>CG: 30.9 (0.4)	Pistachios (salted)	53g/d (240kcal)	BW reduction diet plus pretzels	12	BW (kg)<br>BMI (kg/m2)	Both groups lost BW, significant differences in BMI between groups
Li, 2011	RCT crossover	Taiwan	T2DM	IG: 20	55%	58±2	-	26±0.7	Almonds	20% energy intake	NCEP Step II	8	BMI (kg/m2)<br>BF%	BF% was significantly lower in patients consuming almonds (p=0.002)
Liu, 2017	RCT parallel	South Korea	Healthy	IG1: 58<br>IG2: 55<br>CG: 56	54%	26.3±5.6	IG1:65.5±12.6<br>IG2:63.9±12.8<br>CG: 62.3±11.5	IG: 23±2.9<br>IG2:22.9±3.1<br>CG: 21.9±3.15	Almonds	IG1: Pre-meal almonds, 56 g/d<br>IG2: Snack almond, 56g/d	High carbohydrate isocaloric control	16	BW (kg)<br>BMI (kg/m2)<br>FM (kg)<br>BF%<br>Visceral fat	Significant decrease in FM compared to control<br>Significant effect on changes of FM, BF% and visceral fat.
Ma, 2010	RCT crossover	USA	T2DM	IG: 21	58.3%	58.1±9.2	89±15.5	32.5±5	Walnuts	56g/d (366kcal)	Habitual diet	8	BW (kg)<br>BMI (kg/m2)<br>WC (cm)	Not significant changes
Maranhão, 2011	RCT parallel	Brazil	OW/obese	IG: 8<br>CG: 9	100%	15.4±2	IG: 86.3 (82.2-94.3)<br>CG: 91.7 (82.1-110.8)	IG: 35.3 (33.9-36)<br>CG: 34 (33-39.1)	Brazil nuts	15-25g/d	Placebo capsule (lactose)	16	BW (kg)<br>BMI (kg/m2)<br>WC (cm)	Not significant changes
Mercanligil, 2007	RCT crossover	Turkey	Hypercholesterolemia/hyperlipidemic	IG:15	0%	48±8	74.3±5	26.1±1.6	Hazelnuts	40g/d; 11.6% energy intake	Low-fat and cholesterol, high carbohydrate diet	8	BW (kg)<br>BMI (kg/m2)<br>WHR<br>BF%	Significant reduction of % body fat
Mohan, 2018	RCT parallel	India	T2DM	IG: 129<br>CG: 140	46.1%	IG: 51.3±8.8<br>CG: 50.4±10	IG: 67.6±9.1<br>CG: 67.3±11.5	IG: 25.6±2.8<br>CG: 26.2±3.9	Cashews (raw, unsalted)	30g/d as snack (11-13& energy intake) + Diabetic control diet	Diabetic control diet	12	BW (kg)<br>BMI (kg/m2)<br>WC (cm)	No differences between groups on BW or BMI

Author, Year	Design	Country	Study population	n	% women	Age (mean/sd; range)	Baseline BW	Baseline BMI	Type of nut	Nut consumption (g/d; kcal/d; %energy)	Control	Duration (wks)	Outcome	Main results
Morgan, 2000	RCT parallel	USA	Healthy	IG: 10<br>CG: 9	78.9%	IG: 37±12;<br>CG: 45±10	-	IG: 24±5;<br>CG: 24±4	Pecans	68g/d	Habitual diet	8	BMI (kg/m2)	Not significant change
Mukuddem-Petersen, 2007	RCT parallel	South Africa	MetS	IG1: 21<br>IG2: 21<br>CG:22	54.7%	IG1: 45<br>IG2: 46<br>CG: 45	IG1: 107±31.8<br>IG2: 99±27.6<br>CG: 106±28.2	IG1: 36±11.02<br>IG2: 34.4±8.98<br>CG: 35.1±9.39	Walnuts	20% energy intake	Habitual diet	8	BW (kg)	Not significant changes
Njike, 2015	RCT crossover	USA	Prediabetes	IG: 112	72.3%	55	82.6 (mean)	-	Walnuts	56g/d (366kcal)	Habitual diet	24	BW (kg)<br>BMI (kg/m2)<br>WC (cm)<br>BF%<br>Visceral fat	Not significant changes
Njike, 2015	RCT parallel	USA	OW/obese	IG: 16<br>CG: 16	62%	IG: 59.5±9<br>CG: 56.5±7.6	IG: 86.8±13.3<br>CG: 81.3±14.3	IG: 34.6±7.2<br>CG: 34.4±5.7	Mixed nuts (snack bar)	1-4portions (200-800kcal)	Isocaloric snack	12	BW (kg)<br>BMI (kg/m2)<br>WC (cm)<br>BF%<br>Visceral fat	Significant reduction of body fat and visceral fat on nut-based snack
O'Byrne, 1997	RCT parallel	USA	Hypercholesterolemia/hyperlipidemic	IG: 12;<br>CG: 13	100	55-65	IG: 68.6±6.2<br>CG: 71.0±10.5	IG: 26.2±3.8;<br>CG: 26.4±3.3	Peanuts	35-68 g/d	Low-fat diet	24	BW (kg), BMI (kg/m2), BF%	Significant reduction for all adiposity outcomes (p<0.05)
Olmedilla-Alonso, 2008	RCT crossover	Spain	CVD or at CV risk	IG: 25	40%	54.4±8.1	82.1±12.9	30±3.8	Walnuts	19.4g/d	Habitual diet	5	BW (kg)	Significant reduction of BW (-0.5, -0.96 to -0.05), not significant at comparison between control (p=0.9)
Orem, 2013	RCT crossover	Turkey	Hypercholesterolemia/hyperlipidemic	IG: 21	14.3%	44.6±10.4	81±14.1	27.4±3.1	Hazelnuts	49-86g/d (18-21% energy intake), consumed as snack	Habitual diet	4	BW (kg)<br>BMI (kg/m2)	BW and BMI showed a significant decrement in hazelnut-enriched diet when compared with control diet

Author, Year	Design	Country	Study population	n	% women	Age (mean/sd; range)	Baseline BW	Baseline BMI	Type of nut	Nut consumption (g/d; kcal/d; %energy)	Control	Duration (wks)	Outcome	Main results
Parham, 2014	RCT crossover	Iran	T2DM	IG: 23<br>CG:21	75%	IG: 53±10<br>CG: 50±11	-	IG: 32.2±6.6<br>CG: 30.24±4	Pistachios	50g/d	Habitual diet	12	BMI (kg/m2)	Not overall change in BMI
Park, 2017	RCT crossover	South Korea	MetS	IG: 84	50%	39.44±6.53	76.1±14.9	27.1±3.6	Walnuts	45g/d (305.4kcal)	Isocaloric white bread	6	WC (cm)<br>Reversion rates of MetS components and central obesity	Walnut consumption improved MetS status, resulting in 28.6%-52.8% reversion rates for MetS components
Pearson, 2016	RCT parallel	New Zealand	Healthy	IG: 29<br>CG1: 24<br>CG2: 26<br>CG3: 23	44%	IG: 39.9±14.5<br>CG1: 38.6±14.1<br>CG2: 39.8±14.3<br>CG3: 37.5±12.7	IG: 72.3±11.4<br>CG1: 67.6±10<br>CG2: 68.9±11.7<br>CG3: 69.7±12.3	IG: 24.8±2.8<br>CG1: 23±2.9<br>CG2: 23.6±3.4<br>CG3: 24.1±3	Hazelnuts (raw)	42g/d	CG1: no snack; CG2: Isocaloric chocolate snack; CG3: Isocaloric potato crisps snack	12	BW (kg)<br>BMI (kg/m2)	Not significant changes
Razquin, 2010	RCT parallel	Spain	CVD or at CV risk	IG: 239<br>CG. 196	55%	IG: 67.6±6.7<br>CG: 68.3±6.0	IG: 74.6±10.3<br>CG: 74.5±11.8	IG: 29.1±3.1<br>CG: 29.2±3.5	Mixed nuts	Walnuts 15g/d; hazelnuts 7.5g/d; almonds 7.5g/d	Low-fat diet	144	BW (kg)	Not significant BW changes
Ren, 2020	RCT parallel	China	T2DM	IG: 22<br>CG: 23	55.7%	IG: 73.6±5<br>CG: 70.5±5.9	IG: 66.6±8.8<br>CG: 63.1±12.9	IG: 23.5±2.3<br>CG: 23.7±2.8	Almonds	56g/d	Low-fat diet	12	BW (kg),<br>BMI(kg/m2)	Significant decrease of BW and BMI on intervention group
Rock, 2017	RCT parallel	USA	OW/obese	IG: 49<br>CG: 51	58%	IG: 53.3±1.4<br>CG: 52.2±1.6	IG: 91.1±2.3<br>CG: 90.9±1.8	IG: 32.4±0.5<br>CG: 32.4±0.4	Walnuts	28-42g/d (15% energy intake)	Hypocaloric standard diet	24	BW (kg)<br>BMI (kg/m2)<br>WC (cm)	Both groups reduced BW, BMI and WC, not differences between groups
Rock, 2020	RCT parallel	USA	OW/obese	IG: 48<br>CG: 45	62%	IG: 56.2±10.6<br>CG: 55±11.3	IG: 94.7±16.3<br>CG: 93.8±15.6	IG: 32.8±4.2<br>CG: 32.8±3.5	Pistachio	42g/d	Same diet without nuts	16	BW (kg), BMI (kg/m2), WC (cm)	Similar decrease of anthropometric measures on intervention and control group
Ros, 2004	RCT crossover	Spain	Hypercholesterolemia/hyperlipidemic	IG: 20	60%	55	70.6±10.3	-	Walnuts	40-65g/d (32% of energy from monosaturated fat)	Mediterranean diet	4	BW (kg)	No change (p>0.1)

Author, Year	Design	Country	Study population	n	% women	Age (mean/sd; range)	Baseline BW	Baseline BMI	Type of nut	Nut consumption (g/d; kcal/d; %energy)	Control	Duration (wks)	Outcome	Main results
Ruisinger, 2015	RCT parallel	USA	CVD or at CV risk	IG: 22<br>CG: 26	50%	IG: 60±10.4<br>CG: 59.3±11.7	IG: 86.8±13.3<br>CG: 81.3±14.3	IG: 29.8±4.8<br>CG: 28.6±3.9	Almonds	100g/d	ATP-III TLC Diet	4	BW (kg)<br>BMI (kg/m2)	Not significant changes
Sabaté, 2003	RCT crossover	USA	Healthy	IG: 25	44	41±13	71±2.7		Almonds	68g/d; 10-20% (low and high almond diet) of energy intake	NCEP Step I	4	BW (kg)	Not significant change (p=0.11)
Sabaté, 2005	RCT crossover	USA	Healthy	IG: 49;<br>CG: 41	55.6%	IG: 54.3±10.6	IG: 73.3±13.1;<br>CG: 78.5±15	IG: 26.1±3.5;<br>CG: 26.9±3.3	Walnuts	35g/d; 12% energy intake	Habitual diet	24	BW (kg); BMI (kg/m2); Fat mass (kg); BF%	After adjustment, no significant differences for BW, body composition, except for BMI
Salas-Salvado, 2008	RCT parallel	Spain	CVD or at CV risk	IG: 411<br>CG. 404	53.8%	IG: 67.2±5.7<br>CG: 67.9±6.2	IG: 75.8±11<br>CG: 75.4±11.3	IG: 29.3±3.2<br>CG: 29.5±3.5	Mixed nuts	Walnuts 15g/d; hazelnuts 7.5g/d; almonds 7.5g/d	Low-fat diet	48	BW (kg)<br>MetS reversion<br>Abdominal obesity reversion	Not significant change on BW occurred: -0.2±3.7 (nuts) and -0.1±3.2 (control)<br><br>OR for reversion of MetS were: 1.7 (1.1 to 2.6) for the nuts group compared with the control<br><br>Significant reversion rate of abdominal adiposity for nut group
Sauder, 2015	RCT crossover	USA	T2DM	IG: 30	50%	56±7.8	90.6±3.4	31.2±3.1	Pistachios (roasted)	59-128g/d; 20% energy intake	Control diet (Nutritionally adequeate)	4	BW (kg)<br>BMI (kg/m2)	Not significant changes between pistachio and control diet
Schutte, 2006	RCT parallel	South Africa	MetS	IG1: 20;<br>IG2: 21;<br>CG:21	54.8%	21-65	-	IG1: 35.9±6.4;<br>IG2: 34.7±5.5;<br>CG: 35.5±5.1	IG1: Walnuts;<br>IG2: unsalted cashews	63-108g/d; 20% energy intake	Habitual diet	8	BMI (kg/m2); WC (cm)	BMI and WC did not change

Author, Year	Design	Country	Study population	n	% women	Age (mean/sd; range)	Baseline BW	Baseline BMI	Type of nut	Nut consumption (g/d; kcal/d; %energy)	Control	Duration (wks)	Outcome	Main results
Sheridan, 2007	RCT crossover	USA	Hypercholesterolemia/hyperlipidemic	IG: 15	26.7%	60±3	79.4±3	28±0.9	Pistachios	15% energy intake	Habitual diet	4	BW (lbs)<br>BMI (kg/m2)	Not significant changes
Somerset, 2013	RCT parallel	Australia	OW/obese	n: 64	84.4%	IG: 43.7 (1.4)<br>CG: 43.2 (1.4)	IG: 103.6 (3.8)<br>CG: 99.6 (2.8)	IG: 35.6 (1.2)<br>CG: 35.8 (1.2)<br>(SEM)	Macadamia nuts	-	Habitual diet	10	BW (kg)<br>BMI (kg/m2)<br>BFM (kg)<br>WC (cm)	Significant decreases in WC ($p < 0.05$) for the nut group at week 10 compared to baseline
Souza, 2018	RCT parallel	Brazil	OW/obese	IG: 24<br>CG: 22	100%	20-59 y	IG: 83.6±13.5<br>CG: 81.9±14.4	IG: 33.3±4.7<br>CG: 32.5±4.4	Almonds (baru; roasted)	20g/d (109kcal) + same control diet	Individualized normocaloric and isoenergetic diet	8	BW (kg)<br>BMI (kg/m2)<br>WC (cm)<br>FM (kg)<br>BF%	Significant reduction on WC ($p=0.03$)
Spaccarotella, 2008	RCT crossover	USA	At risk for prostate cancer	IG: 21	0%	IG: 66	84.8±2.9	-	Walnuts	75g/d; 24% energy intake	Habitual diet	8	BW (kg)<br>BMI (kg/m2)	Not significant change, walnut group maintained their BW
Spiller, 1998	RCT parallel	USA	Hypercholesterolemia/hyperlipidemic	IG: 18<br>CG1: 12<br>CG2: 15	73.3%	53±10	IG: 65±13;<br>CG1: 69±15;<br>CG2: 64±11	-	Almonds	100g/d	non-low fat diet (cheddar cheese, butter, bye crackers, olive oil)	4	BW (kg)	Not significant change
Tan, 2013	RCT parallel	Australia	CVD or at CV risk	IG1: 28<br>IG2: 28<br>IG3: 26<br>IG4: 28<br>CG: 27	65%	IG1: 33±11.5<br>IG2: 28±10.7<br>IG3: 29±13.5<br>IG4: 29±12<br>CG: 29±9.6	IG: 80.5±15<br>IG2: 83.2±21.1<br>IG3: 84.8±13.7<br>IG4: 81.8±14.6<br>CG: 77.2±16.8	IG: 28.2±4.8<br>IG2: 28.7±5<br>IG3: 29±3.9<br>IG4: 28.2±5.2<br>CG: 27±4.4	Almonds	43g/d; consumed with meals or as snacks (Four different groups: breakfast (IG1); Lunch (IG2); Morning snack (IG3) and afternoon snack (IG4))	Habitual diet without nuts and seeds	4	BW (kg)<br>BMI (kg/m2)<br>BFM (kg)<br>WC (cm)	Anthropometric measurements did not differ from the control group or across intervention groups

Author, Year	Design	Country	Study population	n	% women	Age (mean/sd; range)	Baseline BW	Baseline BMI	Type of nut	Nut consumption (g/d; kcal/d; %energy)	Control	Duration (wks)	Outcome	Main results
Tapsell, 2004	RCT parallel	Australia	T2DM	IG: 58	51.1%	IG: 59.3±7.1; CG: 60.5±8.2	IG: 84.55±4.31; CG: 81.87±11.19	IG: 30.16±4.51; CG: 29.22±2.6	Walnuts	30g/d	Low-fat diet	24	BW (kg); BMI (kg/m2); BF%	No significant differences between groups for changes in body BW, BF%
Tapsell, 2009	RCT parallel	Australia	T2DM	IG: 18 CG. 17	-	54±8.7	IG: 92.3±15.7 CG: 93.4±3	IG: 33.2±4.4 CG: 33±4	Walnuts	30g/d	Low-fat diet	48	BW (kg) BF% VAT (cm2) SAT (cm2)	Not significant BW loss between groups
Tapsell, 2017	RCT parallel	Australia	OW/obese	IG: 72 CG1: 45 CG2: 61	74%	IG: 45 (IQR: 37-51)	-	-	Walnuts	30g/d	CG1: interdisciplinary advice; CG2: Usual care (advice based on Australian Guide to Healthy Eating and National Physical Activity Guidelines)	12-48	BW (kg) BF%	Walnut group lost more BW than controls at 3 months and 6 months
Tey, 2013	RCT parallel	New Zealand	OW/obese	IG1: 33 IG2: 37 CG: 37	57%	IG1: 43.8±13.5 IG2: 42.8±10.6 CG: 41±13	IG1: 86.2±11.8 IG2: 92±19.6 CG: 88.7±16.	IG1: 30.7±4.7 IG2: 30.9±6 CG: 30.4±4.5	Hazelnuts	30 or 60g/d	Habitual diet	12	BW (kg) BMI (kg/m2) BFM (kg) BF%	Not significant changes between groups
Tindall, 2019	RCT crossover	USA	CVD or at CV risk	IG: 36	44.4%	IG: 43±10	-	30.3±4.7	Walnuts (whole)	57-99g/d (18% energy intake)	Isocaloric maintenance diets without walnuts	6	BW (kg)	Significant reduction of BW after walnut diet but not significant differences between interventions
Wang, 2020	RCT parallel	China	MetS	IG: 113 CG: 111	69.7%	IG: 46.2±9.9 CG: 46.2±9.9	IG: 76.5±14.4 CG: 73.6±11.8	IG: 28.4±3.4 CG: 27.6±2.7	Peanuts	56g/d	Isocaloric snack	12	BW (kg), BMI (kg/m2), WC (cm)	No significant changes on anthropometric measures after intervention
Wien, 2003	RCT parallel	USA	OW/obese	IG:32; CG:33	57%	IG: 53±2; CG: 57±2	IG: 113±5; CG: 114±5	IG: 39±1; CG: 37±1	Almonds (unblanched)	84g/d	Low-fat diet, self-selected complex carbohydrates	24	BW (lbs); BMI (kg/m2); WC (cm); Fat mass (lbs)	Significant reduction (p<0.05)

Author, Year	Design	Country	Study population	n	% women	Age (mean/sd; range)	Baseline BW	Baseline BMI	Type of nut	Nut consumption (g/d; kcal/d; %energy)	Control	Duration (wks)	Outcome	Main results
Wien, 2010	RCT parallel	USA	Pre-diabetes	IG: 32<br>CG: 33	74%	IG: 53±9<br>CG: 54±11	IG: 82.9±14.4<br>CG: 80.5±14.4	IG: 30±5<br>CG: 29±5	Almonds	20% energy intake	ADA diet	16	BW (kg)<br>BMI (kg/m2)<br>WC (cm)	Not significant change
Wien, 2014	RCT parallel	USA	T2DM	IG: 30<br>CG: 30	50%	IG: 59±13<br>CG: 64±12	IG: 86±24.8<br>CG: 90.4±19.3	IG: 31.1±6.9<br>CG: 33.4±6.8	Peanuts	20% energy intake	ADA diet	24	BW (kg)<br>BMI (kg/m2)<br>WC (cm)	Both groups experienced mild reductions in BW, BMI, and WC during the study (P = 0.01-P = 0.03), however there were no differences between the two groups
Williams, 2019	RCT crossover	USA	OW/obese	IG: 24	62.5%	Males: 41±15<br>Females: 52±9	-	-	Almonds (whole, raw)	-	Two control diet with higher carbohydrate reference and lower carbohydrate reference diet	3	BW (kg)<br>BMI (kg/m2)<br>WC (cm)	Not significant changes
Wu, 2010	RCT parallel	China	MetS	IG: 94<br>CG: 95	44.4%	IG: 48	IG: 72.2±11.4<br>CG: 70.6±10.9	IG: 25.7±2.9<br>CG: 25.4±2.4	Walnuts	30g/d	Diet according to the AHA guidelines	12	BW (kg)<br>WC (cm)	Not significant differences between groups
Yilmaz, 2019	RCT parallel	Turkey	Hypercholesterolemia/hyperlipidemic	IG: 9<br>CG: 10	100%	IG: 52±6.4<br>CG: 52±3.5	IG: 78.5±12.5<br>CG: 81.9±12.8	IG: 35.7±4.7<br>CG: 36±6.1	Hazelnuts	50g/d	Cardioprotective control diet	6	BW (kg)<br>BMI (kg/m2)<br>WC (cm)<br>WHR<br>FM (kg)<br>BF%	Not significant changes on anthropometric measurements, only BMI decreased significantly in all groups at the end
Zambón, 2000	RCT crossover	Spain	Hypercholesterolemia/hyperlipidemic	IG: 49	55.1%	56±11	70.6±12.1	27±3.1	Walnuts	-	Mediterranean diet	6	BW (kg)	Not significant reduction
Zibaeenezzhad, 2019	RCT parallel	Iran	T2DM	IG: 45<br>CG: 45	52.2%	IG: 55.5±10.8<br>CG: 54±11.4	IG: 75.9±8.6<br>CG: 73.8±7.2	IG: 27.6±2.5<br>CG: 27.2±2.3	Walnuts (oil)	15g/d	Habitual diet	12	BW (kg)<br>BMI (kg/m2)	Improvement on blood glucose level but no changes for body weight after nut consumption

RCT: randomised controlled trial; **ADA**, American Diabetes Association; **AHA**, American Heart Association; **ATP**, Adult Treatment Panel; **NCEP**, National Cholesterol Education Program; **TLC**, Therapeutic Lifestyle Changes; **T2DM**: Type 2 diabetes mellitus; **CVD**: cardiovascular disease; **ATP-III TLC Diet**: Adult Treatment Panel's third report Therapeutic Lifestyle Changes Diet counselling; **MetS**: Metabolic syndrome; **BW**: body weight; **BMI**: body mass index; **BF%**: body fat percentage; **WC**: waist circumference; **WHR**: Waist-to-hip ratio; **VAT**: Visceral adipose tissue; **SAT**: subcutaneous adipose tissue; **BFM**: Body fat mass.

Table S4. Transitivity assessment.

	Walnut (s:30; n:1728)		Almond (s:29; n:989)		Mixed nuts (s:13; n:3431)		Pistachio (s:12; n:350)		Peanut (s:9; n:346)		Hazelnut (s:8; n:189)		Cashew (s:4; n:204)		Others: brazil nut, macadamia, pecan (s:4; n:98)		p-value
	Mean	SD	Mean	SD	Mean	SD	Mean	SD	Mean	SD	Mean	SD	Mean	SD	Mean	SD	
**Baseline Age**	53.84	8.6	50.17	12.57	51.72	15.13	45.19	10.76	48.69	17.37	44.65	6.68	49.75	4.47	34.53	16.59	0.12
**Baseline BW**	76.24	13.49	76.45	11.7	79.0	6.24	78.31	11.46	82.18	10.24	75.51	7.55	81.7	21.14	95.65	5.59	0.38
**Baseline BMI**	29.44	3.26	27.68	3.34	29.36	2.72	28.46	2.97	28.41	3.33	27.35	4.31	31.35	4.68	31.15	5.2	0.22
**Baseline WC**	99.5	9.17	96.18	10.51	101.03	6.46	97.22	15.08	95.17	18.63	108.1	NA	103.6	10.55	96.83	5.83	0.87
**Baseline BF%**	32.35	6.39	34.23	5.96	37.22	2.05	24.9	NA	30.45	5.73	34.5	8.62	NA	NA	43.7	NA	0.34

BF%: Body fat percentage; **BMI**: Body mass index; **BW**: body weight, **NA**: not available; **SD**: standard deviation; **WC**: waist circumference.

Table S5. Transitivity assessment regarding the Intervention groups

Reference	Intervention	n	Baseline age	Baseline BW	Baseline BMI	Baseline WC	Baseline BF%
Abazarfard, 2014	Almond	50	42.4	76.4	29.9	107.8	NA
Abbaspour, 2019	Mixed nuts	24	30.4	90.3	30.7	98.3	37.8
Abdrabalnabi, 2020	Walnut	324	69.1	73.5	NA	NA	NA
Álvarez-Pérez, 2016	Mixed nuts	102	NA	80.2	31.1	102.6	36.4
Alves, 2014	Peanut	22	27	95.1	30	NA	NA
Barbour, 2015	Peanut	61	65	87.7	30.6	100	NA
Bashan, 2018	Walnut	73	41	86.3	30.9	NA	NA
Bento, 2014	Almond	20	35	64	23.2	NA	35.7
Berryman, 2015	Almond	48	50	74.7	26.2	93.2	NA
Bitok, 2018	Walnut	159	69.7	77.1	27.5	99.4	25.53
Bowen, 2019	Almond	39	60.8	102	34.4	112	NA
Bulló, 2009	Mixed nuts	70	68.4	NA	29.4	NA	NA
Caldas, 2020	Peanut	21	28	92.7	29.4	NA	NA
Canales, 2007	Walnut	22	54.8	81	29.6	NA	NA
Carughi, 2019	Pistachio	30	NA	58.5	NA	74.2	25
Casas-Agustench, 2011	Mixed nuts	25	52.9	86.4	31.6	105.6	36.5
Chen, 2017	Almond	33	55	65.3	25.6	89	31
Chiang, 2012	Walnut	25	NA	70.9	24.8	NA	NA
Chisholm, 2005	Mixed nuts	28	48	74.2	26.8	NA	NA
Claesson, 2009	Peanut	13	68.4	68.7	22.2	73.9	22.6
Coates, 2020	Almond	63	64	84.4	30.3	101.2	35.8
Costa e Silva, 2019	Brazil nuts	15	NA	NA	32.2	109.1	39.9
Damasceno, 2011	Almond	18	56	70.7	25.7	NA	NA
Damasceno, 2011	Walnut	18	56	70.7	25.7	NA	NA
Damasceno, 2013	Mixed nuts	58	NA	75.4	29	99	NA
Damavandi, 2013	Hazelnut	23	55.7	72.1	28.5	NA	NA
Damavandi, 2019	Cashew	22	53.86	72.1	28.7	91.9	NA
Dhillon, 2016	Almond	36	33.6	83.5	30.3	88.1	NA
Dhillon, 2018	Almond	38	NA	71.5	25.6	80.5	26.9
Dikariyanto, 2020	Almond	54	56.3	NA	27.3	94.1	32.2
Domenech, 2019	Walnut	116	69.2	69.3	26.3	96.6	NA

Estruch, 2016	Mixed nuts	2454	33.6	83.5	30.3	100.2	NA
Fantino, 2019	Pistachio	29	30	62.7	23.6	NA	NA
Fatahi, 2019	Walnut	33	52.9	85.6	33.3	108	NA
Foster, 2012	Almond	61	47	94	31.1	NA	NA
Gebauer, 2008	Pistachio	28	48	76.8	26.8	NA	NA
Ghadiminouran, 2009	Peanut	54	43	78	27.5	NA	NA
Gozde, 2019	Walnut	20	47.1	73.3	25.6	83.5	28.2
Gulati, 2014	Pistachio	30	29.9	81.6	NA	102.6	NA
Hernández-Alonso, 2014	Pistachio	54	NA	77.6	28.9	88.4	NA
Hollis, 2007	Almond	20	24	70.2	25.9	NA	35.8
Hou, 2018	Peanut	11	68	NA	22.8	NA	NA
Hou, 2018	Almond	14	70.86	NA	24.1	NA	NA
Hwang, 2019	Walnut	84	39.44	76.1	27.1	88.6	NA
Jamshed, 2015	Almond	41	NA	79	NA	NA	NA
Jenkins, 1997	Mixed nuts	10	33	71.3	23	NA	NA
Jenkins, 2002	Almond	27	64	71.1	25.7	NA	NA
Jenkins, 2011	Mixed nuts	40	63	80	29	NA	NA
Johnston, 2013	Peanut	23	NA	87	NA	NA	NA
Jung, 2017	Almond	84	52	NA	25.4	88.6	NA
Kalgaonkar, 2010	Almond	14	36.2	87.1	35.1	NA	NA
Kalgaonkar, 2010	Walnut	17	31.2	101	35.2	NA	NA
Kaseb, 2013	Almond	19	52.6	74.35	26.9	NA	NA
Kaseb, 2013	Walnut	8	52.6	74.35	26.9	NA	NA
Kasliwal, 2015	Pistachio	21	37.7	77.1	26.1	111.6	NA
Katz, 2012	Walnut	40	57.4	NA	33.2	92.7	NA
Kocyigit, 2006	Pistachio	22	33.4	NA	24.2	NA	NA
Lamarche, 2004	Almond	12	65	70	25.8	NA	NA
Le, 2016	Walnut	35	NA	33.6	33.6	NA	NA
Lee, 2014	Mixed nuts	30	NA	73	27.2	105	NA
Lee, 2017	Almond	31	46.3	NA	29.6	NA	NA
Li, 2010	Pistachio	27	45.4	86	30.1	NA	NA
Li, 2011	Almond	20	58	NA	26	NA	30.6
Liu, 2017	Almond	58	26.3	65.5	23	NA	27.1
Ma, 2010	Walnut	22	58.1	89	32.5	NA	NA

Maranhão, 2011	Brazil nuts	8	15.4	86.3	35.3	91	NA
Mercanligil, 2007	Hazelnut	15	48	74.3	26.1	NA	26.2
Mohan, 2018	Cashew	129	51.3	67.6	25.6	111.5	NA
Morgan, 2000	Pecan	10	37	NA	24.5	NA	NA
Mukuddem-Petersen, 2007	Walnut	21	45	107	36	NA	NA
Mukuddem-Petersen, 2007	Cashew	21	45	99	34.4	NA	NA
Njike, 2015	Walnut	112	55	82.6	NA	NA	NA
Njike, 2015	Mixed nuts	16	59.5	86.8	34.6	88.42	43.9
O'Byrne, 1997	Peanut	12	NA	68.6	26.2	NA	34.6
Olmedilla-Alonso, 2008	Walnut	25	54.4	82.1	30	NA	NA
Orem, 2013	Hazelnut	21	44.6	81	27.4	NA	NA
Parham, 2014	Pistachio	23	53	NA	32.2	NA	NA
Park, 2017	Walnut	84	39.44	76.1	27.1	115.5	NA
Pearson, 2016	Hazelnut	32	39.9	72.3	24.8	NA	NA
Razquin, 2010	Mixed nuts	239	67.6	74.6	29.1	NA	NA
Ren, 2020	Almond	22	73.6	66.6	23.5	NA	NA
Rock, 2017	Walnut	49	53.3	91.1	32.4	108.4	NA
Rock, 2020	Pistachio	48	56.2	94.7	32.8	109.3	NA
Ros, 2004	Walnut	20	55	70.6	NA	NA	NA
Ruisinger, 2015	Almond	22	60	86.8	29.8	NA	NA
Sabaté, 2003	Almond	25	41	71	NA	NA	NA
Sabaté, 2005	Walnut	49	54.3	73.3	26.1	NA	NA
Salas-Salvado, 2008	Mixed nuts	411	67.2	75.8	29.3	NA	31.7
Sauder, 2015	Pistachio	30	56	90.6	31.2	NA	NA
Schutte, 2006	Walnut	20	NA	NA	35.9	108.3	NA
Schutte, 2006	Cashew	21	NA	NA	34.7	101.2	NA
Sheridan, 2007	Pistachio	15	60	79.4	28	NA	NA
Somerset, 2013	Macadamia	64	43.7	103.6	35.6	94.6	NA
Souza, 2018	Almond	24	NA	83.6	33.3	85.3	49.1
Spaccarotella, 2008	Walnut	21	66	84.8±2.9	NA	NA	NA
Spiller, 1998	Almond	18	53	65	NA	NA	NA
Tan, 2013	Almond	28	33	80.5	28.2	85.6	NA
Tan, 2013	Almond	26	28	83.2	28.7	86.2	NA
Tan, 2013	Almond	28	29	84.8	29	81.9	NA

Tan, 2013	Almond	28	29	81.8	28.2	95.7	NA
Tapsell, 2004	Walnut	58	59.3	84.55	30.2	NA	35.1
Tapsell, 2009	Walnut	18	54	92.3	33.2	NA	43
Tapsell, 2017	Walnut	72	NA	NA	NA	NA	41.4
Tey, 2013	Hazelnut	33	43.8	86.2	30.7	NA	35.4
Tey, 2013	Hazelnut	37	42.8	92	30.9	NA	35
Tindall, 2019	Walnut	36	43	NA	30.3	NA	NA
Wang, 2020	Peanut	113	46.2	76.5	28.4	NA	NA
Wien, 2003	Almond	32	53	113	39	120	NA
Wien, 2010	Almond	32	53	82.9	30	95	NA
Wien, 2014	Peanut	30	59	86	31.1	104.3	NA
Williams, 2019	Almond	24	NA	NA	NA	NA	NA
Wu, 2010	Walnut	94	48	72.2	25.7	NA	NA
Yilmaz, 2019	Hazelnut	9	52	78.5	35.7	104.1	43.1
Zambón, 2000	Walnut	49	56	70.6	27	NA	NA
Zibaeenezzhad, 2019	Walnut	45	55.5	75.9	27.6	NA	37.8

Table S6. Transitivity assessment regarding the Control groups.

Reference	Intervention	n	Mean age	Baseline body weight	Baseline BMI	Baseline WC	Baseline BF%
Abazarfard, 2014	Control	50	42.9	75.6	29.4	106.2	NA
Abbaspour, 2019	Control	24	29.1	95.1	31.6	101.4	39.1
Abdrabalnabi, 2020	Control	312	69.1	73.5	NA	NA	NA
Álvarez-Pérez, 2016	Control	98	NA	79.3	31.3	103.4	37.1
Alves, 2014	Control	22	27.4	94.5	29.5	NA	NA
Barbour, 2015	Control	61	65	87.7	30.6	100	NA
Bashan, 2018	Control	72	40.7	85	30.1	NA	NA
Bento, 2014	Control	20	35	63.7	23.1	NA	34.6
Berryman, 2015	Control	48	50	74.7	26.2	93.2	NA
Bitok, 2018	Control	148	69.1	75.6	27.4	98.6	25.4
Bowen, 2019	Control	37	60.6	95.8	33.2	108	NA
Bulló, 2009	Control	59	67.8	NA	29.6	NA	NA
Caldas, 2020	Control	22	27.4	94.5	30.1	NA	NA
Canales, 2007	Control	22	54.8	81	29.6	NA	NA
Carughi, 2019	Control	30	NA	57.3	NA	74.8	24.9
Casas-Agustench, 2011	Control	25	50.6	79.9	30	101.3	34.4
Chen, 2017	Control	33	55	64.7	25.3	88.1	31.6
Chiang, 2012	Control	25	NA	70.9	24.8	NA	NA
Chisholm, 2005	Control	28	48	74.1	26.8	NA	NA
Claesson, 2009	Control	12	67.8	67.3	22.2	74.6	26.4
Coates, 2020	Control	65	65	85.4	30.5	102.3	35.5
Costa e Silva, 2019	Control	16	NA	NA	30.8	102.3	43.7
Damasceno, 2011	Control	18	56	70.7	25.7	NA	NA
Damasceno, 2013	Control	52	NA	77.6	29.7	100.2	NA
Damavandi, 2013	Control	25	55.7	72	28.2	NA	NA
Damavandi, 2019	Control	21	53.86	71.9	28.6	93.2	NA
Dhillon, 2016	Control	37	35	82.2	30.6	90.2	NA
Dhillon, 2018	Control	35	NA	71.3	25.3	80.4	27.9
Dikariyanto, 2020	Control	51	56	NA	26.7	93.3	31.1
Domenech, 2019	Control	120	68.5	71.6	27.5	99.2	NA
Estruch, 2016	Control	2450	35	82.2	30.6	100.9	NA
Fantino, 2019	Control	29	33	61.4	23.42	NA	NA

Fatahi, 2019	Control	33	54	83.9	32.98	109	NA
Foster, 2012	Control	62	46.7	91.5	30	NA	NA
Gebauer, 2008	Control	28	48	76.8	26.8	NA	NA
Ghadiminouran, 2009	Control	54	43	78.1	27.5	NA	NA
Gozde, 2019	Control	17	43.3	73.6	25.5	84.1	25.9
Gulati, 2014	Control	30	29.4	80.3	NA	106	NA
Hernández-Alonso, 2014	Control	54	NA	77.6	28.9	89	NA
Hollis, 2007	Control	20	24	70.2	25.9	NA	33.6
Hwang, 2019	Control	84	39.44	76.1	27.1	89.5	NA
Jamshed, 2015	Control	34	NA	73.4	NA	NA	NA
Jenkins, 1997	Control	10	33	72.6	23	NA	NA
Jenkins, 2002	Control	27	64	71	25.7	NA	NA
Jenkins, 2011	Control	39	61	83	29	NA	NA
Johnston, 2013	Control	21	NA	82.5	NA	NA	NA
Jung, 2017	Control	84	52	NA	25.4	90.9	NA
Kaseb, 2013	Control	21	52.6	74.35	26.9	NA	NA
Kasliwal, 2015	Control	21	40.4	80.4	27.8	111.6	NA
Katz, 2012	Control	40	57.4	NA	33.2	93.3	NA
Kocyigit, 2006	Control	22	32.8	NA	24.6	NA	NA
Lamarche, 2004	Control	12	65	70	25.8	NA	NA
Le, 2016	Control	36	NA	33.6	33.6	NA	NA
Lee, 2014	Control	30	NA	73	27	111	NA
Lee, 2017	Control	31	46.3	NA	29.6	NA	NA
Li, 2010	Control	25	47.3	85.5	30.9	NA	NA
Li, 2011	Control	20	58	NA	26	NA	30.6
Liu, 2017	Control	56	26.3	62.3	23	NA	34.4
Ma, 2010	Control	22	58.1	89	32.5	NA	NA
Maranhão, 2011	Control	9	15.4	91.7	34	90.7	NA
Mercanligil, 2007	Control	15	48	74.3	26.1	NA	26.2
Mohan, 2018	Control	140	50.4	67.3	26.2	114.3	NA
Morgan, 2000	Control	9	45	NA	24	NA	NA
Mukuddem-Petersen, 2007	Control	22	45	106	35.1	NA	NA
Njike, 2015	Control	112	55	52.6	NA	NA	NA

Njike, 2015	Control	16	56.5	81.3	34.4	89	38.3
O'Byrne, 1997	Control	13	NA	71	26.4	NA	34.5
Olmedilla-Alonso, 2008	Control	25	54.4	82.1	30	NA	NA
Orem, 2013	Control	21	44.6	81	27.4	NA	NA
Parham, 2014	Control	21	50	NA	30.2	NA	NA
Park, 2017	Control	84	39.44	76.1	27.1	109.9	NA
Pearson, 2016	Control	27	38.6	67.6	23	NA	NA
Pearson, 2016	Control	29	39.8	68.9	23.6	NA	NA
Pearson, 2016	Control	25	37.5	69.7	24.1	NA	NA
Razquin, 2010	Control	196	68.3	74.5	29.2	NA	NA
Ren, 2020	Control	23	70.5	63.1	23.7	NA	NA
Rock, 2017	Control	51	52.2	90.9	32.4	108.6	NA
Rock, 2020	Control	45	55	93.8	32.8	104.7	NA
Ros, 2004	Control	20	55	70.6	NA	NA	NA
Ruisinger, 2015	Control	26	59.3	81.3	28.6	NA	NA
Sabaté, 2003	Control	25	41	71	NA	NA	NA
Sabaté, 2005	Control	41	54.3	78.5	26.9	NA	32.2
Salas-Salvado, 2008	Control	404	67.9	75.4	29.5	NA	NA
Sauder, 2015	Control	30	56	90.6	31.2	NA	NA
Schutte, 2006	Control	21	NA	NA	35.5	103.3	NA
Sheridan, 2007	Control	15	60	79.4	28	NA	NA
Somerset, 2013	Control	64	43.2	99.6	35.8	97.5	NA
Souza, 2018	Control	22	NA	81.9	32.5	87	48.8
Spaccarotella, 2008	Control	21	66	84.8±2.9	NA	NA	NA
Spiller, 1998	Control	15	53	69	NA	NA	NA
Tan, 2013	Control	27	29	77.2	27	94.8	NA
Tapsell, 2004	Control	58	60.5	81.87	29.2	NA	31.2
Tapsell, 2009	Control	17	54	93.4	33	NA	38.1
Tapsell, 2017	Control	61	NA	NA	NA	NA	41.3
Tey, 2013	Control	37	41	88.7	30.4	NA	33.9
Tindall, 2019	Control	36	43	NA	30.3	NA	NA
Wang, 2020	Control	111	46.2	73.6	27.6	NA	NA
Wien, 2003	Control	33	57	114	37	120	NA
Wien, 2010	Control	33	54	80.5	29	96	NA

Wien, 2014	Control	30	64	90.4	33.4	110.9	NA
Williams, 2019	Control	24	NA	NA	NA	NA	NA
Wu, 2010	Control	95	48	70.6	25.4	NA	NA
Yilmaz, 2019	Control	10	52	81.9	36	108.1	43.4
Zambón, 2000	Control	49	56	70.6	27	NA	NA
Zibaeenezzhad, 2019	Control	45	54	73.8	27.2	NA	39.1

Table S7. Direct and indirect evidence and network meta-analysis results summary table for body weight using CINeMA.

Comparison	Evidence	NMA SMD	Direct SMD	Indirect SMD	Difference of SMD (inconsistency)	P value	Incoherence judgment
**An:C**	Mixed	-0.069 (-0.154 to 0.017)	-0.073 (-0.160 to 0.015)	0.027 (-0.397 to 0.451)	-0.100 (-0.533 to 0.333)	0.650	No concerns
**An:Wn**	Mixed	-0.094 (-0.210 to 0.022)	-0.002 (-0.417 to 0.414)	-0.102 (-0.223 to 0.019)	0.100 (-0.333 to 0.533)	0.650	No concerns
**Bn:C**	Direct	-	-0.070 (-1.010 to 0.870)	-	-	-	No concerns
**C:Cs**	Mixed	-0.031 (-0.229 to 0.166)	-0.028 (-0.237 to 0.180)	-0.056 (-0.666 to 0.555)	0.028 (-0.618 to 0.673)	0.933	No concerns
**C:Hn**	Direct	-	-0.068 (-0.251 to 0.115)	-	-	-	No concerns
**C:Md**	Direct	-	0.120 (-0.225 to 0.465)	-	-	-	No concerns
**C:Mx**	Direct	-	-0.021 (-0.068 to 0.026)	-	-	-	No concerns
**C:Pc**	Direct	-	-0.005 (-0.162 to 0.153)	-	-	-	No concerns
**C:Pn**	Direct	-	-0.123 (-0.272 to 0.027)	-	-	-	No concerns
**C:Wn**	Mixed	-0.025 (-0.107 to 0.056)	-0.030 (-0.114 to 0.054)	0.050 (-0.304 to 0.404)	-0.079 (-0.443 to 0.284)	0.669	No concerns
**Cs:Wn**	Mixed	0.006 (-0.204 to 0.216)	0.030 (-0.575 to 0.635)	0.003 (-0.222 to 0.227)	0.028 (-0.618 to 0.673)	0.933	No concerns
**An:Bn**	Indirect	-	-	0.001 (-0.943 to -0.945)	-	-	No concerns
**An:Cs**	Indirect	-	-	-0.100 (-0.315 to 0.115)	-	-	No concerns
**An:Hn**	Indirect	-	-	-0.137 (-0.339 to 0.065)	-	-	No concerns
**An:Md**	Indirect	-	-	0.051 (-0.304 to 0.407)	-	-	No concerns
**An:Mx**	Indirect	-	-	-0.090 (-0.188 to 0.008)	-	-	No concerns
**An:Pc**	Indirect	-	-	-0.073 (-0.253 to 0.106)	-	-	No concerns
**An:Pn**	Indirect	-	-	-0.192 (-0.364 to -0.019)	-	-	No concerns
**Bn:Cs**	Indirect	-	-	-0.101 (-1.062 to 0.859)	-	-	No concerns
**Bn:Hn**	Indirect	-	-	-0.138 (-1.096 to 0.820)	-	-	No concerns
**Bn:Md**	Indirect	-	-	0.050 (-0.951 to 1.051)	-	-	No concerns
**Bn:Mx**	Indirect	-	-	-0.091 (-1.032 to 0.850)	-	-	No concerns
**Bn:Pc**	Indirect	-	-	-0.075 (-1.028 to 0.878)	-	-	No concerns
**Bn:Pn**	Indirect	-	-	-0.193 (-1.145 to 0.759)	-	-	No concerns
**Bn:Wn**	Indirect	-	-	-0.095 (-1.039 to 0.848)	-	-	No concerns

**Cs:Hn**	Indirect	-	-	-0.037 (-0.306 to 0.232)	-	-	No concerns
**Cs:Md**	Indirect	-	-	0.151 (-0.246 to 0.549)	-	-	No concerns
**Cs:Mx**	Indirect	-	-	0.010 (-0.193 to 0.213)	-	-	No concerns
**Cs:Pc**	Indirect	-	-	0.027 (-0.226 to 0.279)	-	-	No concerns
**Cs:Pn**	Indirect	-	-	-0.092 (-0.339 to 0.156)	-	-	No concerns
**Hn:Md**	Indirect	-	-	0.188 (-0.202 to 0.579)	-	-	No concerns
**Hn:Mx**	Indirect	-	-	0.047 (-0.142 to 0.236)	-	-	No concerns
**Hn:Pc**	Indirect	-	-	0.064 (-0.178 to 0.305)	-	-	No concerns
**Hn:Pn**	Indirect	-	-	-0.055 (-0.291 to 0.182)	-	-	No concerns
**Hn:Wn**	Indirect	-	-	0.043 (-0.158 to 0.243)	-	-	No concerns
**Md:Mx**	Indirect	-	-	-0.141 (-0.489 to 0.207)	-	-	No concerns
**Md:Pc**	Indirect	-	-	-0.125 (-0.504 to 0.255)	-	-	No concerns
**Md:Pn**	Indirect	-	-	-0.243 (-0.619 to 0.133)	-	-	No concerns
**Md:Wn**	Indirect	-	-	-0.145 (-0.500 to 0.209)	-	-	No concerns
**Mx:Pc**	Indirect	-	-	0.017 (-0.148 to 0.181)	-	-	No concerns
**Mx:Pn**	Indirect	-	-	-0.102 (-0.258 to 0.055)	-	-	No concerns
**Mx:Wn**	Indirect	-	-	-0.004 (-0.098 to 0.090)	-	-	No concerns
**Pc:Pn**	Indirect	-	-	-0.118 (-0.336 to 0.099)	-	-	No concerns
**Pc:Wn**	Indirect	-	-	-0.021 (-0.198 to 0.157)	-	-	No concerns
**Pn:Wn**	Indirect	-	-	0.098 (-0.073 to 0.268)	-	-	No concerns

SMD: Standardized mean difference; **An**: almond, **Bn**: brazil nut, **C**: control, **Cs**: cashew, **Hn**: Hazelnut, **Mx**: mixed nuts, **Md**: macadamia, **Pa**: pecan, **Pc**: pistachio, **Pn**: peanut; **Wn**: walnut.
$\chi 2$ statistic: 0.214 (2 degrees of freedom), **P value**: 0.898.

Table S8. Direct and indirect evidence and network meta-analysis results summary table for body mass index using CINeMA.

Comparison	Evidence	NMA SMD	Direct SMD	Indirect SMD	Difference of SMD (inconsistency)	P value	Incoherence judgment
**An:C**	Mixed	-0.061 (-0.169 to 0.046)	-0.070 (-0.179 to 0.039)	0.142 (-0.394 to 0.678)	-0.212 (-0.759 to 0.335)	0.447	No concerns
**An:Pn**	Mixed	-0.117 (-0.311 to 0.077)	-0.130 (-0.920 to 0.660)	-0.116 (-0.316 to 0.084)	-0.014 (-0.829 to 0.801)	0.974	No concerns
**An:Wn**	Mixed	-0.098 (-0.241 to 0.045)	0.270 (-0.440 to 0.980)	-0.113 (-0.260 to 0.033)	0.383 (-0.342 to 1.108)	0.300	No concerns
**Bn:C**	Direct	-	-0.131 (-0.698 to 0.437)	-	-	-	No concerns
**C:Cs**	Mixed	-0.001 (-0.199 to 0.197)	-0.002 (-0.211 to 0.207)	0.004 (-0.614 to 0.622)	-0.006 (-0.658 to 0.646)	0.986	No concerns
**C:Hn**	Direct	-	-0.066 (-0.250 to 0.118)	-	-	-	No concerns
**C:Md**	Direct	-	0.070 (-0.275 to 0.415)	-	-	-	No concerns
**C:Mx**	Direct	-	0.050 (-0.096 to 0.196)	-	-	-	No concerns
**C:Pa**	Direct	-	0.000 (-0.900 to 0.900)	-	-	-	No concerns
**C:Pc**	Direct	-	-0.007 (-0.188 to 0.174)	-	-	-	No concerns
**C:Pn**	Mixed	-0.056 (-0.220 to 0.109)	-0.055 (-0.223 to 0.113)	-0.069 (-0.866 to 0.729)	0.014 (-0.801 to 0.829)	0.974	No concerns
**C:Wn**	Mixed	-0.036 (-0.133 to 0.061)	-0.043 (-0.142 to 0.056)	0.129 (-0.351 to 0.608)	-0.172 (-0.662 to 0.308)	0.492	No concerns
**Cs:Wn**	Mixed	-0.035 (-0.251 to 0.181)	-0.040 (-0.650 to 0.570)	-0.034 (-0.265 to 0.197)	-0.006 (-0.658 to 0.646)	0.986	No concerns
**An:Bn**	Indirect	-	-	0.069 (-0.508 to 0.646)	-	-	No concerns
**An:Cs**	Indirect	-	-	-0.063 (-0.288 to 0.162)	-	-	No concerns
**An:Hn**	Indirect	-	-	-0.128 (-0.340 to 0.085)	-	-	No concerns
**An:Md**	Indirect	-	-	0.009 (-0.353 to 0.370)	-	-	No concerns
**An:Mx**	Indirect	-	-	-0.011 (-0.193 to 0.170)	-	-	No concerns
**An:Pa**	Indirect	-	-	-0.061 (-0.968 to 0.845)	-	-	No concerns
**An:Pc**	Indirect	-	-	-0.069 (-0.279 to 0.141)	-	-	No concerns
**Bn:Cs**	Indirect	-	-	-0.132 (-0.733 to 0.469)	-	-	No concerns
**Bn:Hn**	Indirect	-	-	-0.197 (-0.793 to 0.400)	-	-	No concerns
**Bn:Md**	Indirect	-	-	-0.061 (-0.724 to 0.603)	-	-	No concerns
**Bn:Mx**	Indirect	-	-	-0.081 (-0.66 to 0.505)	-	-	No concerns
**Bn:Pa**	Indirect	-	-	-0.131 (-1.194 to 0.933)	-	-	No concerns
**Bn:Pc**	Indirect	-	-	-0.138 (-0.733 to 0.458)	-	-	No concerns

**Bn:Pn**	Indirect	-	-	-0.186 (-0.777 to 0.404)	-	-	No concerns
**Bn:Wn**	Indirect	-	-	-0.167 (-0.742 to 0.409)	-	-	No concerns
**Cs:Hn**	Indirect	-	-	-0.065 (-0.335 to 0.205)	-	-	No concerns
**Cs:Md**	Indirect	-	-	0.071 (-0.327 to 0.469)	-	-	No concerns
**Cs:Mx**	Indirect	-	-	0.051 (-0.195 to 0.297)	-	-	No concerns
**Cs:Pa**	Indirect	-	-	0.001 (-0.920 to 0.923)	-	-	No concerns
**Cs:Pc**	Indirect	-	-	-0.006 (-0.119 to 0.351)	-	-	No concerns
**Cs:Pn**	Indirect	-	-	-0.054 (-0.312 to 0.203)	-	-	No concerns
**Hn:Md**	Indirect	-	-	0.136 (-0.255 to 0.527)	-	-	No concerns
**Hn:Mx**	Indirect	-	-	0.116 (-0.119 to 0.351)	-	-	No concerns
**Hn:Pa**	Indirect	-	-	0.066 (-0.853 to 0.985)	-	-	No concerns
**Hn:Pc**	Indirect	-	-	0.059 (-0.199 to 0.317)	-	-	No concerns
**Hn:Pn,0**	Indirect	-	-	0.011 (-0.236 to 0.257)	-	-	No concerns
**Hn:Wn**	Indirect	-	-	0.030 (-0.178 to 0.238)	-	-	No concerns
**Md:Mx**	Indirect	-	-	-0.020 (-0.395 to 0.355)	-	-	No concerns
**Md:Pa**	Indirect	-	-	-0.070 (-1.034 to 0.894)	-	-	No concerns
**Md:Pc**	Indirect	-	-	-0.077 (-0.467 to 0.312)	-	-	No concerns
**Md:Pn**	Indirect	-	-	-0.126 (-0.508 to 0.257)	-	-	No concerns
**Md:Wn**	Indirect	-	-	-0.106 (-0.465 to 0.252)	-	-	No concerns
**Mx:Pa**	Indirect	-	-	-0.050 (-0.962 to 0.862)	-	-	No concerns
**Mx:Pc**	Indirect	-	-	-0.057 (-0.290 to 0.175)	-	-	No concerns
**Mx:Pn**	Indirect	-	-	-0.106 (-0.326 to 0.114)	-	-	No concerns
**Mx:Wn**	Indirect	-	-	-0.086 (-0.262 to 0.089)	-	-	No concerns
**Pa:Pc**	Indirect	-	-	-0.007 (-0.925 to 0.911)	-	-	No concerns
**Pa:Pn**	Indirect	-	-	-0.056 (-0.971 to 0.859)	-	-	No concerns
**Pa:Wn**	Indirect	-	-	-0.036 (-0.941 to 0.869)	-	-	No concerns
**Pc:Pn**	Indirect	-	-	-0.048 (-0.293 to 0.196)	-	-	No concerns
**Pc:Wn**	Indirect	-	-	-0.029 (-0.234 to 0.176)	-	-	No concerns
**Pn:Wn**	Indirect	-	-	0.019 (-0.172 to 0.210)	-	-	No concerns

SMD: Standardized mean difference; **An**: almond, **Bn**: brazil nut, **C**: control, **Cs**: cashew, **Hn**: Hazelnut, **Mx**: mixed nuts, **Md**: macadamia, **Pa**: pecan, **Pc**: pistachio, **Pn**: peanut; **Wn**: walnut.
$\chi 2$ statistic: 1.075 (3 degrees of freedom), **P value**: 0.783.

Table S9. Direct and indirect evidence and network meta-analysis results summary table for waist circumference using CINeMA.

Comparison	Evidence	NMA SMD	Direct SMD	Indirect SMD	Difference of SMD (inconsistency)	P value	Incoherence judgment
**An:C**	Direct	-	-0.148 (-0.269 to -0.028)	-	-	-	No concerns
**Bn:C**	Direct	-	0.075 (-0.512 to 0.662)	-	-	-	No concerns
**C:Cs**	Mixed	-0.002 (-0.243 to 0.239)	0.007 (-0.252 to 0.266)	-0.057 (-0.712 to 0.597)	0.064 (-0.640 to 0.768)	0.858	No concerns
**C:Hn**	Direct	-	-0.302 (-0.606 to 0.002)	-	-	-	No concerns
**C:Md**	Direct	-	0.070 (-0.332 to 0.472)	-	-	-	No concerns
**C:Mx**	Direct	-	0.126 (-0.013 to 0.265)	-	-	-	No concerns
**C:Pc**	Direct	-	-0.138 (-0.366 to 0.091)	-	-	-	No concerns
**C:Pn**	Direct	-	0.060 (-0.131 to 0.252)	-	-	-	No concerns
**C:Wn**	Mixed	0.004 (-0.112 to 0.121)	0.003 (-0.116 to 0.121)	0.067 (-0.627 to 0.761)	-0.064 (-0.768 to 0.640)	0.858	No concerns
**Cs:Wn**	Mixed	0.006 (-0.254 to 0.267)	0.060 (-0.584 to 0.704)	-0.004 (-0.289 to 0.281)	0.064 (-0.640 to 0.768)	0.858	No concerns
**An:Bn**	Indirect	-	-	-0.224 (-0.823 to 0.376)	-	-	No concerns
**An:Cs**	Indirect	-	-	-0.150 (-0.420 to 0.119)	-	-	No concerns
**An:Hn**	Indirect	-	-	-0.450 (-0.777 to -0.123)	-	-	No concerns
**An:Md**	Indirect	-	-	-0.079 (-0.498 to 0.341)	-	-	No concerns
**An:Mx**	Indirect	-	-	-0.022 (-0.206 to 0.161)	-	-	No concerns
**An:Pc**	Indirect	-	-	-0.286 (-0.545 to -0.028)	-	-	No concerns
**An:Pn**	Indirect	-	-	-0.088 (-0.315 to 0.139)	-	-	No concerns
**An:Wn**	Indirect	-	-	-0.144 (-0.312 to 0.024)	-	-	No concerns
**Bn:Cs**	Indirect	-	-	0.073 (-0.561 to 0.708)	-	-	No concerns
**Bn:Hn**	Indirect	-	-	-0.227 (-0.888 to 0.435)	-	-	No concerns
**Bn:Md**	Indirect	-	-	0.145 (-0.567 to 0.857)	-	-	No concerns
**Bn:Mx**	Indirect	-	-	0.201 (-0.402 to 0.804)	-	-	No concerns
**Bn:Pc**	Indirect	-	-	-0.063 (-0.693 to 0.567)	-	-	No concerns
**Bn:Pn**	Indirect	-	-	0.136 (-0.482 to 0.753)	-	-	No concerns
**Bn:Wn**	Indirect	-	-	0.079 (-0.519 to 0.678)	-	-	No concerns
**Cs:Hn**	Indirect	-	-	-0.300 (-0.688 to 0.088)	-	-	No concerns

**Cs:Md**	Indirect	-	-	0.072 (-0.397 to 0.541)	-	-	No concerns
**Cs:Mx**	Indirect	-	-	0.128 (-0.150 to 0.406)	-	-	No concerns
**Cs:Pc**	Indirect	-	-	-0.136 (-0.468 to 0.196)	-	-	No concerns
**Cs:Pn**	Indirect	-	-	0.062 (-0.246 to 0.370)	-	-	No concerns
**Hn:Md**	Indirect	-	-	0.372 (-0.133 to 0.876)	-	-	No concerns
**Hn:Mx**	Indirect	-	-	0.428 (0.094 to 0.762)	-	-	No concerns
**Hn:Pc**	Indirect	-	-	0.164 (-0.216 to 0.544)	-	-	No concerns
**Hn:Pn**	Indirect	-	-	0.362 (0.003 to 0.722)	-	-	No concerns
**Hn:Wn**	Indirect	-	-	0.306 (-0.019 to 0.632)	-	-	No concerns
**Md:Mx**	Indirect	-	-	0.056 (-0.369 to 0.481)	-	-	No concerns
**Md:Pc**	Indirect	-	-	-0.208 (-0.670 to 0.255)	-	-	No concerns
**Md:Pn**	Indirect	-	-	-0.009 (-0.455 to 0.436)	-	-	No concerns
**Md:Wn**	Indirect	-	-	-0.066 (-0.484 to 0.353)	-	-	No concerns
**Mx:Pc**	Indirect	-	-	-0.264 (-0.531 to 0.003)	-	-	No concerns
**Mx:Pn**	Indirect	-	-	-0.066 (-0.302 to 0.171)	-	-	No concerns
**Mx:Wn**	Indirect	-	-	-0.122 (-0.303 to 0.059)	-	-	No concerns
**Pc:Pn**	Indirect	-	-	0.198 (-0.100 to 0.497)	-	-	No concerns
**Pc:Wn**	Indirect	-	-	0.142 (-0.114 to 0.399)	-	-	No concerns
**Pn:Wn**	Indirect	-	-	-0.056 (-0.281 to 0.168)	-	-	No concerns

SMD: Standardized mean difference; **An**: almond, **Bn**: brazil nut, **C**: control, **Cs**: cashew, **Hn**: Hazelnut, **Mx**: mixed nuts, **Md**: macadamia, **Pa**: pecan, **Pc**: pistachio, **Pn**: peanut; **Wn**: walnut.
χ2 statistic: 0.031 (1 degrees of freedom), **P value**: 0.860.

Table S10. Direct and indirect evidence and network meta-analysis results summary table for body fat percentage using CINeMA.

Comparison	Evidence	NMA SMD	Direct SMD	Indirect SMD	Difference of SMD (inconsistency)	P value	Incoherence judgment
**An:C**	Direct	-	0.040 (-0.135 to 0.215)	-	-	-	No concerns
**Bn:C**	Direct	-	-0.020 (-0.762 to 0.722)	-	-	-	No concerns
**C:Hn**	Direct	-	0.044 (-0.269 to 0.180)	-	-	-	No concerns
**C:Mx**	Direct	-	0.209 (-0.051 to 0.468)	-	-	-	No concerns
**C:Pc**	Direct	-	0.070 (-0.485 to 0.625)	-	-	-	No concerns
**C:Pn**	Direct	-	0.158 (-0.199 to 0.514)	-	-	-	No concerns
**C:Wn**	Direct	-	0.145 (-0.019 to 0.309)	-	-	-	No concerns
**An:Bn**	Indirect	-	-	0.060 (-0.703 to 0.822)	-	-	No concerns
**An:Hn**	Indirect	-	-	-0.005 (-0.289 to 0.280)	-	-	No concerns
**An:Mx**	Indirect	-	-	0.248 (-0.064 to 0.561)	-	-	No concerns
**An:Pc**	Indirect	-	-	0.110 (-0.472 to 0.629)	-	-	No concerns
**An:Pn**	Indirect	-	-	0.198 (-0.200 to 0.595)	-	-	No concerns
**An:Wn**	Indirect	-	-	0.185 (-0.055 to 0.424)	-	-	No concerns
**Bn:Hn**	Indirect	-	-	-0.064 (-0.839 to 0.711)	-	-	No concerns
**Bn:Mx**	Indirect	-	-	0.189 (-0.597 to 0.975)	-	-	No concerns
**Bn:Pc**	Indirect	-	-	0.050 (-0.877 to 0.977)	-	-	No concerns
**Bn:Pn**	Indirect	-	-	0.138 (-0.685 to 0.961)	-	-	No concerns
**Bn:Wn**	Indirect	-	-	0.125 (-0.635 to 0.885)	-	-	No concerns
**Hn:Mx**	Indirect	-	-	0.253 (-0.090 to 0.596)	-	-	No concerns
**Hn:Pc**	Indirect	-	-	0.114 (-0.484 to 0.713)	-	-	No concerns
**Hn:Pn**	Indirect	-	-	0.202 (-0.219 to 0.624)	-	-	No concerns
**Hn:Wn**	Indirect	-	-	0.189 (-0.088 to 0.467)	-	-	No concerns
**Mx:Pc**	Indirect	-	-	-0.139 (-0.751 to 0.474)	-	-	No concerns
**Mx:Pn**	Indirect	-	-	-0.051 (-0.492 to 0.390)	-	-	No concerns
**Mx:Wn**	Indirect	-	-	-0.064 (-0.370 to 0.243)	-	-	No concerns

**Pc:Pn**	Indirect	-	-	0.088 (-0.572 to 0.748)	-	-	No concerns
**Pc:Wn**	Indirect	-	-	0.075 (-0.504 to 0.654)	-	-	No concerns
**Pn:Wn**	Indirect	-	-	-0.013 (-0.405 to 0.379)	-	-	No concerns

SMD: Standardized mean difference; **An**: almond, **Bn**: brazil nut, **C**: control, **Cs**: cashew, **Hn**: Hazelnut, **Mx**: mixed nuts, **Md**: macadamia, **Pa**: pecan, **Pc**: pistachio, **Pn**: peanut; **Wn**: walnut.
χ2 statistic: 0.000 (0 degrees of freedom), **P value**: NA

Table S11. Quality Assessment (RoB 2.0).

Study	Experimental	Comparator	Outcome	Weight	D1	D2	D3	D4	D5	Overall
RCTs with Intention-to-treat analyses										
Abazarfard, 2014	NA	NA	NA	1	+	+	+	+	+	+
Abbaspour, 2019	NA	NA	NA	1	!	+	+	+	+	!
Abdrabalnabi, 2020	NA	NA	NA	1	+	-	+	+	+	-
Alves, 2014	NA	NA	NA	1	!	+	+	+	+	!
Barbour, 2015	NA	NA	NA	1	+	+	+	+	+	+
Bashan 2018	NA	NA	NA	1	+	!	+	+	+	!
Bento, 2014	NA	NA	NA	1	+	+	!	+	+	!
Bowen, 2019	NA	NA	NA	1	+	+	+	+	+	+
Canales, 2007	NA	NA	NA	1	+	+	+	+	+	+
Carughi, 2019	NA	NA	NA	1	+	+	+	+	+	+
Casas-Agustench, 2011	NA	NA	NA	1	+	+	+	+	+	+
Chiang, 2012	NA	NA	NA	1	+	+	+	+	+	+
Chisholm, 2005	NA	NA	NA	1	+	+	+	-	+	-
Claesson, 2009	NA	NA	NA	1	+	+	+	-	+	-

Study	Experimental	Comparator	Outcome	Weight	D1	D2	D3	D4	D5	Overall
Damasceno, 2013	NA	NA	NA	1	+	+	+	!	+	!
Damavandi, 2013	NA	NA	NA	1	!	+	+	+	+	!
Dhillon, 2016	NA	NA	NA	1	+	+	+	+	+	+
Dhillon, 2018	NA	NA	NA	1	+	+	+	+	+	+
Dikariyanto, 2020	NA	NA	NA	1	+	+	+	+	+	+
Estruch, 2016	NA	NA	NA	1	+	!	+	+	+	!
Fatahi, 2019	NA	NA	NA	1	+	+	+	+	+	+
Foster, 2012	NA	NA	NA	1	+	!	+	!	+	!
Gebauer, 2008	NA	NA	NA	1	+	+	+	!	+	!
Gozde, 2019	NA	NA	NA	1	+	+	+	+	+	+
Gulati, 2014	NA	NA	NA	1	+	+	+	+	+	+
Hernández-Alonso, 2014	NA	NA	NA	1	+	+	+	+	+	+
Jenkins, 1997	NA	NA	NA	1	!	+	+	!	!	!
Johnston, 2013	NA	NA	NA	1	+	!	+	-	+	-
Katz, 2012	NA	NA	NA	1	+	+	+	+	+	+
Kocyigit, 2006	NA	NA	NA	1	+	+	+	+	+	+

Study	Experimental	Comparator	Outcome	Weight	D1	D2	D3	D4	D5	Overall
Lamarche, 2004	NA	NA	NA	1	!	+	+	+	-	-
Lee, 2014	NA	NA	NA	1	!	+	+	+	+	!
Ma, 2010	NA	NA	NA	1	+	+	!	+	+	!
Maranhao, 2011	NA	NA	NA	1	+	+	+	-	+	-
Mercanligil, 2007	NA	NA	NA	1	!	!	+	-	+	-
Morgan, 2000	NA	NA	NA	1	+	+	+	+	+	+
Mukuddem-Petersen, 2007	NA	NA	NA	1	+	+	+	+	+	+
Nijke, 2015 (a)	NA	NA	NA	1	+	+	+	+	+	+
Nijke, 2015 (b)	NA	NA	NA	1	+	+	+	+	+	+
Olmedilla-Alonso, 2008	NA	NA	NA	1	!	!	+	+	+	!
Orem, 2013	NA	NA	NA	1	-	+	+	+	+	-
Parham, 2014	NA	NA	NA	1	+	+	+	+	+	+
Razquin, 2010	NA	NA	NA	1	+	+	+	+	+	+
Rock, 2017	NA	NA	NA	1	+	+	+	+	+	+
Sabaté, 2005	NA	NA	NA	1	+	+	+	!	+	!
Salas-Salvadó, 2008	NA	NA	NA	1	+	!	!	!	+	!

Study	Experimental	Comparator	Outcome	Weight	D1	D2	D3	D4	D5	Overall
Schutte, 2006	NA	NA	NA	1	+	+	+	+	+	+
Somerset, 2013	NA	NA	NA	1	!	+	+	+	+	!
Spaccarotella, 2008	NA	NA	NA	1	!	+	+	+	+	!
Tapsell, 2004	NA	NA	NA	1	+	+	+	+	+	+
Tapsell, 2017	NA	NA	NA	1	+	+	+	+	+	+
Tey, 2013	NA	NA	NA	1	+	+	+	+	+	+
Wang, 2020	NA	NA	NA	1	+	+	+	!	+	!
Wien, 2003	NA	NA	NA	1	+	!	+	+	+	!
Wien, 2010	NA	NA	NA	1	+	!	+	!	+	!
Wien, 2014	NA	NA	NA	1	+	!	+	!	+	!
Zibaeenezzhad, 2019	NA	NA	NA	1	!	+	+	!	+	!

RCTs with per-protocol analyses

Álvarez-Pérez, 2016	NA	NA	NA	1	+	+	+	-	+	-
Berryman, 2015	NA	NA	NA	1	+	+	+	+	+	+
Bitok, 2018	NA	NA	NA	1	+	-	+	+	+	-
Bulló, 2009	NA	NA	NA	1	+	!	+	+	+	!

Author, year	Experimental	Comparator	Outcome	Weight	D1	D2	D3	D4	D5	Overall
Caldas, 2020	NA	NA	NA	1	+	!	!	+	+	!
Chen, 2017	NA	NA	NA	1	+	!	+	+	+	!
Coates, 2020	NA	NA	NA	1	+	!	!	+	+	!
Costa e Silva, 2019	NA	NA	NA	1	+	!	!	+	+	!
Damasceno, 2011	NA	NA	NA	1	+	!	+	!	+	!
Damavandi, 2019	NA	NA	NA	1	!	+	+	!	+	!
Domenech, 2019	NA	NA	NA	1	+	!	+	+	+	!
Fantino, 2019	NA	NA	NA	1	+	+	+	!	+	!
Ghadiminouran, 2009	NA	NA	NA	1	+	+	!	!	+	!
Hollis, 2007	NA	NA	NA	1	+	!	-	-	+	-
Hou, 2018	NA	NA	NA	1	+	!	-	+	+	-
Hwang, 2019	NA	NA	NA	1	+	!	-	+	+	-
Jamshed, 2015	NA	NA	NA	1	+	!	!	+	+	!
Jenkins, 2002	NA	NA	NA	1	!	-	+	+	+	-
Jung, 2017	NA	NA	NA	1	!	-	+	+	+	-
Kalgaonkar, 2010	NA	NA	NA	1	!	+	!	+	+	!

Author, year	Experimental	Comparator	Outcome	Weight	D1	D2	D3	D4	D5	Overall
Kaseb, 2013	NA	NA	NA	1	!	-	-	+	+	-
Kasliwal, 2015	NA	NA	NA	1	!	+	+	!	+	!
Le, 2016	NA	NA	NA	1	+	-	!	-	+	-
Lee, 2017	NA	NA	NA	1	+	!	!	+	+	!
Li, 2010	NA	NA	NA	1	!	!	+	!	+	!
Li, 2011	NA	NA	NA	1	+	-	-	+	+	-
Liu, 2017	NA	NA	NA	1	+	-	-	+	+	-
Mohan, 2018	NA	NA	NA	1	+	!	!	+	+	!
O'Byrne, 1997	NA	NA	NA	1	!	-	!	+	+	-
Park, 2017	NA	NA	NA	1	+	-	!	+	+	-
Pearson, 2016	NA	NA	NA	1	+	!	!	+	+	!
Ren, 2020	NA	NA	NA	1	+	!	!	+	+	!
Rock, 2020	NA	NA	NA	1	+	-	+	!	+	-
Ros, 2004	NA	NA	NA	1	!	+	+	!	+	!
Ruisinger, 2015	NA	NA	NA	1	!	+	+	+	+	!
Sabaté, 2003	NA	NA	NA	1	!	+	+	+	+	!

Sauder, 2015	NA	NA	NA	1	+	!	+	+	+	!
Sheridan, 2007	NA	NA	NA	1	!	!	+	+	+	!
Souza, 2018	NA	NA	NA	1	+	-	!	+	+	-
Spiller, 1998	NA	NA	NA	1	!	!	!	!	+	-
Tan, 2013	NA	NA	NA	1	+	!	!	!	+	!
Tapsell, 2009	NA	NA	NA	1	+	-	!	+	+	-
Tindall, 2019	NA	NA	NA	1	+	!	!	+	+	!
Williams, 2019	NA	NA	NA	1	+	!	-	!	+	-
Wu, 2010	NA	NA	NA	1	+	+	!	+	+	!
Zambón, 2000	NA	NA	NA	1	+	!	+	!	+	!
Yilmaz, 2019	NA	NA	NA	1	!	!	+	+	+	!

D1: Randomisation process; D2: Deviations from the intended interventions; D3: Missing outcome data; D4: Measurement of the outcome; D5: Selection of the reported results.

+ : Low risk of bias; ! : Some concerns; - : High risk of bias.

Table S12. Percentage of change for healthy/normal weight and people with Overweight/obesity.

Population	Outcome	Group	n (groups/studies)	% of change	LL	UL	I2 (% heterogeneity)	p-value (heterogeneity)
Healthy	BW	Nuts	14	-0.506	-1.043	0.032	0	0.995
Healthy	BW	Control	12	-1.025	-2.146	0.096	0	0.978
OW/Ob	BW	Nuts	17	**-3.982**	-6.248	-1.716	85.4	0
OW/Ob	BW	Control	16	**-3.373**	-5.788	-0.958	83.4	0
Healthy	BMI	Nuts	7	0.09	-0.379	0.559	62.5	0.01
Healthy	BMI	Control	5	0.291	-0.282	0.863	21.9	0.275
OW/Ob	BMI	Nuts	16	**-3.294**	-6.206	-0.382	98.9	0
OW/Ob	BMI	Control	15	-2.226	-4.551	0.099	97.8	0
Healthy	WC	Nuts	4	-0.049	-1.573	1.475	0	0.882
Healthy	WC	Control	4	0.158	-1.79	2.105	0	0.984
OW/Ob	WC	Nuts	12	**-4.104**	-7.272	-0.936	92.9	0
OW/Ob	WC	Control	12	**-2.109**	-4.058	-0.16	79.8	0
Healthy	BF%	Nuts	6	0.537	-1.847	2.921	74.2	0.002
Healthy	BF%	Control	6	1.162	-0.717	3.042	46.6	0.095
OW/Ob	BF%	Nuts	6	-1.628	-5.835	2.58	95.4	0

OW/Ob	BF%	Control	5	-0.608	-1.469	0.254	45.4	0.12

Population	Outcome	Group	Length	n (groups/studies)	% of change	LL	UL	I2 (% heterogeneity)	p-value (heterogeneity)
Healthy	BW	Nuts	<12weeks	10	-0.432	-0.988	0.124	0	1
Healthy	BW	Nuts	≥12weeks	4	-1.55	-3.646	0.545	0	0.606
OW/Ob	BW	Nuts	<12weeks	4	0.095	-2.193	2.383	0	0.955
OW/Ob	BW	Nuts	≥12weeks	13	**-4.938**	-7.299	-2.578	84.6	0
Healthy	BMI	Nuts	<12weeks	5	-0.08	-0.511	0.351	11.3	0.341
Healthy	BMI	Nuts	≥12weeks	2	0.008	-1.026	1.042	59.8	0.115
OW/Ob	BMI	Nuts	<12weeks	5	-0.799	-2.058	0.461	66.4	0.02
OW/Ob	BMI	Nuts	≥12weeks	11	**-4.342**	-8.132	-0.552	99.1	0
Healthy	WC	Nuts	<12weeks	3	-0.17	-1.94	1.599	0	0.744
Healthy	WC	Nuts	≥12weeks	1	0.3	-2.699	3.299	0	-
OW/Ob	WC	Nuts	<12weeks	3	-0.672	-2.631	1.287	61.1	0.07
OW/Ob	WC	Nuts	≥12weeks	9	**-4.87**	-8.976	-0.772	90.9	0
Healthy	BF%	Nuts	<12weeks	4	0.282	-1.269	1.833	0	0.82
Healthy	BF%	Nuts	≥12weeks	2	0.256	-6.123	6.634	93.8	0
OW/Ob	BF%	Nuts	<12weeks	2	-0.906	-3.452	1.64	0	0.349

OW/Ob	BF%	Nuts	≥12weeks	4	-2.255	-7.523	3.013	96.6	0

BF%: Body fat percentage; **BMI**: Body mass index; **BW**: body weight; **LL**: lower limit; **UL**: upper limit; OW/**Ob**: overweight/obese; **WC**: waist circumference. **Bold** % of change means significant.

Table S13. GRADE approach: summary of findings.

Certainty assessment							№ of patients		Effect		Certainty	Importance
№ of studies	Study design	Risk of bias	Inconsistency	Indirectness	Imprecision	Other considerations	nut consumption	habitual diets, low-fat diets or isocaloric snacks	Relative (95% CI)	Absolute (95% CI)		
**Body weight (kg) (follow up: range 2 weeks to 240 weeks; Scale from: 61.44 to 107)**												
103	randomised trials	serious [a]	serious [b]	serious [c]	not serious	none	6825	6599	-	SMD **0.01 SD higher** (-0.02 lower to 0.05 higher)	⊕◯◯◯ VERY LOW	NOT IMPORTANT
**Body Mass Index (kg/m2) (follow up: range 3 weeks to 96 weeks; assessed with: calculated; Scale from: 23 to 39)**												
72	randomised trials	serious [d]	serious [b]	serious [c]	not serious	none	2924	2781	-	SMD **0.002 SD lower** (-0.05 lower to 0.05 higher)	⊕◯◯◯ VERY LOW	IMPORTANT
**Waist circumference (cm) (follow up: range 3 weeks to 240 weeks; Scale from: 73.9 to 120)**												
60	randomised trials	serious [e]	serious [b]	serious [c]	not serious	none	5150	5062	-	SMD **0.05 SD lower** (-0.11 lower to 0.01 higher)	⊕◯◯◯ VERY LOW	IMPORTANT

Certainty assessment							№ of patients		Effect		Certainty	Importance
**№ of studies**	**Study design**	**Risk of bias**	**Inconsistency**	**Indirectness**	**Imprecision**	**Other considerations**	**nut consumption**	**habitual diets, low-fat diets or isocaloric snacks**	**Relative (95% CI)**	**Absolute (95% CI)**		
**Body fat (%) (follow up: range 2 weeks to 96 weeks; Scale from: 22.6 to 49.1)**												
33	randomised trials	serious [f]	not serious [g]	serious [c]	not serious	none	760	701	-	SMD **0.07 SD lower** (-0.15 lower to 0.02 higher)	⊕⊕ LOW	IMPORTANT

CI: Confidence interval; **SMD:** Standardised mean difference

Explanations

a. Among the included studies for body weight outcome, there were 54% assessed with some concerns and 21% with high risk of bias by RoB2 Cochrane tool (overall bias). Mostly because of deviations from intended interventions, randomization process and missing outcome data items.

b. The value of the I2 was related with moderate or substantial heterogeneity.

c. Most comparisons were assessed as high indirectness.

d. Among the included studies for BMI outcome, there were 56% assessed with some concerns and 22% with high risk of bias by RoB2 Cochrane tool (overall bias). Mostly because of deviations from intended interventions, randomization process and missing outcome data.

e. Among the included studies for WC outcome, there were 48% assessed with some concerns and 18% with high risk of bias by RoB2 Cochrane tool (overall bias). Mostly because of deviations from intended interventions, randomization process and missing outcome data.

f. Among the included studies for BF% outcome, there were 33% assessed with some concerns and 33% with high risk of bias by RoB2 Cochrane tool (overall bias).

g. There were low heterogeneity (15.5%) and sensitivity analysis showed no significant changes in the effect size estimates.

Table S14. CINeMA final report.

Comparison	Nº studies	Within-study bias	Reporting bias	Indirectness	Imprecision	Heterogeneity	Incoherence	Confidence
**Body weight**								
An:C	30	Some concerns	Undetected	Some concerns	No concerns	No concerns	No concerns	Low
An:Wn	3	Some concerns	Undetected	Some concerns	Some concerns	No concerns	No concerns	Very low
Bn:C	1	Major concerns	Undetected	Some concerns	Major concerns	No concerns	No concerns	Very low
C:Cs	3	Some concerns	Undetected	No concerns	Some concerns	No concerns	No concerns	Low
C:Hn	9	Some concerns	Undetected	Some concerns	Some concerns	No concerns	No concerns	Very low
C:Md	1	Some concerns	Undetected	No concerns	Major concerns	No concerns	No concerns	Very low
C:Mx	12	Some concerns	Undetected	No concerns	No concerns	No concerns	No concerns	Moderate
C:Pc	10	Some concerns	Undetected	Some concerns	No concerns	No concerns	No concerns	Low
C:Pn	9	Some concerns	Undetected	Some concerns	Some concerns	No concerns	No concerns	Very low
C:Wn	24	Some concerns	Undetected	Some concerns	No concerns	No concerns	No concerns	Low
Cs:Wn	1	Some concerns	Undetected	No concerns	Major concerns	No concerns	No concerns	Very low
An:Bn	0	Major concerns	Undetected	Some concerns	Major concerns	No concerns	No concerns	Very low

An:Cs	0	Some concerns	Undetected	No concerns	Some concerns	No concerns	No concerns	Low
An:Hn	0	Some concerns	Undetected	Some concerns	Some concerns	No concerns	No concerns	Very low
An:Md	0	Some concerns	Undetected	No concerns	Major concerns	No concerns	No concerns	Very low
An:Mx	0	Some concerns	Undetected	No concerns	No concerns	No concerns	No concerns	Moderate
An:Pc	0	Some concerns	Undetected	Some concerns	Some concerns	No concerns	No concerns	Very low
An:Pn	0	Some concerns	Undetected	Some concerns	No concerns	No concerns	No concerns	Low
Bn:Cs	0	Some concerns	Undetected	Some concerns	Major concerns	No concerns	No concerns	Very low
Bn:Hn	0	Some concerns	Undetected	Some concerns	Major concerns	No concerns	No concerns	Very low
Bn:Md	0	Major concerns	Undetected	Some concerns	Major concerns	No concerns	No concerns	Very low
Bn:Mx	0	Some concerns	Undetected	Some concerns	Major concerns	No concerns	No concerns	Very low
Bn:Pc	0	Some concerns	Undetected	Some concerns	Major concerns	No concerns	No concerns	Very low
Bn:Pn	0	Some concerns	Undetected	Some concerns	Major concerns	No concerns	No concerns	Very low
Bn:Wn	0	Some concerns	Undetected	Some concerns	Major concerns	No concerns	No concerns	Very low
Cs:Hn	0	Some concerns	Undetected	No concerns	Major concerns	No concerns	No concerns	Very low
Cs:Md	0	Some concerns	Undetected	No concerns	Major concerns	No concerns	No concerns	Very low
Cs:Mx	0	Some concerns	Undetected	No concerns	Some concerns	No concerns	No concerns	Low
Cs:Pc	0	Some concerns	Undetected	No concerns	Major concerns	No concerns	No concerns	Very low
Cs:Pn	0	Some concerns	Undetected	No concerns	Some concerns	No concerns	No concerns	Low
Hn:Md	0	Some concerns	Undetected	No concerns	Major concerns	No concerns	No concerns	Very low
Hn:Mx	0	Some concerns	Undetected	No concerns	Some concerns	No concerns	No concerns	Low
Hn:Pc	0	Some concerns	Undetected	Some concerns	Some concerns	No concerns	No concerns	Very low
Hn:Pn	0	Some concerns	Undetected	Some concerns	Some concerns	No concerns	No concerns	Very low
Hn:Wn	0	Some concerns	Undetected	Some concerns	Some concerns	No concerns	No concerns	Very low
Md:Mx	0	Some concerns	Undetected	No concerns	Major concerns	No concerns	No concerns	Very low
Md:Pc	0	Some concerns	Undetected	No concerns	Major concerns	No concerns	No concerns	Very low
Md:Pn	0	Some concerns	Undetected	No concerns	Some concerns	No concerns	No concerns	Low
Md:Wn	0	Some concerns	Undetected	No concerns	Major concerns	No concerns	No concerns	Very low
Mx:Pc	0	Some concerns	Undetected	Some concerns	No concerns	No concerns	No concerns	Low

Mx:Pn	0	Some concerns	Undetected	No concerns	Some concerns	No concerns	No concerns	Low
Mx:Wn	0	Some concerns	Undetected	Some concerns	No concerns	No concerns	No concerns	Low
Pc:Pn	0	Some concerns	Undetected	Some concerns	Some concerns	No concerns	No concerns	Very low
Pc:Wn	0	Some concerns	Undetected	Some concerns	No concerns	Some concerns	No concerns	Very low
Pn:Wn	0	Some concerns	Undetected	Some concerns	Some concerns	No concerns	No concerns	Very low
**Body mass index**								
An:C	18	Some concerns	Undetected	Some concerns	No concerns	No concerns	No concerns	Low
An:Pn	1	Some concerns	Undetected	Some concerns	Some concerns	No concerns	No concerns	Very low
An:Wn	1	Some concerns	Undetected	Some concerns	Some concerns	No concerns	No concerns	Very low
Bn:C	2	Some concerns	Undetected	No concerns	Major concerns	No concerns	No concerns	Very low
C:Cs	3	No concerns	Undetected	Major concerns	No concerns	Major concerns	No concerns	Very low
C:Hn	9	Some concerns	Undetected	Some concerns	Some concerns	No concerns	No concerns	Very low
C:Md	1	Major concerns	Undetected	No concerns	Major concerns	No concerns	No concerns	Very low
C:Mx	8	Some concerns	Undetected	Some concerns	No concerns	No concerns	No concerns	Low
C:Pa	1	No concerns	Undetected	No concerns	Major concerns	No concerns	No concerns	Low
C:Pc	8	Some concerns	Undetected	Some concerns	No concerns	No concerns	No concerns	Low
C:Pn	7	Some concerns	Undetected	Some concerns	No concerns	Some concerns	No concerns	Very low
C:Wn	12	Major concerns	Undetected	Major concerns	No concerns	No concerns	No concerns	Very low
Cs:Wn	1	Some concerns	Undetected	Major concerns	Some concerns	No concerns	No concerns	Low
An:Bn	0	Some concerns	Undetected	No concerns	Major concerns	No concerns	No concerns	Low
An:Cs	0	No concerns	Undetected	Some concerns	Some concerns	No concerns	No concerns	Low
An:Hn	0	Some concerns	Undetected	Some concerns	Some concerns	No concerns	No concerns	Very low
An:Md	0	Some concerns	Undetected	No concerns	Major concerns	No concerns	No concerns	Very low
An:Mx	0	Some concerns	Undetected	Some concerns	No concerns	No concerns	No concerns	Low
An:Pa	0	No concerns	Undetected	No concerns	Major concerns	No concerns	No concerns	Low
An:Pc	0	Some concerns	Undetected	Some concerns	Some concerns	No concerns	No concerns	Very low
Bn:Cs	0	Some concerns	Undetected	Some concerns	Major concerns	No concerns	No concerns	Very low
Bn:Hn	0	Some concerns	Undetected	No concerns	Major concerns	No concerns	No concerns	Very low

Bn:Md	0	Major concerns	Undetected	No concerns	Major concerns	No concerns	No concerns	Very low
Bn:Mx	0	Some concerns	Undetected	No concerns	Major concerns	No concerns	No concerns	Very low
Bn:Pa	0	Some concerns	Undetected	No concerns	Major concerns	No concerns	No concerns	Very low
Bn:Pc	0	Some concerns	Undetected	No concerns	Major concerns	No concerns	No concerns	Very low
Bn:Pn	0	Some concerns	Undetected	No concerns	Major concerns	No concerns	No concerns	Very low
Bn:Wn	0	Some concerns	Undetected	Some concerns	Major concerns	No concerns	No concerns	Very low
Cs:Hn	0	No concerns	Undetected	Some concerns	Major concerns	No concerns	No concerns	Very low
Cs:Md	0	Some concerns	Undetected	Some concerns	Major concerns	No concerns	No concerns	Very low
Cs:Mx	0	Some concerns	Undetected	Some concerns	Some concerns	Some concerns	No concerns	Very low
Cs:Pa	0	No concerns	Undetected	Some concerns	Major concerns	No concerns	No concerns	Very low
Cs:Pc	0	Some concerns	Undetected	Some concerns	Major concerns	No concerns	No concerns	Very low
Cs:Pn	0	Some concerns	Undetected	Some concerns	Major concerns	No concerns	No concerns	Very low
Hn:Md	0	Some concerns	Undetected	No concerns	Major concerns	No concerns	No concerns	Very low
Hn:Mx	0	Some concerns	Undetected	Some concerns	Some concerns	No concerns	No concerns	Very low
Hn:Pa	0	No concerns	Undetected	No concerns	Major concerns	No concerns	No concerns	Low
Hn:Pc	0	Some concerns	Undetected	Some concerns	Some concerns	Some concerns	No concerns	Very low
Hn:Pn,0	0	Some concerns	Undetected	Some concerns	Major concerns	No concerns	No concerns	Very low
Hn:Wn	0	Some concerns	Undetected	Some concerns	Some concerns	No concerns	No concerns	Very low
Md:Mx	0	Major concerns	Undetected	No concerns	Major concerns	No concerns	No concerns	Very low
Md:Pa	0	Some concerns	Undetected	No concerns	Major concerns	No concerns	No concerns	Very low
Md:Pc	0	Major concerns	Undetected	No concerns	Major concerns	No concerns	No concerns	Very low
Md:Pn	0	Some concerns	Undetected	No concerns	Major concerns	No concerns	No concerns	Very low
Md:Wn	0	Major concerns	Undetected	Some concerns	Major concerns	No concerns	No concerns	Very low
Mx:Pa	0	Some concerns	Undetected	No concerns	Major concerns	No concerns	No concerns	Very low
Mx:Pc	0	Some concerns	Undetected	Some concerns	Some concerns	No concerns	No concerns	Very low
Mx:Pn	0	Some concerns	Undetected	Some concerns	Some concerns	No concerns	No concerns	Very low
Mx:Wn	0	Some concerns	Undetected	Some concerns	Some concerns	No concerns	No concerns	Very low
Pa:Pc	0	Some concerns	Undetected	No concerns	Major concerns	No concerns	No concerns	Very low

Pa:Pn	0	No concerns	Undetected	No concerns	Major concerns	No concerns	No concerns	Low
Pa:Wn	0	Some concerns	Undetected	Some concerns	Major concerns	No concerns	No concerns	Very low
Pc:Pn	0	Some concerns	Undetected	Some concerns	Some concerns	Some concerns	No concerns	Very low
Pc:Wn	0	Some concerns	Undetected	Some concerns	Some concerns	No concerns	No concerns	Very low
Pn:Wn	0	Some concerns	Undetected	Some concerns	Some concerns	No concerns	No concerns	Very low
**Waist circumference**								
An:C	17	Some concerns	Undetected	Some concerns	No concerns	Some concerns	No concerns	Very low
Bn:C	2	Major concerns	Undetected	No concerns	Major concerns	No concerns	No concerns	Very low
C:Cs	3	Some concerns	Undetected	Major concerns	Major concerns	No concerns	No concerns	Very low
C:Hn	4	Some concerns	Undetected	Some concerns	Some concerns	No concerns	No concerns	Very low
C:Md	1	Some concerns	Undetected	No concerns	Major concerns	No concerns	No concerns	Very low
C:Mx	8	Some concerns	Undetected	Some concerns	Some concerns	No concerns	No concerns	Very low
C:Pc	5	Some concerns	Undetected	No concerns	Some concerns	No concerns	No concerns	Low
C:Pn	7	Some concerns	Undetected	Some concerns	Some concerns	Some concerns	No concerns	Very low
C:Wn	12	Some concerns	Undetected	Some concerns	No concerns	Major concerns	No concerns	Very low
Cs:Wn	1	Some concerns	Undetected	Some concerns	Major concerns	No concerns	No concerns	Very low
An:Bn	0	Some concerns	Undetected	No concerns	Major concerns	No concerns	No concerns	Very low
An:Cs	0	Some concerns	Undetected	Some concerns	Some concerns	No concerns	No concerns	Very low
An:Hn	0	Some concerns	Undetected	Some concerns	No concerns	No concerns	No concerns	Low
An:Md	0	Some concerns	Undetected	No concerns	Major concerns	No concerns	No concerns	Very low
An:Mx	0	Some concerns	Undetected	Some concerns	Some concerns	Some concerns	No concerns	Very low
An:Pc	0	Some concerns	Undetected	No concerns	No concerns	Some concerns	No concerns	Low
An:Pn	0	Some concerns	Undetected	Some concerns	Some concerns	Some concerns	No concerns	Very low
An:Wn	0	Some concerns	Undetected	Some concerns	Some concerns	No concerns	No concerns	Very low
Bn:Cs	0	Some concerns	Undetected	Some concerns	Major concerns	No concerns	No concerns	Very low
Bn:Hn	0	Some concerns	Undetected	No concerns	Major concerns	No concerns	No concerns	Very low
Bn:Md	0	Major concerns	Undetected	No concerns	Major concerns	No concerns	No concerns	Very low
Bn:Mx	0	Some concerns	Undetected	No concerns	Major concerns	No concerns	No concerns	Very low

Bn:Pc	0	Some concerns	Undetected	No concerns	Major concerns	No concerns	No concerns	Very low
Bn:Pn	0	Major concerns	Undetected	No concerns	Major concerns	No concerns	No concerns	Very low
Bn:Wn	0	Some concerns	Undetected	No concerns	Major concerns	No concerns	No concerns	Very low
Cs:Hn	0	Some concerns	Undetected	Some concerns	Some concerns	No concerns	No concerns	Very low
Cs:Md	0	Some concerns	Undetected	Some concerns	Major concerns	No concerns	No concerns	Very low
Cs:Mx	0	Some concerns	Undetected	Some concerns	Some concerns	Some concerns	No concerns	Very low
Cs:Pc	0	Some concerns	Undetected	Some concerns	Some concerns	Some concerns	No concerns	Very low
Cs:Pn	0	Some concerns	Undetected	Some concerns	Major concerns	No concerns	No concerns	Very low
Hn:Md,0	0	Some concerns	Undetected	No concerns	Some concerns	No concerns	No concerns	Low
Hn:Mx	0	Some concerns	Undetected	Some concerns	No concerns	No concerns	No concerns	Low
Hn:Pc	0	Some concerns	Undetected	No concerns	Major concerns	No concerns	No concerns	Very low
Hn:Pn	0	Some concerns	Undetected	Some concerns	No concerns	Some concerns	No concerns	Very low
Hn:Wn	0	Some concerns	Undetected	Some concerns	Some concerns	No concerns	No concerns	Very low
Md:Mx	0	Some concerns	Undetected	No concerns	Major concerns	No concerns	No concerns	Very low
Md:Pc	0	Some concerns	Undetected	No concerns	Major concerns	No concerns	No concerns	Very low
Md:Pn	0	Some concerns	Undetected	No concerns	Major concerns	No concerns	No concerns	Very low
Md:Wn	0	Some concerns	Undetected	No concerns	Major concerns	No concerns	No concerns	Very low
Mx:Pc	0	Some concerns	Undetected	Some concerns	Some concerns	No concerns	No concerns	Very low
Mx:Pn	0	Some concerns	Undetected	Some concerns	Some concerns	Some concerns	No concerns	Very low
Mx:Wn	0	Some concerns	Undetected	Some concerns	Some concerns	No concerns	No concerns	Very low
Pc:Pn	0	Some concerns	Undetected	Some concerns	Some concerns	No concerns	No concerns	Very low
Pc:Wn	0	Some concerns	Undetected	No concerns	Some concerns	No concerns	No concerns	Low
Pn:Wn	0	Some concerns	Undetected	Some concerns	Some concerns	Some concerns	No concerns	Very low
**Body fat percentage**								
An:C	9	Some concerns	Undetected	No concerns	Some concerns	Some concerns	Major concerns	Very low
Bn:C	1	Major concerns	Undetected	No concerns	Major concerns	No concerns	Major concerns	Very low
C:Hn	7	Some concerns	Undetected	No concerns	Some concerns	Some concerns	Major concerns	Very low
C:Mx	4	Some concerns	Undetected	Some concerns	Some concerns	No concerns	Major concerns	Very low

C:Pc	1	Major concerns	Undetected	No concerns	Major concerns	No concerns	Major concerns	Very low
C:Pn	4	Some concerns	Undetected	No concerns	Some concerns	Some concerns	Major concerns	Very low
C:Wn	7	Some concerns	Undetected	Some concerns	Some concerns	No concerns	Major concerns	Very low
An:Bn	0	Major concerns	Undetected	No concerns	Major concerns	No concerns	Major concerns	Very low
An:Hn	0	Some concerns	Undetected	No concerns	Major concerns	No concerns	Major concerns	Very low
An:Mx	0	Some concerns	Undetected	Some concerns	Some concerns	No concerns	Major concerns	Very low
An:Pc	0	Major concerns	Undetected	No concerns	Major concerns	No concerns	Major concerns	Very low
An:Pn	0	Some concerns	Undetected	No concerns	Major concerns	No concerns	Major concerns	Very low
An:Wn	0	Some concerns	Undetected	Some concerns	Some concerns	No concerns	Major concerns	Very low
Bn:Hn	0	Major concerns	Undetected	No concerns	Major concerns	No concerns	Major concerns	Very low
Bn:Mx	0	Major concerns	Undetected	Some concerns	Major concerns	No concerns	Major concerns	Very low
Bn:Pc	0	Major concerns	Undetected	No concerns	Major concerns	No concerns	Major concerns	Very low
Bn:Pn	0	Some concerns	Undetected	No concerns	Major concerns	No concerns	Major concerns	Very low
Bn:Wn	0	Some concerns	Undetected	No concerns	Major concerns	No concerns	Major concerns	Very low
Hn:Mx	0	Some concerns	Undetected	Some concerns	Some concerns	No concerns	Major concerns	Very low
Hn:Pc	0	Major concerns	Undetected	No concerns	Major concerns	No concerns	Major concerns	Very low
Hn:Pn	0	Some concerns	Undetected	No concerns	Major concerns	No concerns	Major concerns	Very low
Hn:Wn	0	Some concerns	Undetected	No concerns	Some concerns	No concerns	Major concerns	Very low
Mx:Pc	0	Major concerns	Undetected	Some concerns	Major concerns	No concerns	Major concerns	Very low
Mx:Pn	0	Some concerns	Undetected	Some concerns	Major concerns	No concerns	Major concerns	Very low
Mx:Wn	0	Some concerns	Undetected	Some concerns	Major concerns	No concerns	Major concerns	Very low
Pc:Pn	0	Some concerns	Undetected	No concerns	Major concerns	No concerns	Major concerns	Very low
Pc:Wn	0	Some concerns	Undetected	No concerns	Major concerns	No concerns	Major concerns	Very low
Pn:Wn	0	Some concerns	Undetected	No concerns	Major concerns	No concerns	Major concerns	Very low

An: almond, Bn: brazil nut, C: control, Hn: Hazelnut, Mx: mixed nuts, Pc: pistachio, Pn: peanut; Wn: walnut.

Table S15. Sensitivity analyses.

Study	SMD	Lower limit	Upper limit
**Body weight**			
Abazarfard, 2014	0.02	-0.02	0.05
Bento, 2014	0.01	-0.02	0.05
Berryman, 2015	0.01	-0.02	0.05
Bowen, 2019	0.01	-0.02	0.05
Chen, 2017	0.01	-0.02	0.05
Coates, 2020	0.01	-0.02	0.05
Damasceno, 2011	0.01	-0.02	0.05
Damasceno, 2011	0.01	-0.02	0.05
Dhillon, 2016	0.02	-0.02	0.05
Dhillon, 2018	0.01	-0.02	0.05
Foster, 2012	0.01	-0.02	0.05
Hollis, 2007	0.02	-0.02	0.05
Jamshed, 2015	0.01	-0.02	0.05
Jenkins, 2002	0.01	-0.02	0.05
Jung, 2017	0.01	-0.02	0.05
Kalgaonkar, 2010	0.01	-0.02	0.05
Kaseb, 2013	0.01	-0.02	0.05
Kaseb, 2013	0.01	-0.02	0.05
Lamarche, 2004	0.01	-0.02	0.05
Lee, 2017	0.01	-0.02	0.05
Liu, 2017	0.01	-0.02	0.05
Ren, 2020	0.01	-0.02	0.05
Ruisinger, 2015	0.01	-0.02	0.05
Sabaté, 2003	0.01	-0.02	0.05
Souza, 2018	0.01	-0.02	0.05
Spiller, 1998	0.01	-0.02	0.05
Tan, 2013	0.01	-0.02	0.05
Tan, 2013	0.01	-0.02	0.05
Tan, 2013	0.01	-0.02	0.05
Tan, 2013	0.01	-0.02	0.05
Wien, 2003	0.02	-0.02	0.05
Wien, 2010	0.01	-0.02	0.05
Williams, 2019	0.01	-0.02	0.05
Maranhão, 2011	0.01	-0.02	0.05
Damavandi, 2019	0.01	-0.02	0.05
Mohan, 2018	0.01	-0.02	0.05
Mukuddem-Petersen, 2007	0.01	-0.02	0.05
Damavandi, 2013	0.01	-0.02	0.05
Mercanligil, 2007	0.01	-0.02	0.05
Orem, 2013	0.01	-0.02	0.05
Pearson, 2016	0.01	-0.02	0.05
Pearson, 2016	0.01	-0.02	0.05
Pearson, 2016	0.01	-0.02	0.05
Tey, 2013	0.01	-0.02	0.05
Tey, 2013	0.01	-0.02	0.05
Yilmaz, 2019	0.01	-0.02	0.05
Somerset, 2013	0.01	-0.02	0.05
Abbaspour, 2019	0.01	-0.02	0.05
Casas-Agustench, 2011	0.01	-0.02	0.05
Chisholm, 2005	0.01	-0.02	0.05
Damasceno, 2013	0.01	-0.02	0.05
Estruch, 2016	0.002	-0.04	0.05
Jenkins, 1997	0.01	-0.02	0.05

Jenkins, 2011	0.01	-0.02	0.05
Lee, 2014	0.01	-0.02	0.05
Njike, 2015	0.01	-0.02	0.05
Razquin, 2010	0.01	-0.02	0.05
Salas-Salvado, 2008	0.02	-0.02	0.05
Álvarez-Pérez, 2016	0.01	-0.02	0.05
Carughi, 2019	0.01	-0.02	0.05
Fantino, 2019	0.01	-0.02	0.05
Gebauer, 2008	0.01	-0.02	0.05
Gulati, 2014	0.01	-0.02	0.05
Hernández-Alonso, 2014	0.01	-0.02	0.05
Kasliwal, 2015	0.01	-0.02	0.05
Li, 2010	0.01	-0.02	0.05
Rock, 2020	0.01	-0.02	0.05
Sauder, 2015	0.01	-0.02	0.05
Sheridan, 2007	0.01	-0.02	0.05
Alves, 2014	0.01	-0.02	0.05
Barbour, 2015	0.01	-0.02	0.05
Caldas, 2020	0.01	-0.02	0.05
Claesson, 2009	0.01	-0.02	0.05
Ghadiminouran, 2009	0.01	-0.02	0.05
Johnston, 2013	0.01	-0.02	0.05
O'Byrne, 1997	0.01	-0.02	0.05
Wang, 2020	0.01	-0.02	0.05
Wien, 2014	0.01	-0.02	0.05
Bashan, 2018	0.01	-0.02	0.05
Bitok, 2018	0.01	-0.02	0.05
Chiang, 2012	0.01	-0.02	0.05
Damasceno, 2011	0.01	-0.02	0.05
Domenech, 2019	0.01	-0.02	0.04
Fatahi, 2019	0.01	-0.02	0.05
Gozde, 2019	0.01	-0.02	0.05
Kaseb, 2013	0.01	-0.02	0.05
Katz, 2012	0.01	-0.02	0.04
Le, 2016	0.01	-0.02	0.05
Ma, 2010	0.01	-0.02	0.05
Mukuddem-Petersen, 2007	0.01	-0.02	0.05
Mukuddem-Petersen, 2007	0.01	-0.02	0.05
Olmedilla-Alonso, 2008	0.01	-0.02	0.05
Rock, 2017	0.01	-0.02	0.05
Ros, 2004	0.01	-0.02	0.05
Sabaté, 2005	0.01	-0.02	0.05
Spaccarotella, 2008	0.01	-0.02	0.05
Tapsell, 2004	0.01	-0.02	0.05
Tapsell, 2009	0.01	-0.02	0.05
Tapsell, 2017	0.01	-0.02	0.05
Tindall, 2019	0.01	-0.02	0.05
Wu, 2010	0.01	-0.02	0.05
Zambón, 2000	0.01	-0.02	0.05
Zibaeenezzhad, 2019	0.01	-0.02	0.05
**Body mass index**			
Abazarfard, 2014	0.01	-0.04	0.06
Abbaspour, 2019	0.002	-0.05	0.05
Abdrabalnabi, 2020	-0.004	-0.06	0.05
Alves, 2014	-0.003	-0.06	0.05
Barbour, 2015	-0.0004	-0.05	0.05
Bento, 2014	0.001	-0.05	0.05
Bulló, 2009	0.004	-0.05	0.06

Caldas, 2020	-0.001	-0.05	0.05
Canales, 2007	0.001	-0.05	0.05
Chen, 2017	0.001	-0.05	0.05
Chisholm, 2005	0.001	-0.05	0.05
Claesson, 2009	0.001	-0.05	0.05
Coates, 2020	-0.001	-0.05	0.05
Costa e Silva, 2019	0.001	-0.05	0.05
Damasceno, 2013	0.0002	-0.05	0.05
Damavandi, 2013	0.001	-0.05	0.05
Damavandi, 2019	0.001	-0.05	0.05
Dikariyanto, 2020	-0.005	-0.06	0.05
Gozde, 2019	0.0001	-0.05	0.05
Hernández-Alonso, 2014	0.001	-0.06	0.05
Hou, 2018	0.001	-0.05	0.05
Jenkins, 2011	-0.001	-0.05	0.05
Jung, 2017	0.001	-0.05	0.05
Kalgaonkar, 2010	-0.01	-0.05	0.05
Kasliwal, 2015	0.0004	-0.05	0.05
Katz, 2012	0.001	-0.06	0.05
Kocyigit, 2006	0.001	-0.05	0.05
Le, 2016	0.003	-0.05	0.05
Lee, 2014	0.001	-0.05	0.05
Li, 2010	0.001	-0.05	0.06
Li, 2011	0.003	-0.05	0.05
Liu, 2017	0.001	-0.05	0.05
Ma, 2010	0.001	-0.05	0.05
Maranhão, 2011	-0.0003	-0.05	0.05
Mercanligil, 2007	0.001	-0.05	0.05
Mohan, 2018	0.001	-0.05	0.05
Morgan, 2000	-0.003	-0.05	0.05
Njike, 2015	0.002	-0.05	0.05
Njike, 2015	0.002	-0.06	0.05
O'Byrne, 1997	0.004	-0.05	0.05
Orem, 2013	-0.001	-0.05	0.05
Parham, 2014	-0.002	-0.05	0.05
Pearson, 2016	-0.003	-0.05	0.05
Pearson, 2016	0.001	-0.05	0.05
Pearson, 2016	0.0003	-0.05	0.05
Ren, 2020	0.0003	-0.05	0.05
Rock, 2017	0.0002	-0.05	0.05
Rock, 2020	0.002	-0.05	0.05
Ruisinger, 2015	0.001	-0.05	0.05
Sabaté, 2005	0.001	-0.05	0.05
Sauder, 2015	0.0004	-0.05	0.05
Schutte, 2006	0.001	-0.05	0.05
Schutte, 2006	0.001	-0.05	0.05
Schutte, 2006	0.002	-0.05	0.05
Sheridan, 2007	0.001	-0.05	0.05
Somerset, 2013	0.0004	-0.05	0.05
Souza, 2018	0.0004	-0.05	0.05
Tan, 2013	0.0005	-0.05	0.05
Tan, 2013	0.0004	-0.05	0.05
Tan, 2013	0.002	-0.05	0.05
Tan, 2013	0.001	-0.05	0.05
Tapsell, 2004	0.001	-0.05	0.05
Tey, 2013	0.001	-0.05	0.05
Tey, 2013	0.01	-0.05	0.05
Wang, 2020	0.002	-0.05	0.05

Wien, 2003	0.001	-0.04	0.06
Wien, 2010	0.01	-0.05	0.05
Wien, 2014	0.001	-0.05	0.05
Williams, 2019	0.001	-0.05	0.06
Yilmaz, 2019	0.001	-0.05	0.05
Zibaeenezzhad, 2019	0.001	-0.05	0.05
Álvarez-Pérez, 2016	0.003	-0.05	0.06
**Waist circumference**			
Barbour, 2015	-0.05	-0.11	0.01
Berryman, 2015	-0.05	-0.11	0.01
Chen, 2017	-0.05	-0.11	0.01
Hernández-Alonso, 2014	-0.04	-0.08	0.004
Hwang, 2019	-0.05	-0.11	0.02
Jung, 2017	-0.05	-0.11	0.01
Katz, 2012	-0.05	-0.11	0.01
Ma, 2010	-0.05	-0.11	0.01
Njike, 2015	-0.05	-0.11	0.01
Park, 2017	-0.04	-0.10	0.02
Williams, 2019	-0.05	-0.11	0.01
Abazarfard, 2014	-0.05	-0.11	0.01
Abbaspour, 2019	-0.05	-0.11	0.01
Álvarez-Pérez, 2016	-0.05	-0.11	0.01
Alves, 2014	-0.05	-0.11	0.01
Bitok, 2018	-0.05	-0.11	0.01
Bowen, 2019	-0.05	-0.11	0.01
Bulló, 2009	-0.05	-0.11	0.01
Caldas, 2020	-0.05	-0.11	0.01
Carughi, 2019	-0.05	-0.11	0.01
Casas-Agustench, 2011	-0.05	-0.11	0.01
Claesson, 2009	-0.05	-0.11	0.01
Coates, 2020	-0.05	-0.11	0.01
Costa e Silva, 2019	-0.05	-0.11	0.004
Damasceno, 2013	-0.05	-0.11	0.01
Damavandi, 2019	-0.05	-0.11	0.01
Dhillon, 2016	-0.05	-0.11	0.01
Dhillon, 2018	-0.05	-0.11	0.02
Dikariyanto, 2020	-0.05	-0.11	0.01
Domenech, 2019	-0.05	-0.11	0.01
Estruch, 2016	-0.05	-0.11	0.01
Fatahi, 2019	-0.05	-0.11	0.01
Gozde, 2019	-0.05	-0.11	0.01
Gulati, 2014	-0.03	-0.08	0.01
Johnston, 2013	-0.05	-0.11	0.01
Kasliwal, 2015	-0.05	-0.11	0.01
Lee, 2014	-0.05	-0.11	0.01
Maranhão, 2011	-0.05	-0.11	0.01
Mohan, 2018	-0.05	-0.11	0.003
Njike, 2015	-0.05	-0.11	0.01
Pearson, 2016	-0.05	-0.11	0.01
Pearson, 2016	-0.05	-0.11	0.01
Pearson, 2016	-0.05	-0.11	0.01
Rock, 2017	-0.05	-0.11	0.01
Schutte, 2006	-0.04	-0.10	0.02
Schutte, 2006	-0.05	-0.11	0.01
Schutte, 2006	-0.05	-0.11	0.01
Somerset, 2013	-0.04	-0.09	0.02
Souza, 2018	-0.05	-0.11	0.01
Tan, 2013	-0.05	-0.11	0.01

Tan, 2013	-0.05	-0.11	0.005
Tan, 2013	-0.05	-0.11	0.01
Tan, 2013	-0.05	-0.11	0.01
Wien, 2003	-0.05	-0.11	0.01
Wien, 2010	-0.05	-0.11	0.01
Wien, 2014	-0.05	-0.11	0.01
Wu, 2010	-0.05	-0.11	0.01
Yilmaz, 2019	-0.05	-0.11	0.01
Wang, 2020	-0.05	-0.11	0.01
Rock, 2020	-0.05	-0.11	0.01
**Body fat percentage**			
Bento, 2014	-0.07	-0.16	0.03
Chen, 2017	-0.07	-0.16	0.02
Coates, 2020	-0.07	-0.16	0.02
Dhillon, 2018	-0.07	-0.16	0.02
Dikariyanto, 2020	-0.08	-0.16	-0.001
Hollis, 2007	-0.07	-0.16	0.03
Li, 2011	-0.07	-0.16	0.03
Liu, 2017	-0.07	-0.16	0.03
Souza, 2018	-0.07	-0.16	0.03
Costa e Silva, 2019	-0.07	-0.16	0.02
Mercanligil, 2007	-0.07	-0.16	0.03
Pearson, 2016	-0.07	-0.16	0.02
Pearson, 2016	-0.07	-0.16	0.02
Pearson, 2016	-0.07	-0.16	0.01
Tey, 2013	-0.07	-0.16	0.02
Tey, 2013	-0.07	-0.16	0.02
Yilmaz, 2019	-0.07	-0.16	0.02
Abbaspour, 2019	-0.07	-0.16	0.02
Casas-Agustench, 2011	-0.06	-0.15	0.03
Njike, 2015	-0.05	-0.14	0.03
Álvarez-Pérez, 2016	-0.06	-0.16	0.03
Carughi, 2019	-0.07	-0.16	0.03
Alves, 2014	-0.06	-0.15	0.03
Claesson, 2009	-0.07	-0.16	0.02
Johnston, 2013	-0.07	-0.16	0.02
O'Byrne, 1997	-0.06	-0.15	0.03
Bitok, 2018	-0.08	-0.17	0.02
Gozde, 2019	-0.07	-0.16	0.02
Njike, 2015	-0.07	-0.16	0.03
Sabaté, 2005	-0.07	-0.16	0.02
Tapsell, 2004	-0.07	-0.16	0.03
Tapsell, 2009	-0.07	-0.16	0.02
Tapsell, 2017	-0.02	-0.10	0.05

Table S16. Publication bias.

	Coefficient	SE	p-value
**Body weight** (n=103)	-0.124	0.115	0.284
**BMI** (n=72)	-0.280	0.273	0.309
**WC** (n=60)	-0.010	0.231	0.977
**BF%** (n=33)	-0.355	0.559	0.531

Table S17. Inconsistency assessment.

Body weight.

Test(s) of heterogeneity:

	Heterogeneity statistic	degrees of freedom	P	I-squared**	Tau-squared
AnC	18.48	29	0.934	0.0%	0.0000
AnWn	0.00	2	1.000	0.0%	0.0000
BnC	0.00	0	.	.%	0.0000
CsC	0.36	2	0.837	0.0%	0.0000
HnC	1.83	8	0.986	0.0%	0.0000
MdC	0.00	0	.	.%	0.0000
MxC	2.59	11	0.995	0.0%	0.0000
PcC	0.55	9	1.000	0.0%	0.0000
PnC	10.45	8	0.235	23.4%	0.0174
WnC	11.86	23	0.973	0.0%	0.0000
WnCs	0.00	0	.	.%	0.0000
Overall	53.15	102	1.000	0.0%	0.0000

** I-squared: the variation in ES attributable to heterogeneity)

Body mass index.

Test(s) of heterogeneity:

	Heterogeneity statistic	degrees of freedom	P	I-squared**	Tau-squared
AnC	28.07	17	0.044	39.4%	0.0371
MxC	0.62	7	0.999	0.0%	0.0000
WnC	7.24	11	0.780	0.0%	0.0000
PnC	3.41	6	0.756	0.0%	0.0000
BnC	0.00	1	0.960	0.0%	0.0000
HnC	2.50	8	0.962	0.0%	0.0000
CsC	0.10	2	0.949	0.0%	0.0000
PcC	4.40	7	0.732	0.0%	0.0000
PnAn	0.00	0	.	.%	0.0000
AnWn	0.00	0	.	.%	0.0000
PaC	0.00	0	.	.%	0.0000
WnCs	0.00	0	.	.%	0.0000
MdC	0.00	0	.	.%	0.0000
Overall	51.06	71	0.964	0.0%	0.0000

** I-squared: the variation in ES attributable to heterogeneity)

Waist circumference.

Test(s) of heterogeneity:

	Heterogeneity statistic	degrees of freedom	P	I-squared**	Tau-squared
AnC	23.89	16	0.092	33.0%	0.0259
BnC	0.09	1	0.766	0.0%	0.0000
CsC	0.08	2	0.962	0.0%	0.0000
HnC	1.79	3	0.618	0.0%	0.0000
MdC	0.00	0	.	.%	0.0000
MxC	18.98	7	0.008	63.1%	0.0418
PcC	2.79	4	0.593	0.0%	0.0000
PnC	2.79	6	0.834	0.0%	0.0000
WnC	14.10	11	0.227	22.0%	0.0086
WnCs	0.00	0	.	.%	0.0000
Overall	79.06	59	0.042	25.4%	0.0104

** I-squared: the variation in ES attributable to heterogeneity)

Body fat percentage.

Test(s) of heterogeneity:

	Heterogeneity statistic	degrees of freedom	P	I-squared**	Tau-squared
AnC	4.33	8	0.826	0.0%	0.0000
BnC	0.00	0	.	.%	0.0000
HnC	2.41	6	0.878	0.0%	0.0000
MxC	4.28	3	0.233	29.9%	0.0274
PcC	0.00	0	.	.%	0.0000
PnC	2.68	3	0.444	0.0%	0.0000
WnC	18.84	6	0.004	68.2%	0.0705
Overall	37.87	32	0.219	15.5%	0.0099

** I-squared: the variation in ES attributable to heterogeneity)

Figure S1. Network diagrams.

i. Body weight

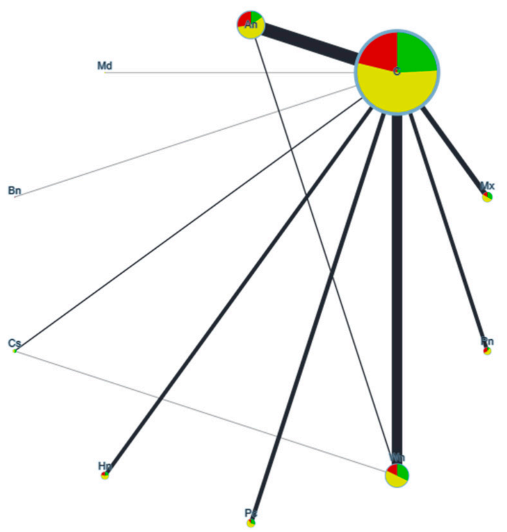

ii. Body mass index

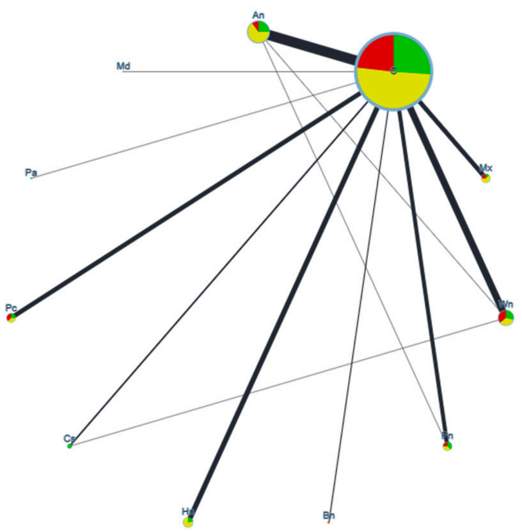

iii.Waist circumference

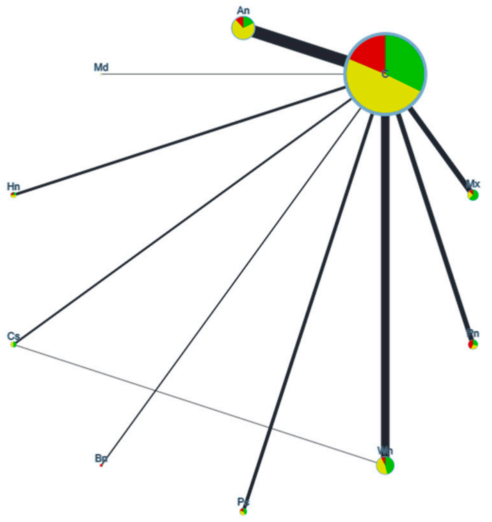

iv. Body fat percentage

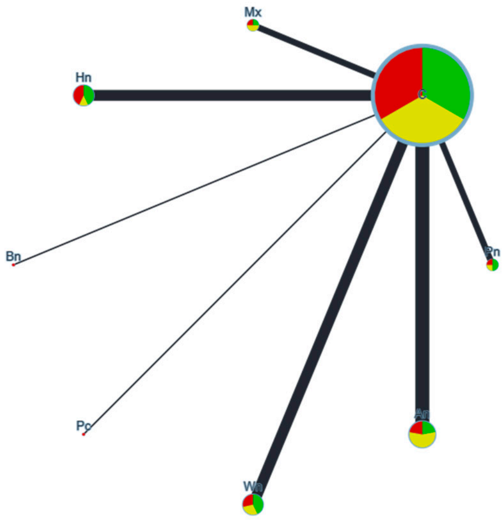

Figure S2. Pairwise meta-analyses for all outcomes.

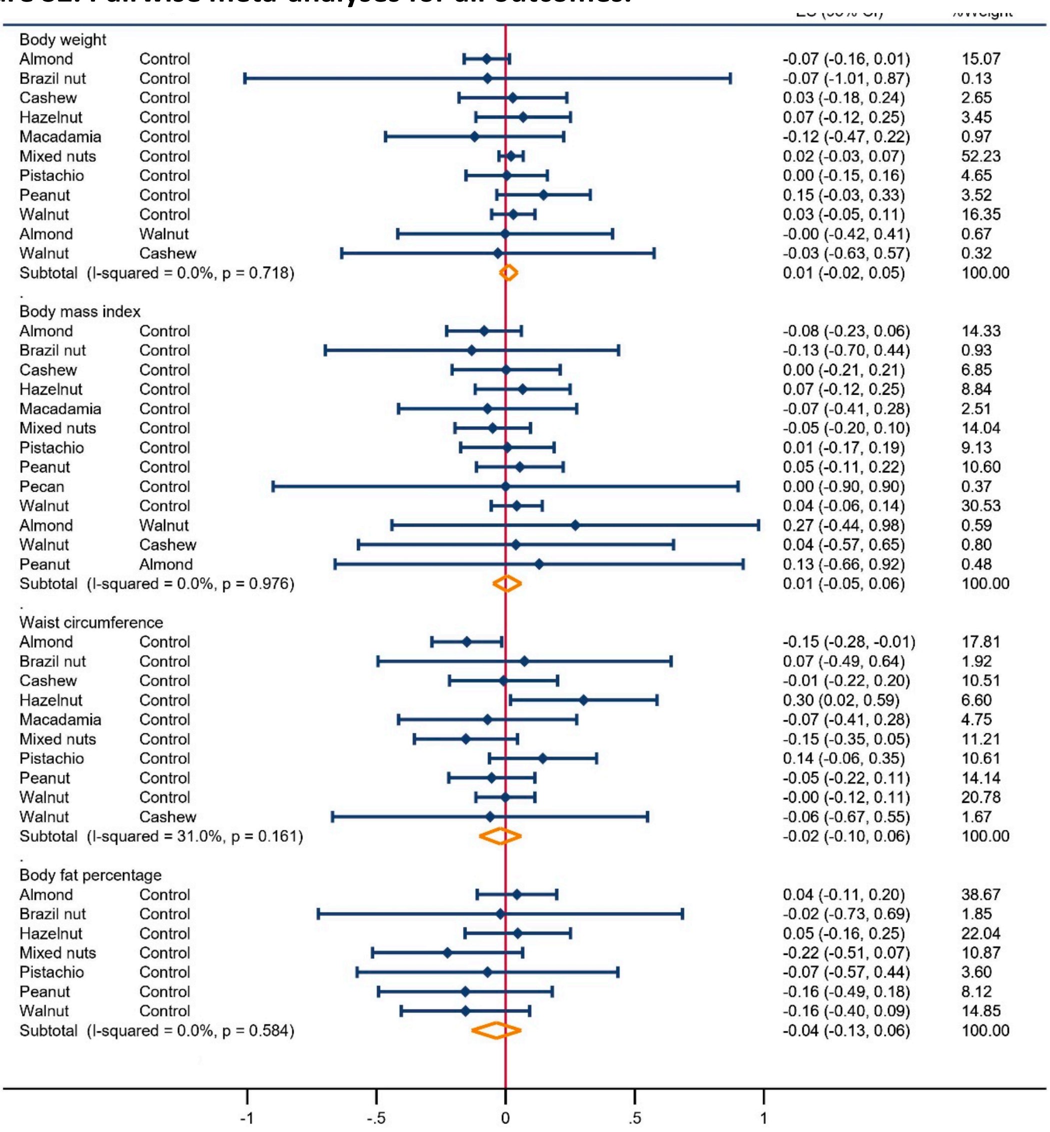

Figure S3. Network estimates for body weight
(ES: standardized mean difference).

a)

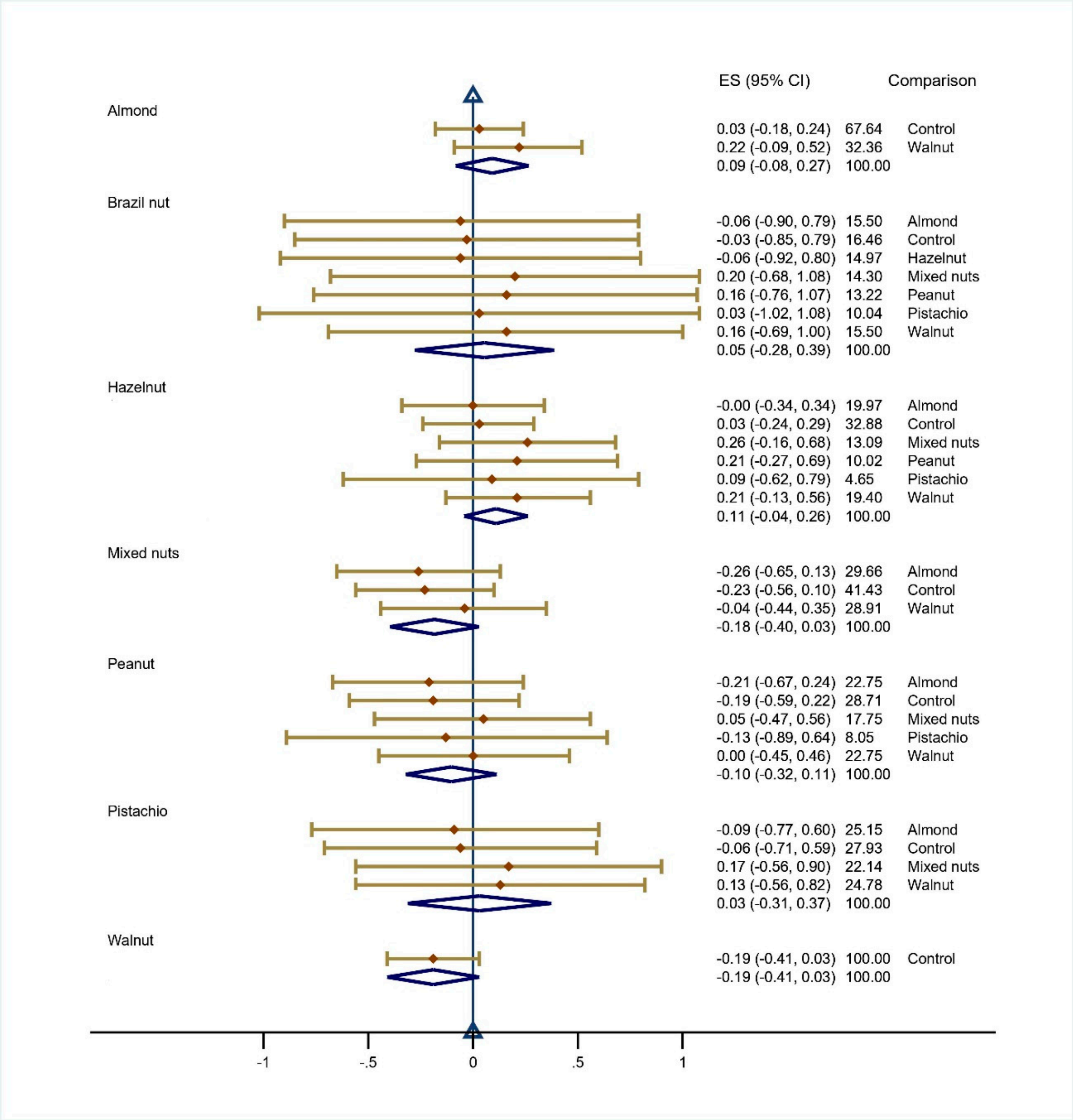

b)

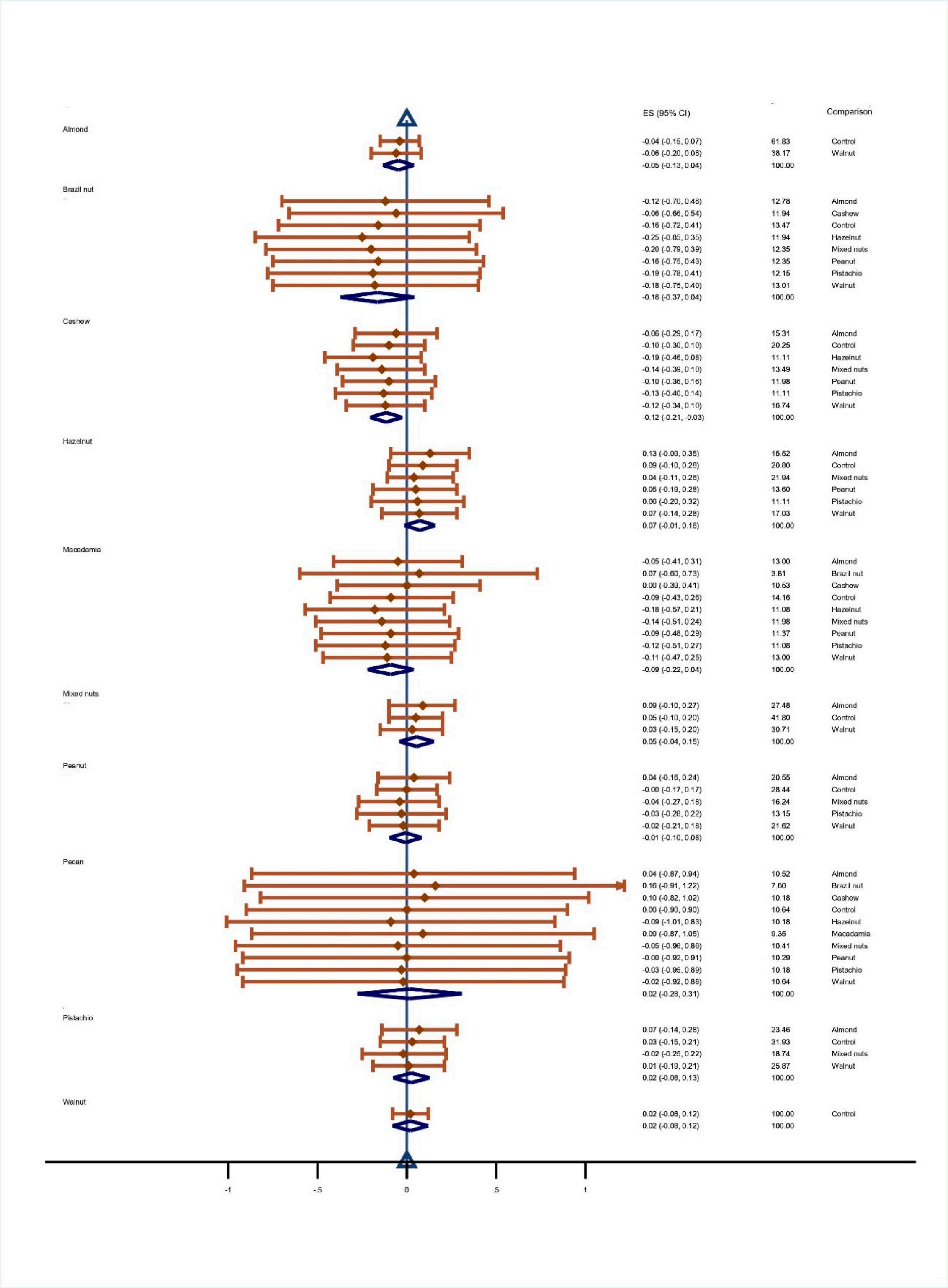
ES (95% CI)
Comparison
Almond
-0.04 (-0.15, 0.07) 61.83 Control
-0.06 (-0.20, 0.08) 38.17 Walnut
-0.05 (-0.13, 0.04) 100.00
Brazil nut
-0.12 (-0.70, 0.46) 12.78 Almond
-0.06 (-0.66, 0.54) 11.94 Cashew
-0.16 (-0.72, 0.41) 13.47 Control
-0.25 (-0.85, 0.35) 11.94 Hazelnut
-0.20 (-0.79, 0.39) 12.35 Mixed nuts
-0.16 (-0.75, 0.43) 12.35 Peanut
-0.19 (-0.78, 0.41) 12.15 Pistachio
-0.18 (-0.75, 0.40) 13.01 Walnut
-0.16 (-0.37, 0.04) 100.00
Cashew
-0.06 (-0.29, 0.17) 15.31 Almond
-0.10 (-0.30, 0.10) 20.25 Control
-0.19 (-0.46, 0.08) 11.11 Hazelnut
-0.14 (-0.39, 0.10) 13.49 Mixed nuts
-0.10 (-0.36, 0.16) 11.98 Peanut
-0.13 (-0.40, 0.14) 11.11 Pistachio
-0.12 (-0.34, 0.10) 16.74 Walnut
-0.12 (-0.21, -0.03) 100.00
Hazelnut
0.13 (-0.09, 0.35) 15.52 Almond
0.09 (-0.10, 0.28) 20.80 Control
0.04 (-0.11, 0.26) 21.94 Mixed nuts
0.05 (-0.19, 0.28) 13.60 Peanut
0.06 (-0.20, 0.32) 11.11 Pistachio
0.07 (-0.14, 0.28) 17.03 Walnut
0.07 (-0.01, 0.16) 100.00
Macadamia
-0.05 (-0.41, 0.31) 13.00 Almond
0.07 (-0.60, 0.73) 3.81 Brazil nut
0.00 (-0.39, 0.41) 10.53 Cashew
-0.09 (-0.43, 0.26) 14.16 Control
-0.18 (-0.57, 0.21) 11.08 Hazelnut
-0.14 (-0.51, 0.24) 11.98 Mixed nuts
-0.09 (-0.48, 0.29) 11.37 Peanut
-0.12 (-0.51, 0.27) 11.08 Pistachio
-0.11 (-0.47, 0.25) 13.00 Walnut
-0.09 (-0.22, 0.04) 100.00
Mixed nuts
0.09 (-0.10, 0.27) 27.48 Almond
0.05 (-0.10, 0.20) 41.80 Control
0.03 (-0.15, 0.20) 30.71 Walnut
0.05 (-0.04, 0.15) 100.00
Peanut
0.04 (-0.16, 0.24) 20.55 Almond
-0.00 (-0.17, 0.17) 28.44 Control
-0.04 (-0.27, 0.18) 16.24 Mixed nuts
-0.03 (-0.28, 0.22) 13.15 Pistachio
-0.02 (-0.21, 0.18) 21.62 Walnut
-0.01 (-0.10, 0.08) 100.00
Pecan
0.04 (-0.87, 0.94) 10.52 Almond
0.16 (-0.91, 1.22) 7.60 Brazil nut
0.10 (-0.82, 1.02) 10.18 Cashew
0.00 (-0.90, 0.90) 10.64 Control
-0.09 (-1.01, 0.83) 10.18 Hazelnut
0.09 (-0.87, 1.05) 9.35 Macadamia
-0.05 (-0.96, 0.86) 10.41 Mixed nuts
-0.00 (-0.92, 0.91) 10.29 Peanut
-0.03 (-0.95, 0.89) 10.18 Pistachio
-0.02 (-0.92, 0.88) 10.64 Walnut
0.02 (-0.28, 0.31) 100.00
Pistachio
0.07 (-0.14, 0.28) 23.46 Almond
0.03 (-0.15, 0.21) 31.93 Control
-0.02 (-0.25, 0.22) 18.74 Mixed nuts
0.01 (-0.19, 0.21) 25.87 Walnut
0.02 (-0.08, 0.13) 100.00
Walnut
0.02 (-0.08, 0.12) 100.00 Control
0.02 (-0.08, 0.12) 100.00
-1
-.5
0
.5
1

c)

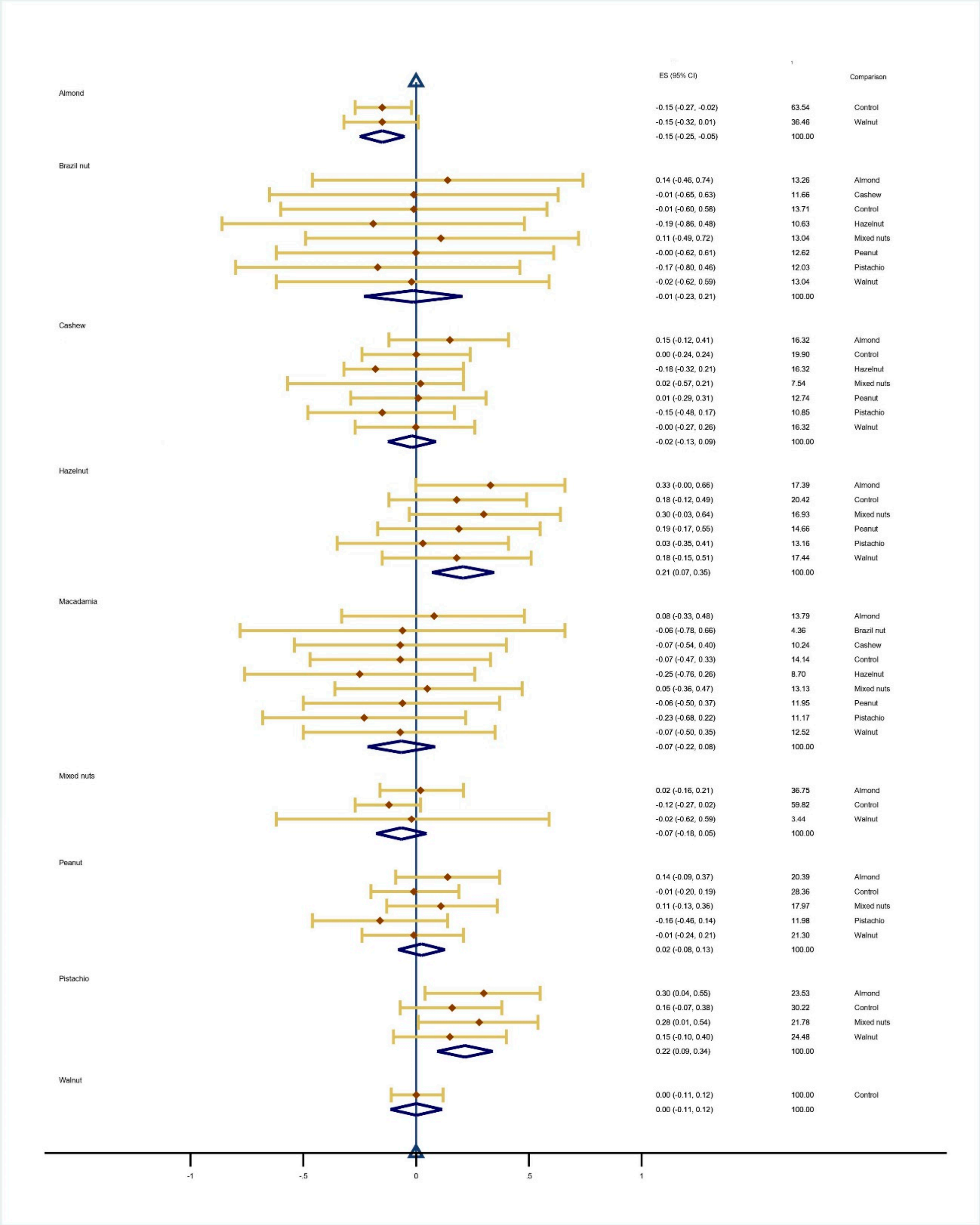

ES (95% CI)
Comparison
Almond
-0.15 (-0.27, -0.02) 63.54 Control
-0.15 (-0.32, 0.01) 36.46 Walnut
-0.15 (-0.25, -0.05) 100.00
Brazil nut
0.14 (-0.46, 0.74) 13.26 Almond
-0.01 (-0.65, 0.63) 11.66 Cashew
-0.01 (-0.60, 0.58) 13.71 Control
-0.19 (-0.86, 0.48) 10.63 Hazelnut
0.11 (-0.49, 0.72) 13.04 Mixed nuts
-0.00 (-0.62, 0.61) 12.62 Peanut
-0.17 (-0.80, 0.46) 12.03 Pistachio
-0.02 (-0.62, 0.59) 13.04 Walnut
-0.01 (-0.23, 0.21) 100.00
Cashew
0.15 (-0.12, 0.41) 16.32 Almond
0.00 (-0.24, 0.24) 19.90 Control
-0.18 (-0.32, 0.21) 16.32 Hazelnut
0.02 (-0.57, 0.21) 7.54 Mixed nuts
0.01 (-0.29, 0.31) 12.74 Peanut
-0.15 (-0.48, 0.17) 10.85 Pistachio
-0.00 (-0.27, 0.26) 16.32 Walnut
-0.02 (-0.13, 0.09) 100.00
Hazelnut
0.33 (-0.00, 0.66) 17.39 Almond
0.18 (-0.12, 0.49) 20.42 Control
0.30 (-0.03, 0.64) 16.93 Mixed nuts
0.19 (-0.17, 0.55) 14.66 Peanut
0.03 (-0.35, 0.41) 13.16 Pistachio
0.18 (-0.15, 0.51) 17.44 Walnut
0.21 (0.07, 0.35) 100.00
Macadamia
0.08 (-0.33, 0.48) 13.79 Almond
-0.06 (-0.78, 0.66) 4.36 Brazil nut
-0.07 (-0.54, 0.40) 10.24 Cashew
-0.07 (-0.47, 0.33) 14.14 Control
-0.25 (-0.76, 0.26) 8.70 Hazelnut
0.05 (-0.36, 0.47) 13.13 Mixed nuts
-0.06 (-0.50, 0.37) 11.95 Peanut
-0.23 (-0.68, 0.22) 11.17 Pistachio
-0.07 (-0.50, 0.35) 12.52 Walnut
-0.07 (-0.22, 0.08) 100.00
Mixed nuts
0.02 (-0.16, 0.21) 36.75 Almond
-0.12 (-0.27, 0.02) 59.82 Control
-0.02 (-0.62, 0.59) 3.44 Walnut
-0.07 (-0.18, 0.05) 100.00
Peanut
0.14 (-0.09, 0.37) 20.39 Almond
-0.01 (-0.20, 0.19) 28.36 Control
0.11 (-0.13, 0.36) 17.97 Mixed nuts
-0.16 (-0.46, 0.14) 11.98 Pistachio
-0.01 (-0.24, 0.21) 21.30 Walnut
0.02 (-0.08, 0.13) 100.00
Pistachio
0.30 (0.04, 0.55) 23.53 Almond
0.16 (-0.07, 0.38) 30.22 Control
0.28 (0.01, 0.54) 21.78 Mixed nuts
0.15 (-0.10, 0.40) 24.48 Walnut
0.22 (0.09, 0.34) 100.00
Walnut
0.00 (-0.11, 0.12) 100.00 Control
0.00 (-0.11, 0.12) 100.00
-1
-.5
0
.5
1

d)

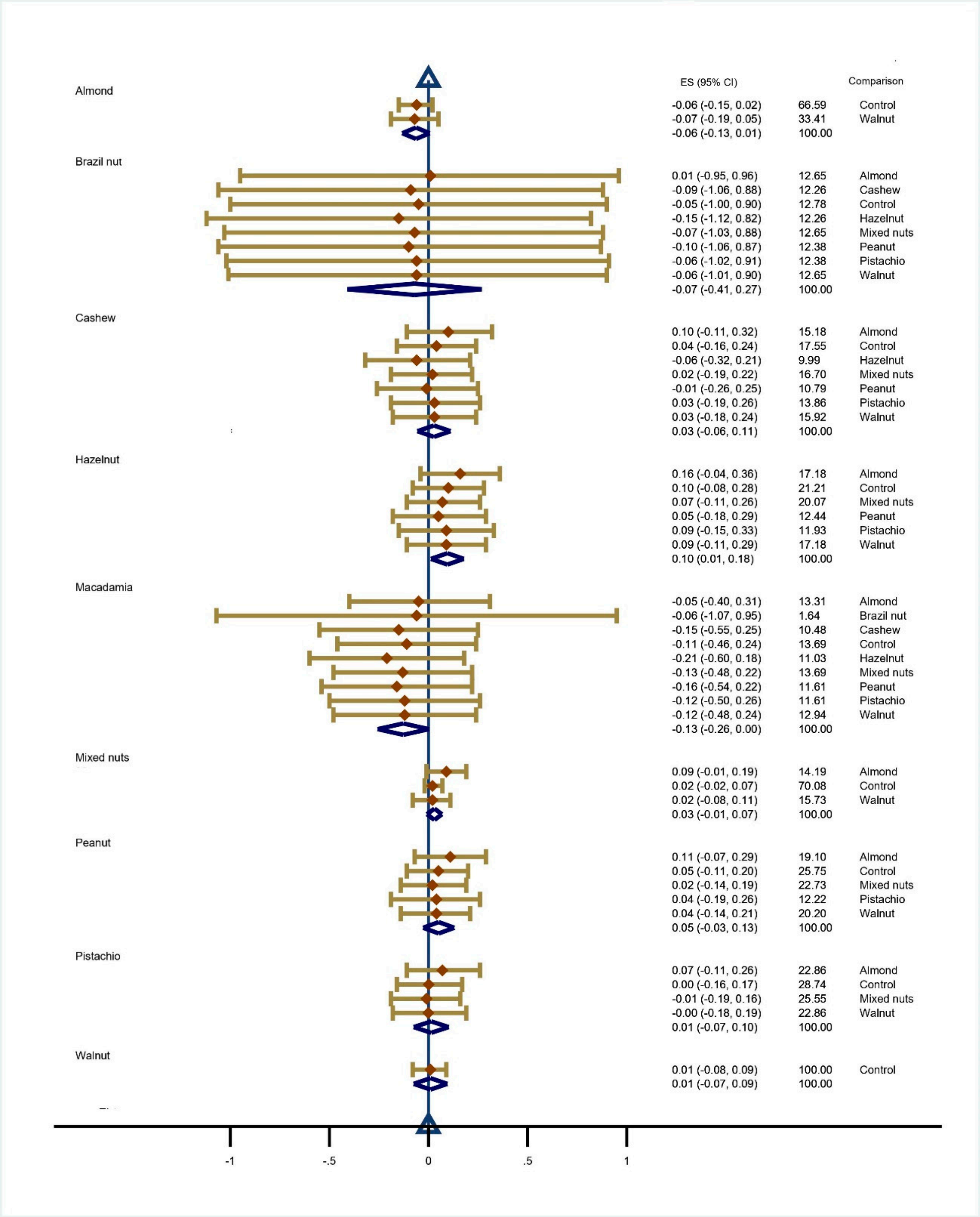
ES (95% CI)
Comparison
Almond
-0.06 (-0.15, 0.02) 66.59 Control
-0.07 (-0.19, 0.05) 33.41 Walnut
-0.06 (-0.13, 0.01) 100.00
Brazil nut
0.01 (-0.95, 0.96) 12.65 Almond
-0.09 (-1.06, 0.88) 12.26 Cashew
-0.05 (-1.00, 0.90) 12.78 Control
-0.15 (-1.12, 0.82) 12.26 Hazelnut
-0.07 (-1.03, 0.88) 12.65 Mixed nuts
-0.10 (-1.06, 0.87) 12.38 Peanut
-0.06 (-1.02, 0.91) 12.38 Pistachio
-0.06 (-1.01, 0.90) 12.65 Walnut
-0.07 (-0.41, 0.27) 100.00
Cashew
0.10 (-0.11, 0.32) 15.18 Almond
0.04 (-0.16, 0.24) 17.55 Control
-0.06 (-0.32, 0.21) 9.99 Hazelnut
0.02 (-0.19, 0.22) 16.70 Mixed nuts
-0.01 (-0.26, 0.25) 10.79 Peanut
0.03 (-0.19, 0.26) 13.86 Pistachio
0.03 (-0.18, 0.24) 15.92 Walnut
0.03 (-0.06, 0.11) 100.00
Hazelnut
0.16 (-0.04, 0.36) 17.18 Almond
0.10 (-0.08, 0.28) 21.21 Control
0.07 (-0.11, 0.26) 20.07 Mixed nuts
0.05 (-0.18, 0.29) 12.44 Peanut
0.09 (-0.15, 0.33) 11.93 Pistachio
0.09 (-0.11, 0.29) 17.18 Walnut
0.10 (0.01, 0.18) 100.00
Macadamia
-0.05 (-0.40, 0.31) 13.31 Almond
-0.06 (-1.07, 0.95) 1.64 Brazil nut
-0.15 (-0.55, 0.25) 10.48 Cashew
-0.11 (-0.46, 0.24) 13.69 Control
-0.21 (-0.60, 0.18) 11.03 Hazelnut
-0.13 (-0.48, 0.22) 13.69 Mixed nuts
-0.16 (-0.54, 0.22) 11.61 Peanut
-0.12 (-0.50, 0.26) 11.61 Pistachio
-0.12 (-0.48, 0.24) 12.94 Walnut
-0.13 (-0.26, 0.00) 100.00
Mixed nuts
0.09 (-0.01, 0.19) 14.19 Almond
0.02 (-0.02, 0.07) 70.08 Control
0.02 (-0.08, 0.11) 15.73 Walnut
0.03 (-0.01, 0.07) 100.00
Peanut
0.11 (-0.07, 0.29) 19.10 Almond
0.05 (-0.11, 0.20) 25.75 Control
0.02 (-0.14, 0.19) 22.73 Mixed nuts
0.04 (-0.19, 0.26) 12.22 Pistachio
0.04 (-0.14, 0.21) 20.20 Walnut
0.05 (-0.03, 0.13) 100.00
Pistachio
0.07 (-0.11, 0.26) 22.86 Almond
0.00 (-0.16, 0.17) 28.74 Control
-0.01 (-0.19, 0.16) 25.55 Mixed nuts
-0.00 (-0.18, 0.19) 22.86 Walnut
0.01 (-0.07, 0.10) 100.00
Walnut
0.01 (-0.08, 0.09) 100.00 Control
0.01 (-0.07, 0.09) 100.00
-1
-.5
0
.5
1

Figure S4. Subgroup analyses.

(Note: Effect sizes – ES – are standardized mean differences)

i) Nuts vs. body weight by study design

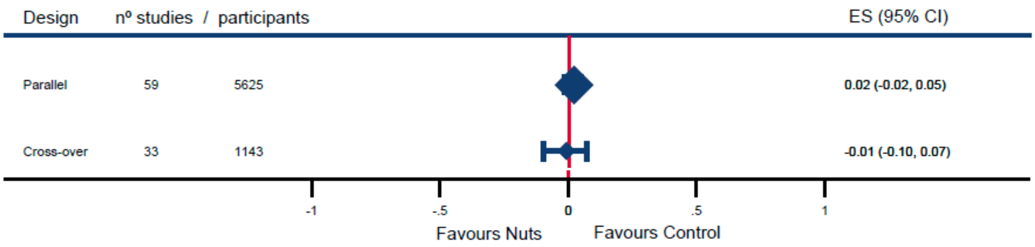

ii) Nuts vs. body weight by nut type

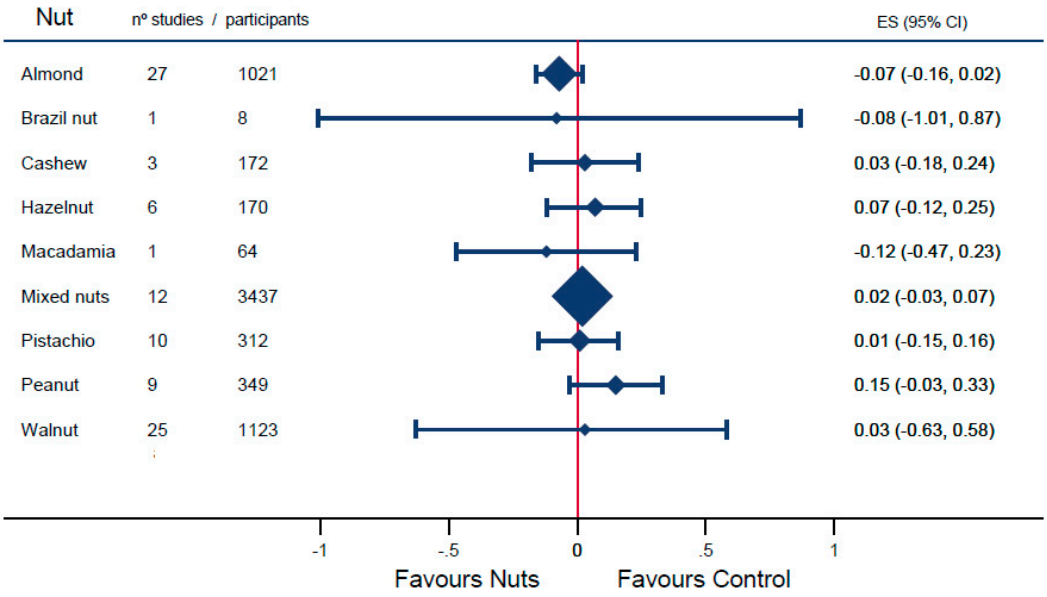

iii) Nuts vs. body weight by health status of the participants.

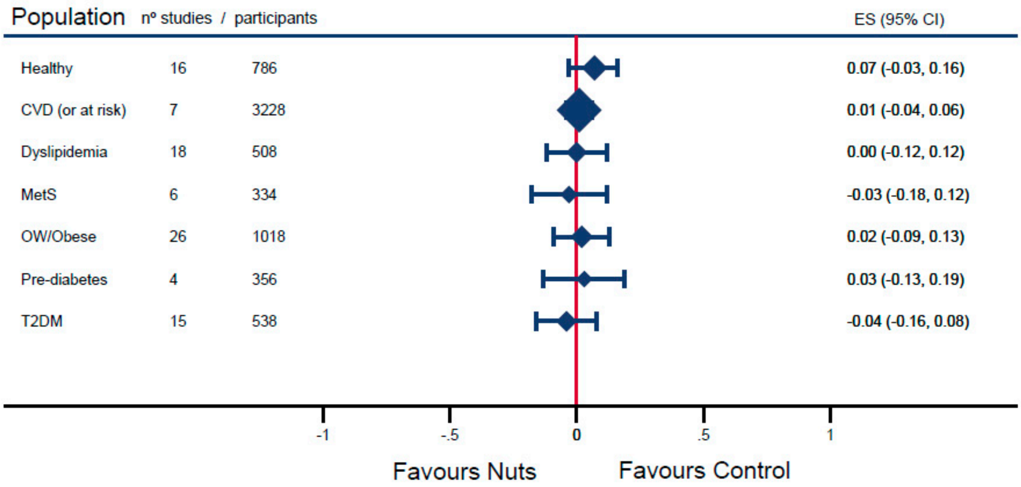

iv) Nuts vs. body weight: sensitivity analysis including only RCTs aimed at losing weight.

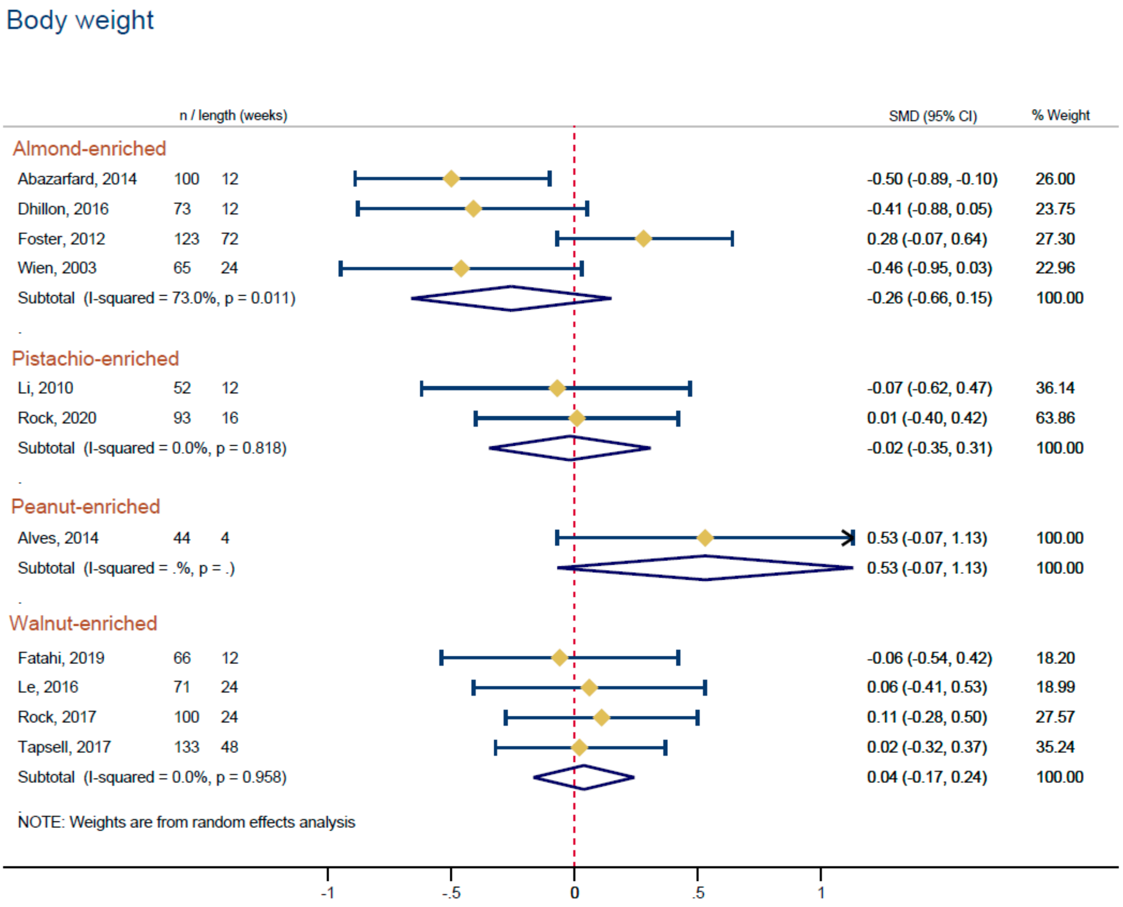

v) Nuts vs. body mass index by study design

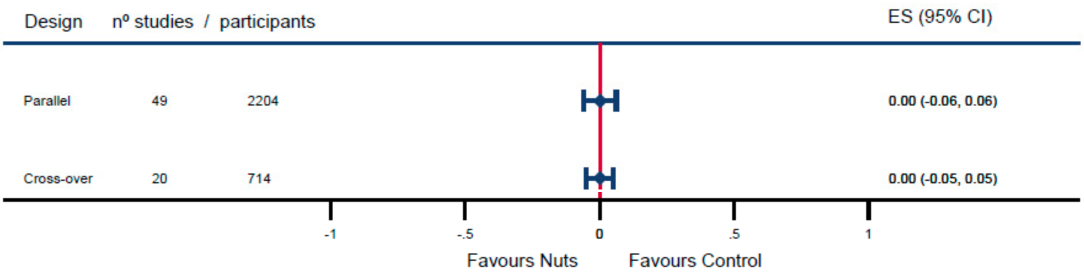

vi) Nuts vs. body mass index by nut type

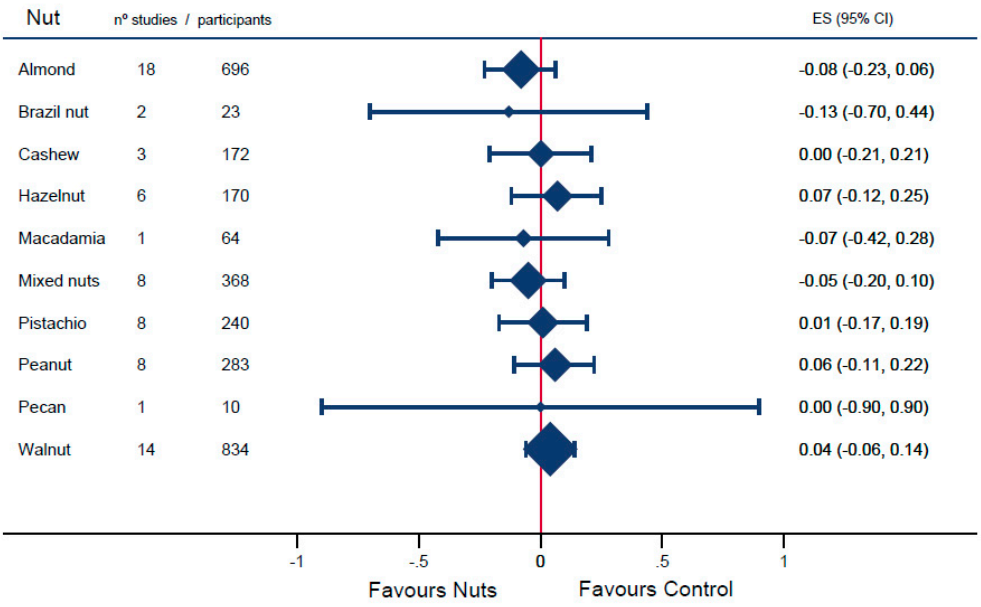

vii) Nuts vs. body mass index by health status of the participants.

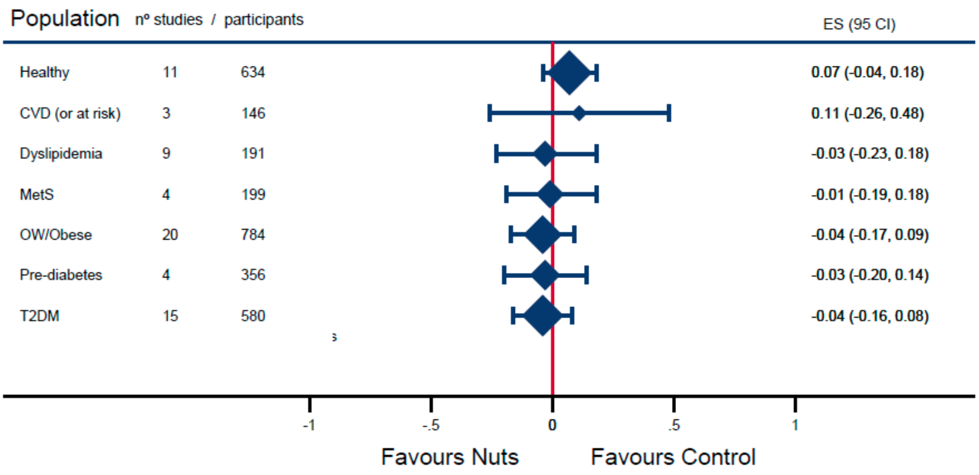

viii) Nuts vs. body mass index: sensitivity analysis including only RCTs aimed at losing weight.

BMI

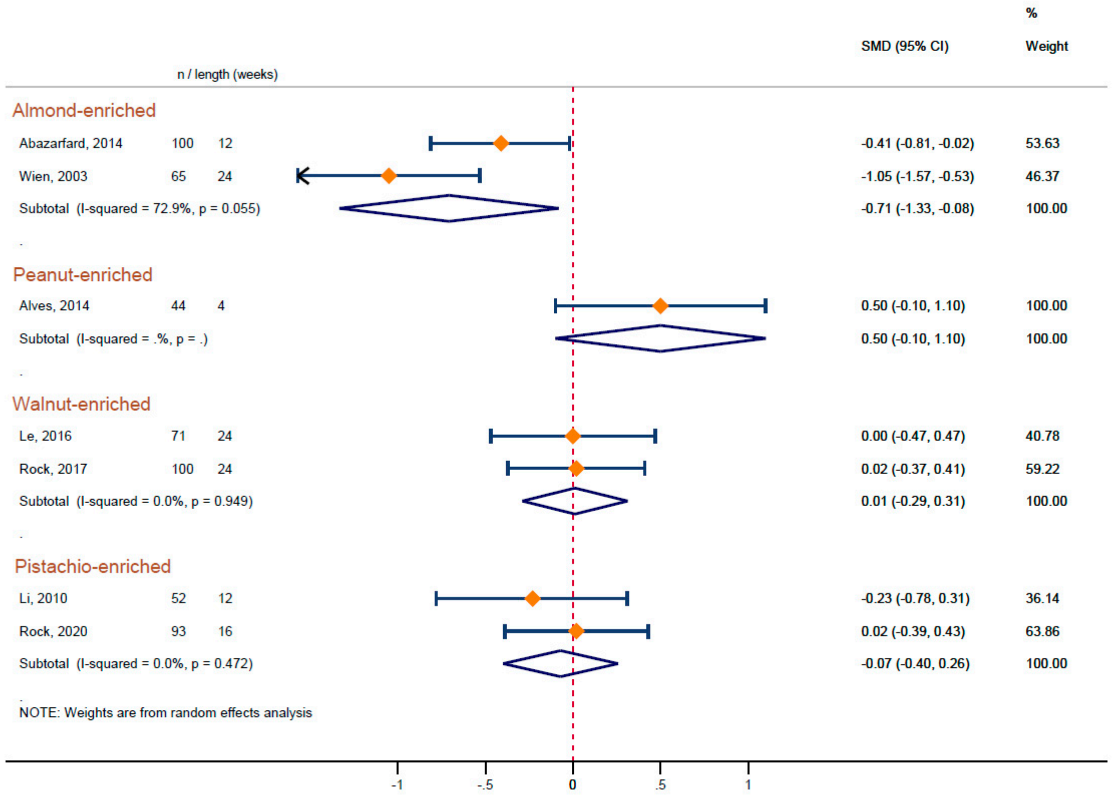

ix) Nuts vs. waist circumference by study design

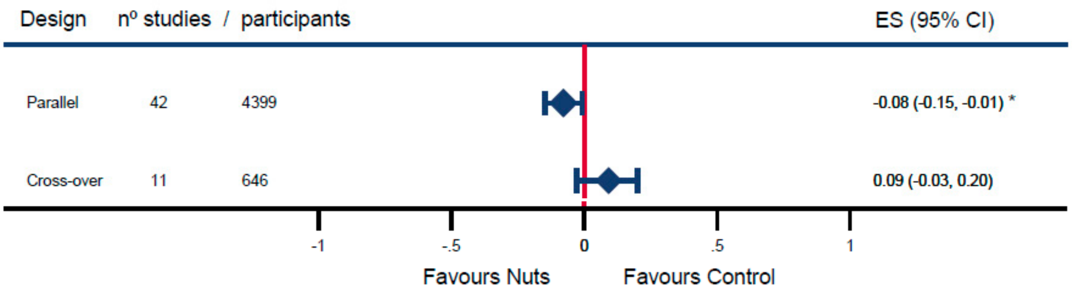

x) Nuts vs. waist circumference by nut type

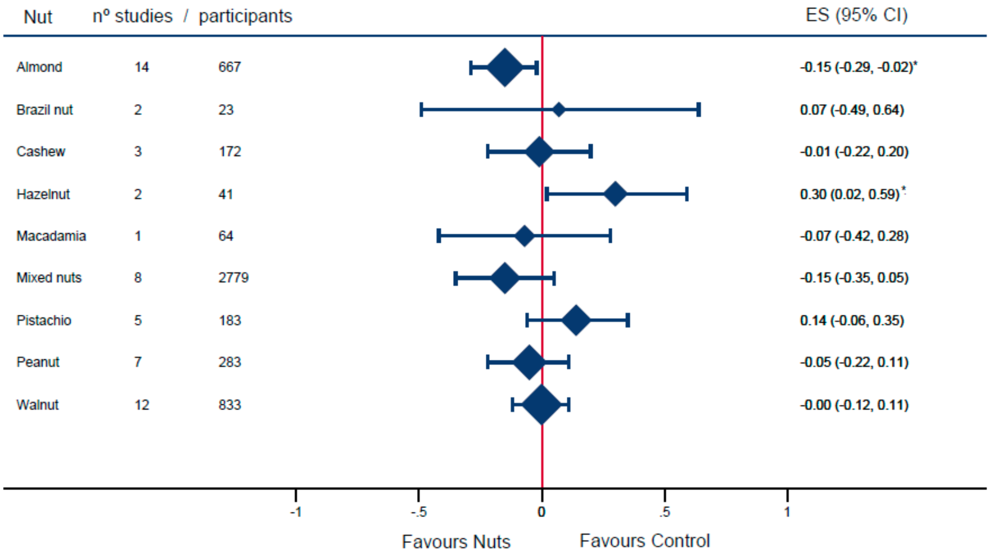

xi) Nuts vs. waist circumference by health status of the participants.

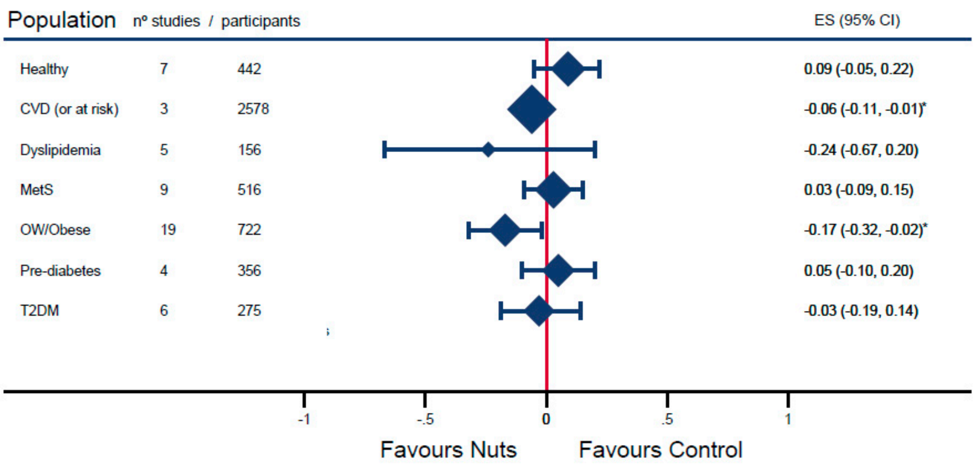

xii) Nuts vs. waist circumference: sensitivity analysis including only RCTs aimed at losing weight.

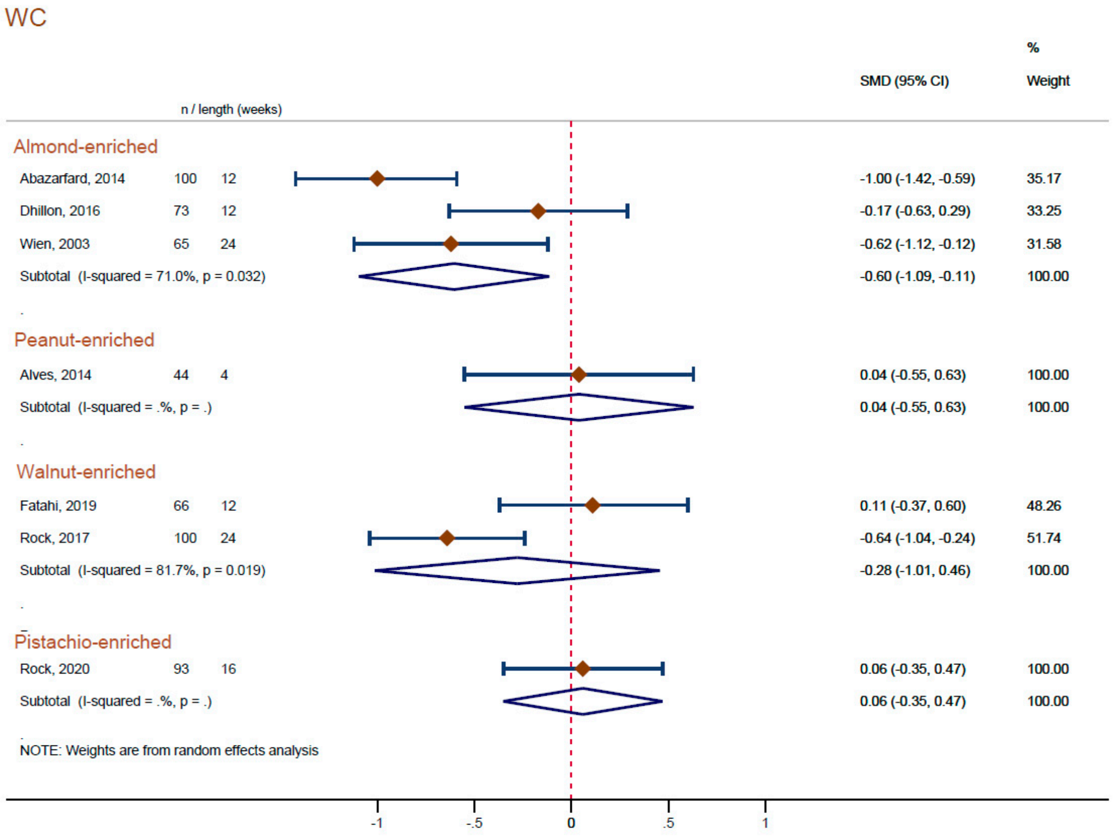

xiii) Nuts vs. body fat percentage by study design

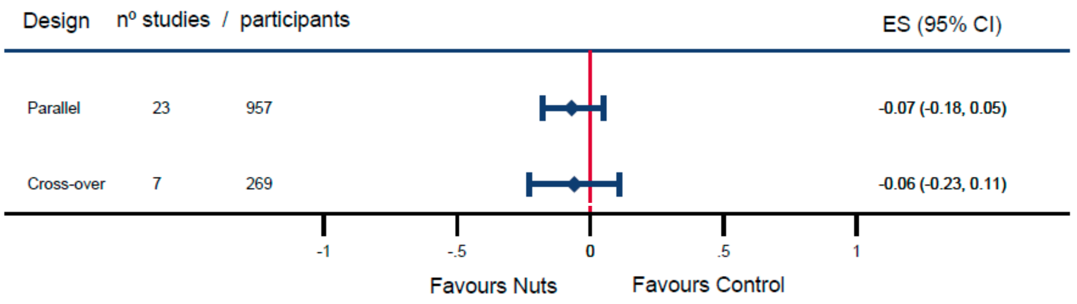

xiv) Nuts vs. body fat percentage by nut type

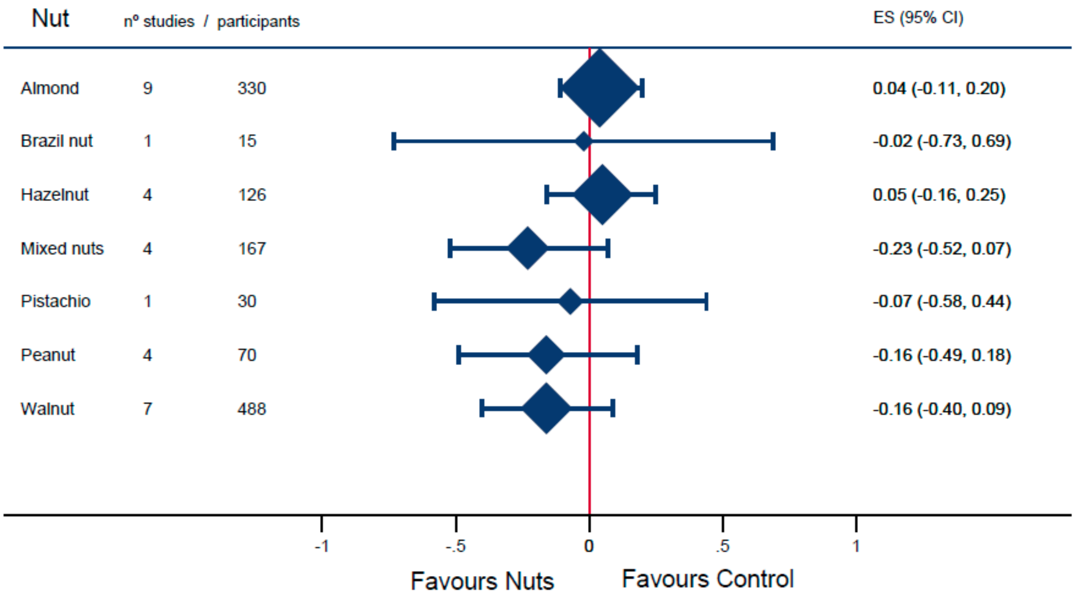

xv) Nuts vs. body fat percentage by health status of the participants.

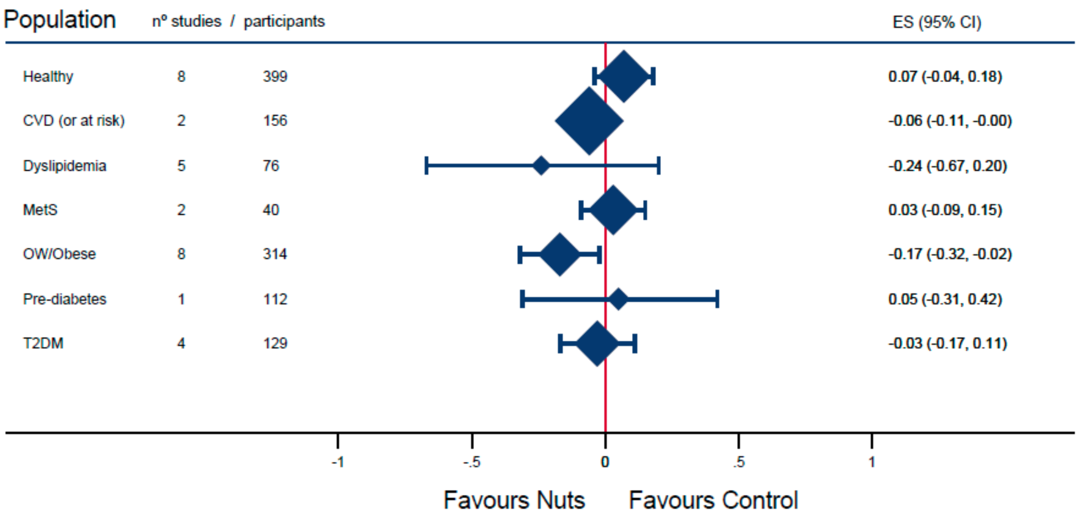

xvi) Nuts vs. body fat percentage: sensitivity analysis including only RCTs aimed at losing weight.

BF %

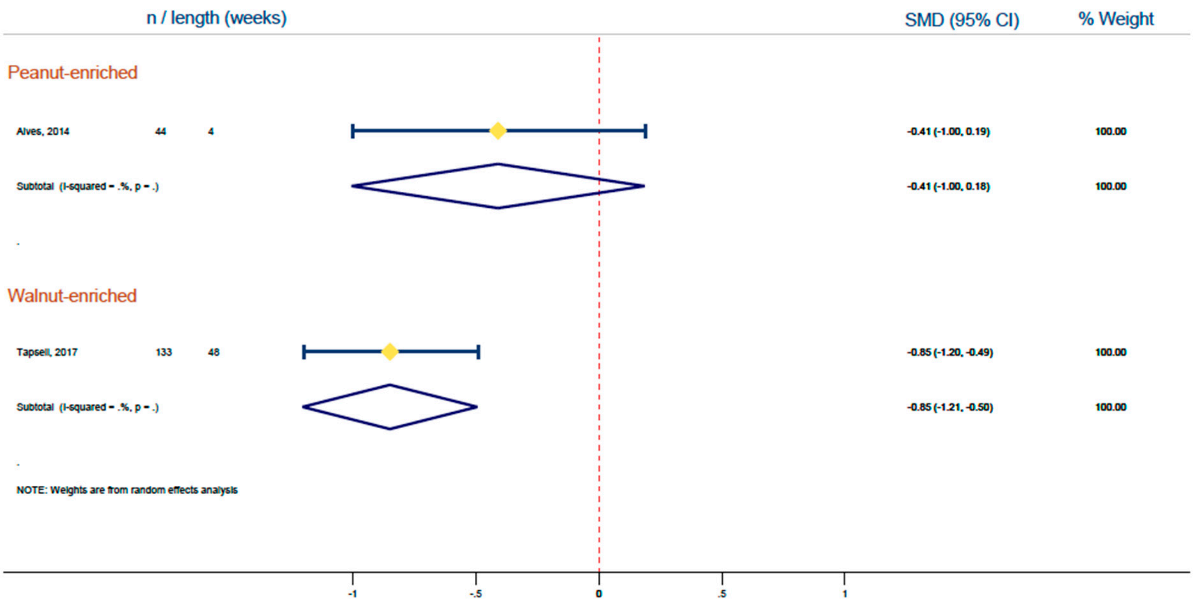

Figure S5. Subgroup analyses based on control comparison for all outcomes:

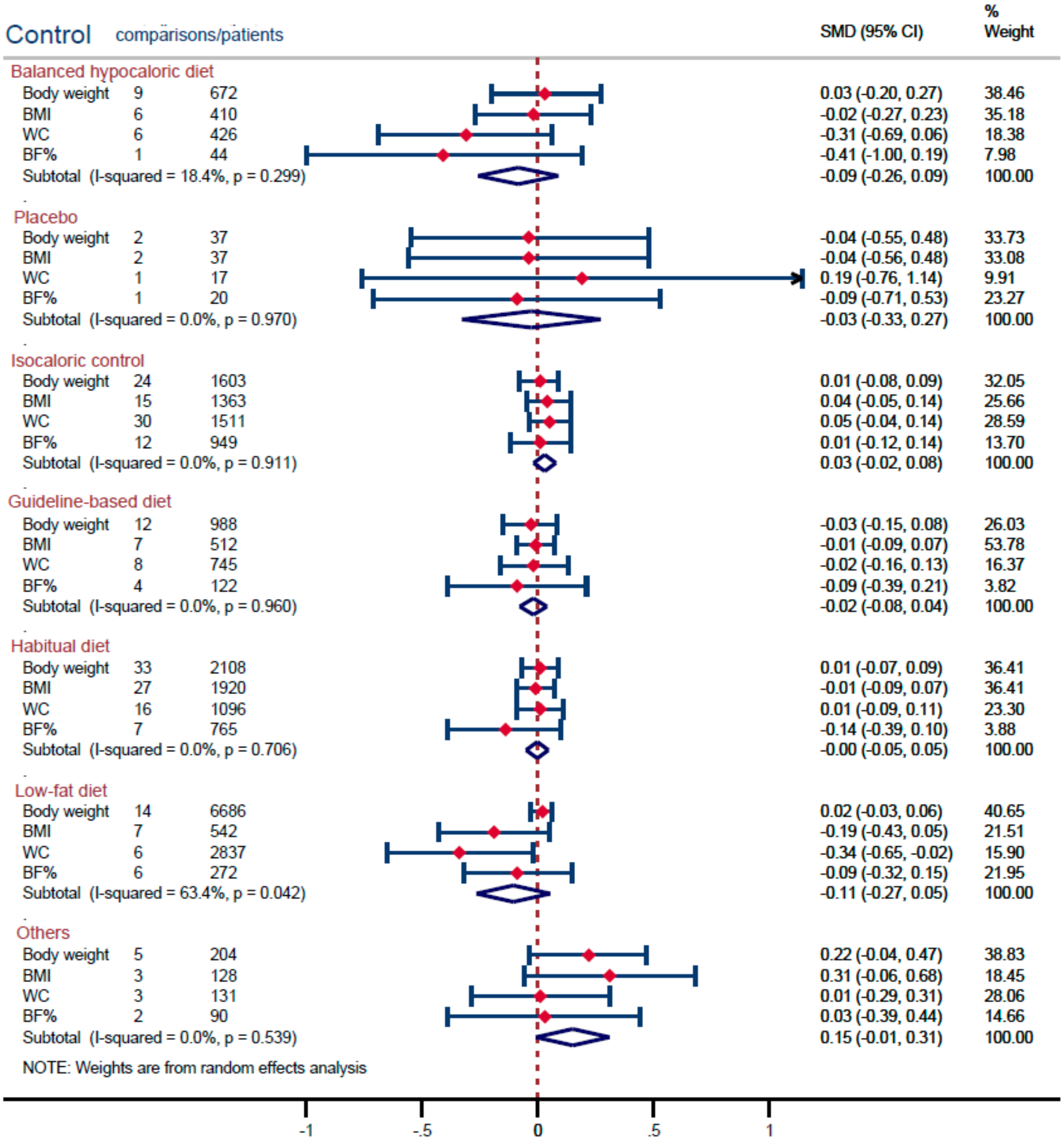

Figure S6. Meta-regressions.

Body weight

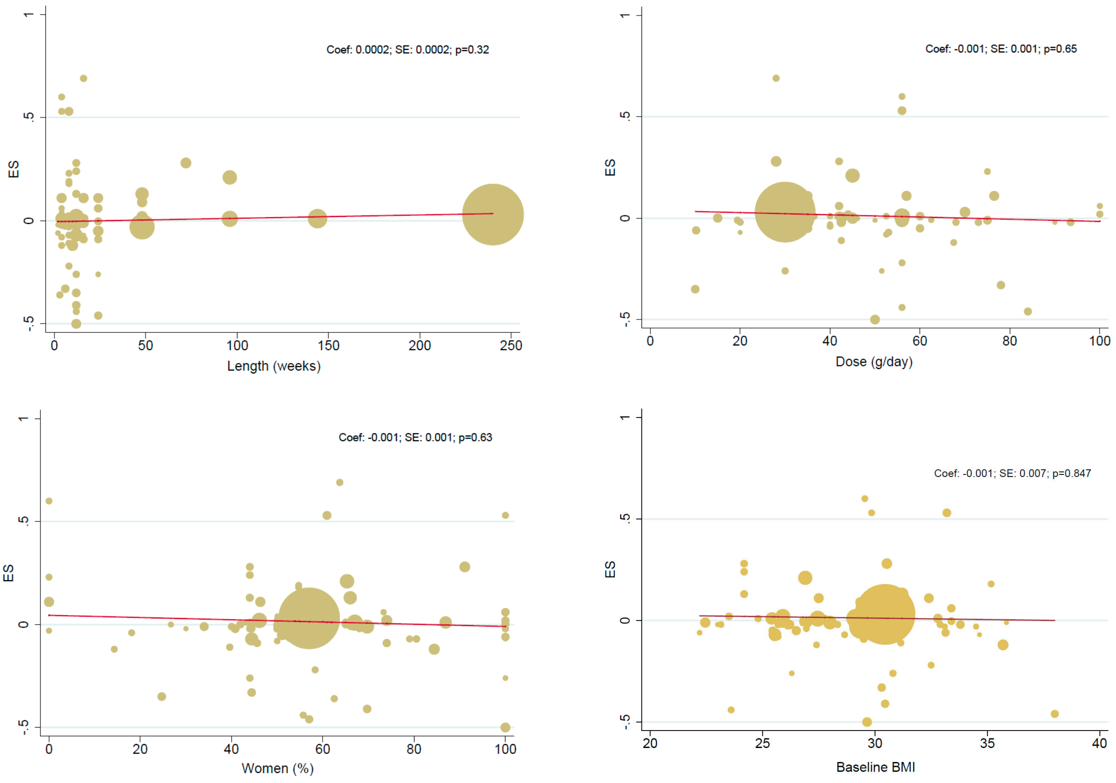

Body mass index

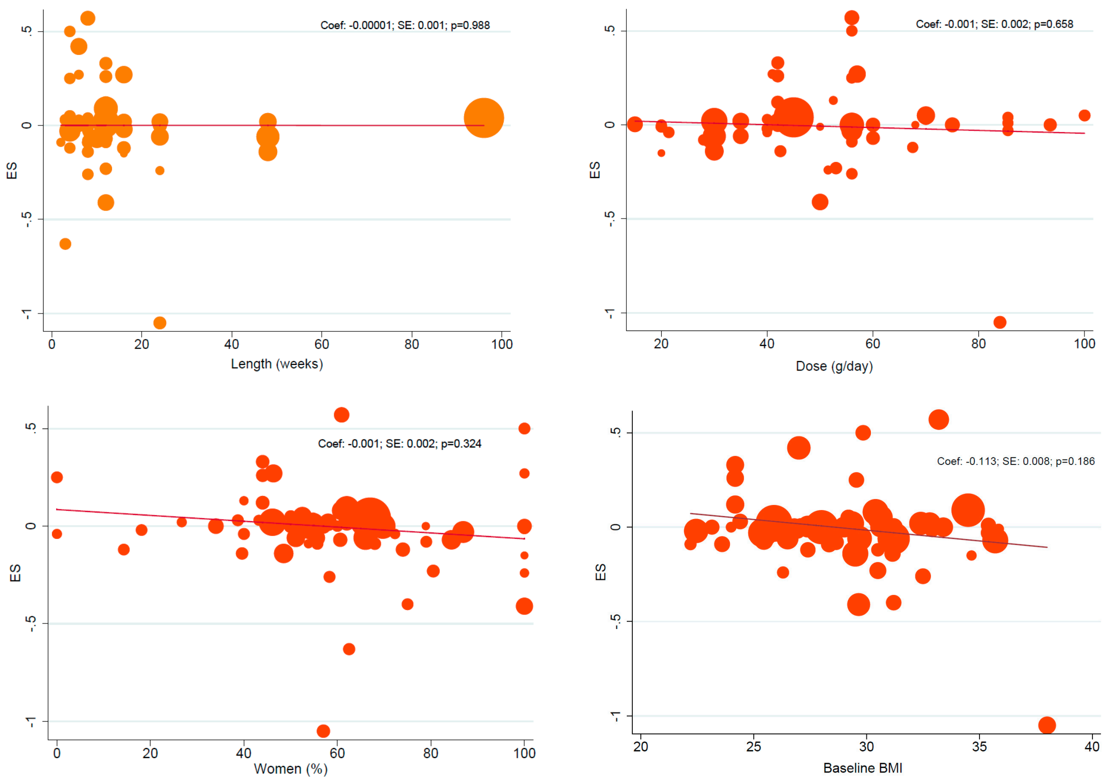

Waist circumference.

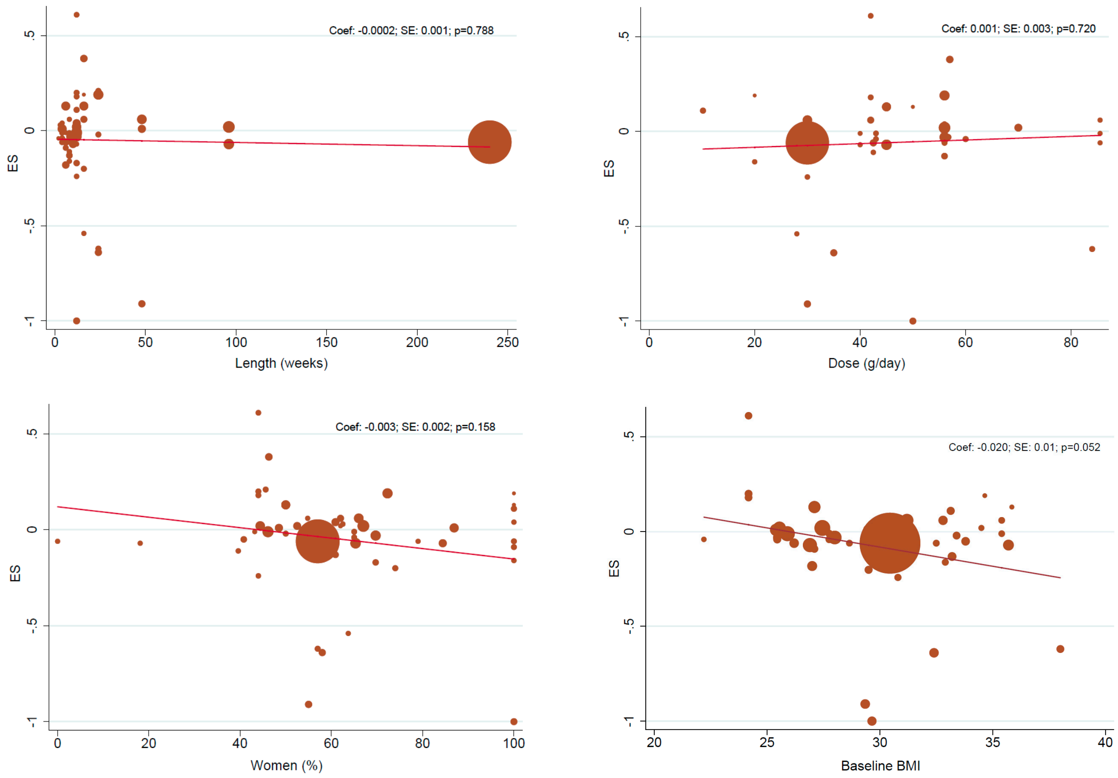

Body fat percentage.

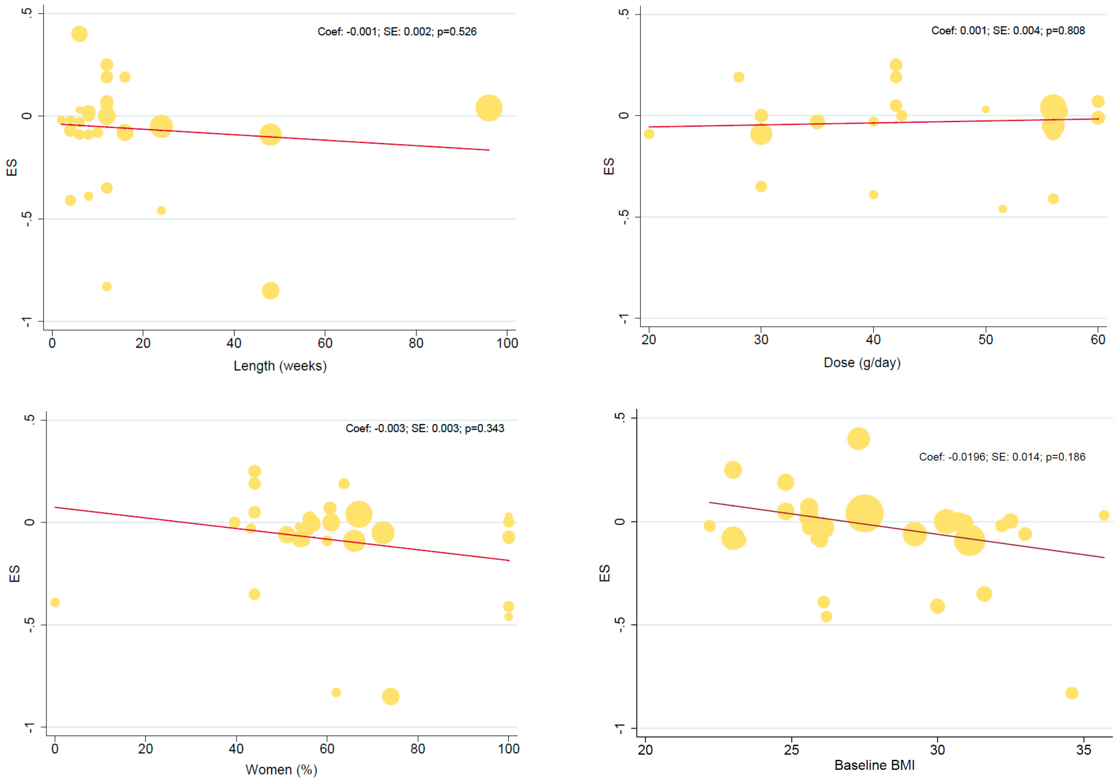

Figure S7. Funnel plots.

Body weight

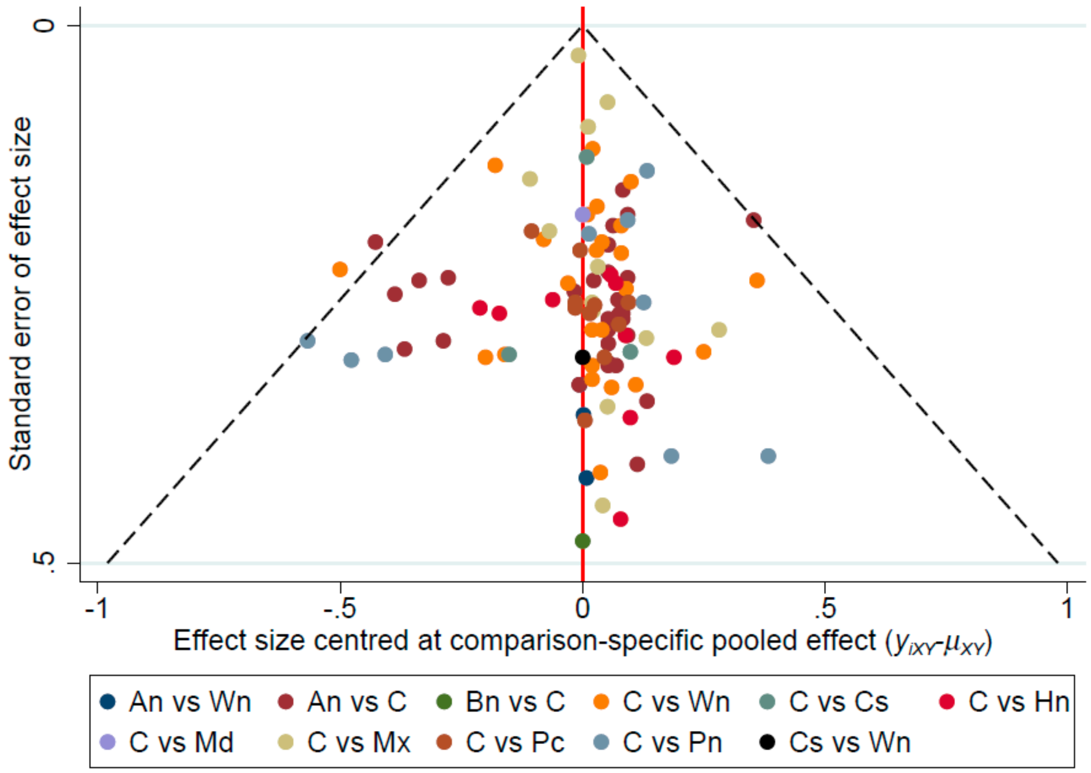

Body mass index

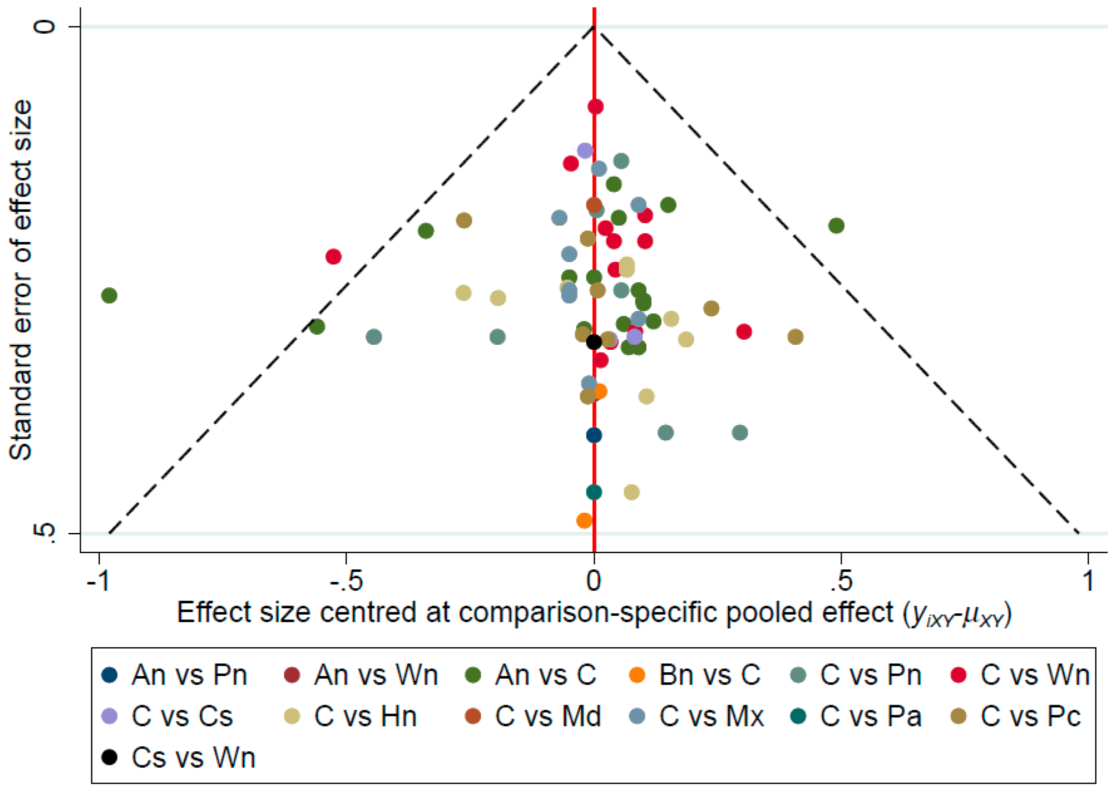

Waist circumference.

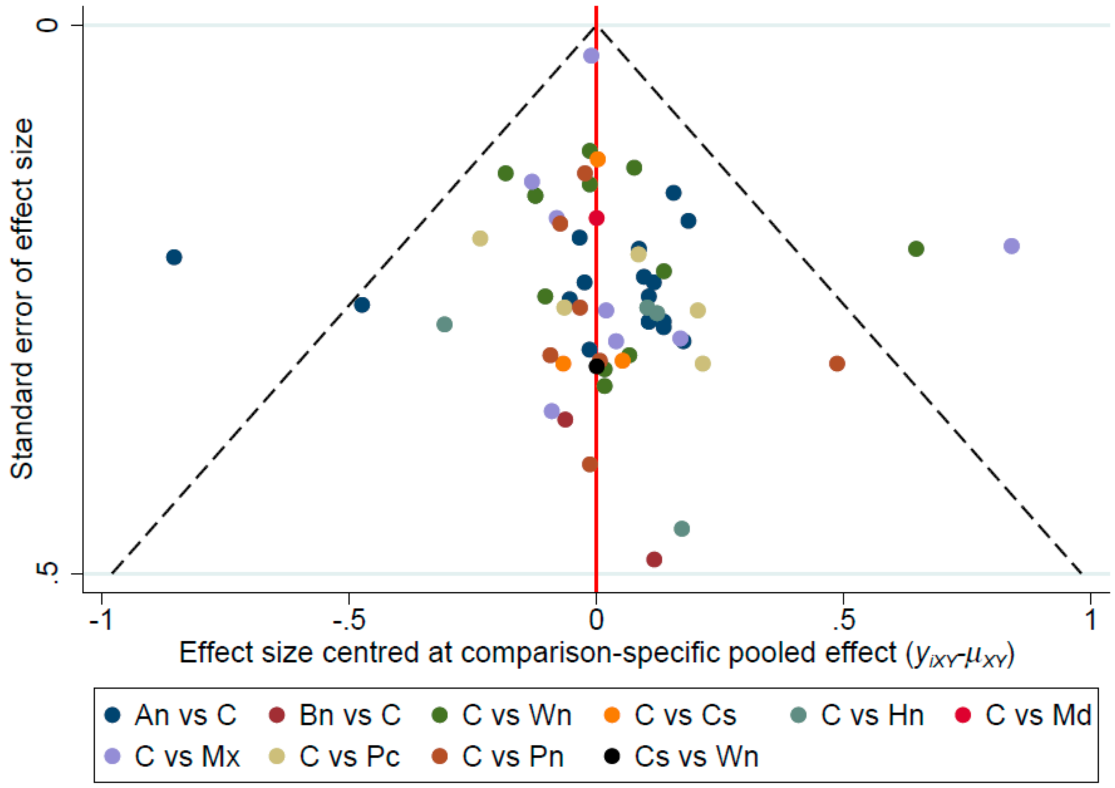

Body fat percentage

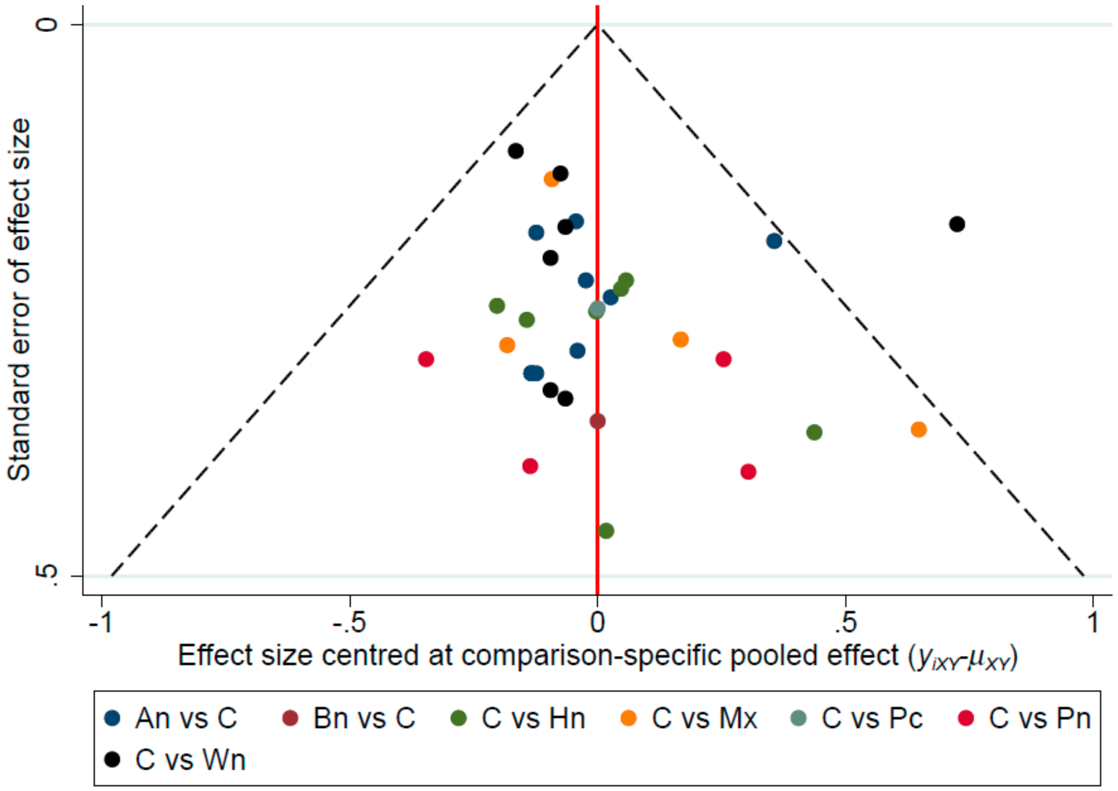

Figure S8. Risk of Bias assessment.

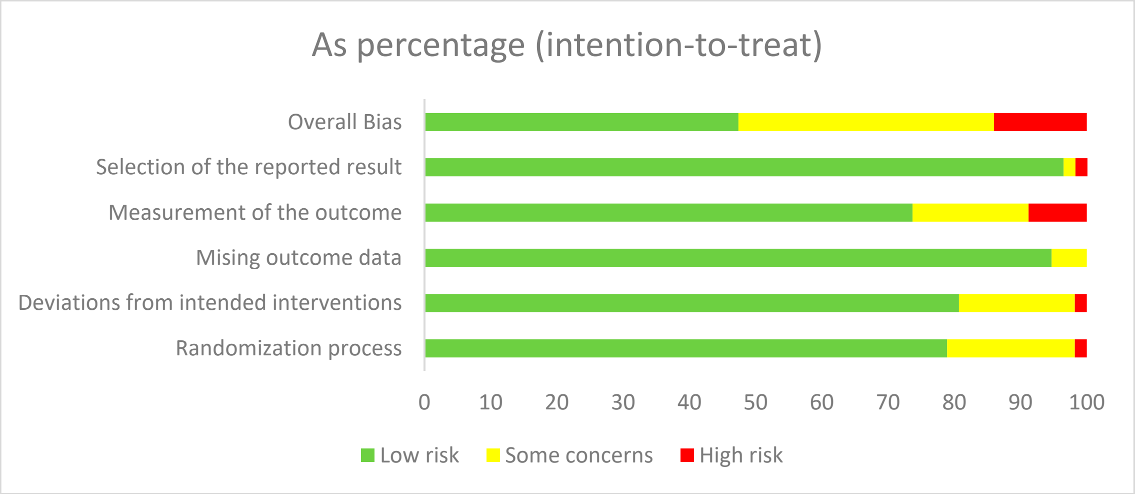

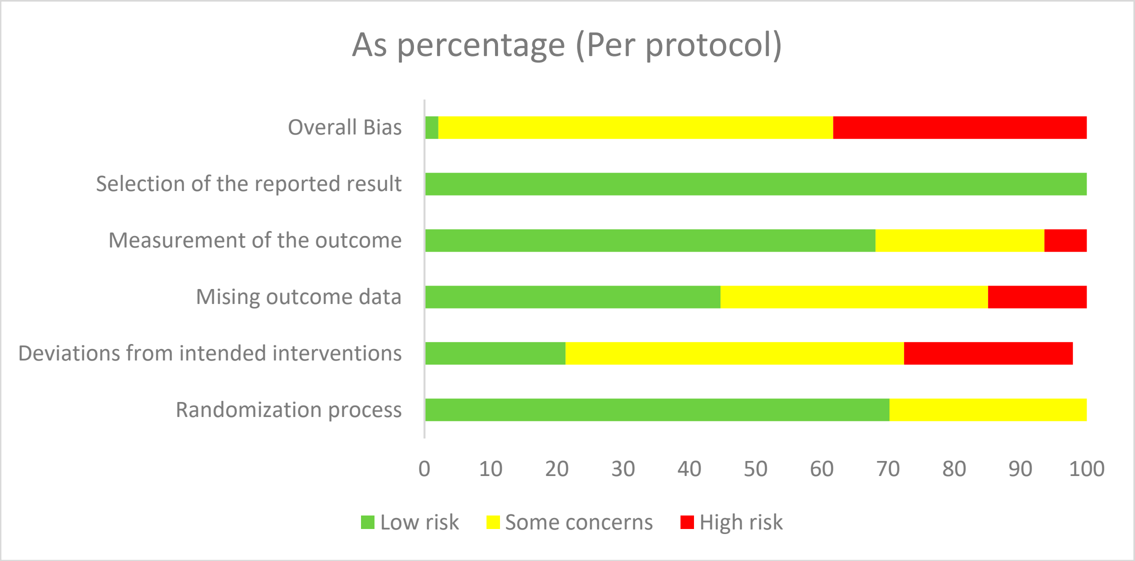

Supplement: Supplementary file 1 [file nutrients-13-02251-s001.zip › nutrients-1241018-supplementary.pdf]
